# International Multicenter Analysis of Brain Structure Across Clinical Stages of Parkinson's Disease

**DOI:** 10.1002/mds.28706

**Published:** 2021-07-20

**Authors:** Max A. Laansma, Joanna K. Bright, Sarah Al‐Bachari, Tim J. Anderson, Tyler Ard, Francesca Assogna, Katherine A. Baquero, Henk W. Berendse, Jamie Blair, Fernando Cendes, John C. Dalrymple‐Alford, Rob M.A. de Bie, Ines Debove, Michiel F. Dirkx, Jason Druzgal, Hedley C.A. Emsley, Gäetan Garraux, Rachel P. Guimarães, Boris A. Gutman, Rick C. Helmich, Johannes C. Klein, Clare E. Mackay, Corey T. McMillan, Tracy R. Melzer, Laura M. Parkes, Fabrizio Piras, Toni L. Pitcher, Kathleen L. Poston, Mario Rango, Letícia F. Ribeiro, Cristiane S. Rocha, Christian Rummel, Lucas S.R. Santos, Reinhold Schmidt, Petra Schwingenschuh, Gianfranco Spalletta, Letizia Squarcina, Odile A. van den Heuvel, Chris Vriend, Jiun‐Jie Wang, Daniel Weintraub, Roland Wiest, Clarissa L. Yasuda, Neda Jahanshad, Paul M. Thompson, Ysbrand D. van der Werf, The ENIGMA‐Parkinson's Study

**Affiliations:** ^1^ Department of Anatomy & Neurosciences, Amsterdam Neuroscience Amsterdam UMC, Vrije Universiteit Amsterdam Amsterdam the Netherlands; ^2^ Imaging Genetics Center, Mark and Mary Stevens Neuroimaging and Informatics Institute, Keck School of Medicine University of Southern California Marina del Rey California USA; ^3^ Faculty of Health and Medicine The University of Lancaster Lancaster UK; ^4^ Division of Neuroscience and Experimental Psychology, Faculty of Biology, Medicine and Health The University of Manchester, Manchester Academic Health Science Centre Manchester UK; ^5^ Department of Neurology Royal Preston Hospital Preston UK; ^6^ Department of Medicine University of Otago, Christchurch Christchurch New Zealand; ^7^ Department of Neurology, USC Stevens Neuroimaging and Informatics Institute University of Southern California Los Angeles California USA; ^8^ Laboratory of Neuropsychiatry IRCCS Santa Lucia Foundation Rome Italy; ^9^ GIGA‐CRC In Vivo Imaging University of Liège Liège Belgium; ^10^ Department of Neurology, Amsterdam Neuroscience Amsterdam UMC, University of Amsterdam Amsterdam the Netherlands; ^11^ Department of Medical Imaging University of Virginia Health System Charlottesville Virginia USA; ^12^ Neuroimaging Laboratory, Department of Neurology University of Campinas Campinas Brazil; ^13^ New Zealand Brain Research Institute Christchurch New Zealand; ^14^ School of Psychology, Speech and Hearing University of Canterbury Christchurch New Zealand; ^15^ Brain Research New Zealand ‐ Rangahau Roro Aotearoa, Centre of Research Excellence Auckland New Zealand; ^16^ Department of Neurology University Hospital Bern, Inselspital, University of Bern Bern Switzerland; ^17^ Department of Neurology and Center of Expertise for Parkinson & Movement Disorders, Donders Institute for Brain, Cognition and Behaviour Radboud University Nijmegen Medical Centre Nijmegen The Netherlands; ^18^ Centre for Cognitive Neuroimaging, Donders Institute for Brain, Cognition and Behaviour Radboud University Nijmegen Nijmegen the Netherlands; ^19^ Department of Radiology and Medical Imaging University of Virginia Charlottesville Virginia USA; ^20^ Lancaster Medical School Lancaster University Preston UK; ^21^ Department of Neurology CHU Liège Liège Belgium; ^22^ Department of Biomedical Engineering Illinois Institute of Technology Chicago Illinois USA; ^23^ Department of Clinical Neurosciences, Division of Clinical Neurology, Oxford Parkinson's Disease Centre, Nuffield University of Oxford Oxford UK; ^24^ Department of Psychiatry University of Oxford Oxford UK; ^25^ University of Pennsylvania Perelman School of Medicine Philadelphia Pennsylvania USA; ^26^ Department of Neurology & Neurological Sciences Stanford University Palo Alto California USA; ^27^ Excellence Center for Advanced MR Techniques and Parkinson's Disease Center, Neurology Unit, Fondazione IRCCS Cà Granda Maggiore Policlinico Hospital University of Milan Milan Italy; ^28^ Department of Medical Genetics University of Campinas Campinas Brazil; ^29^ Support Center for Advanced Neuroimaging (SCAN), University Institute of Diagnostic and Interventional Neuroradiology University Hospital Bern Bern Switzerland; ^30^ Department of Neurology, Clinical Division of Neurogeriatrics Medical University Graz Graz Austria; ^31^ Department of Neurology Medical University of Graz Graz Austria; ^32^ Psychiatry, Amsterdam Neuroscience Amsterdam UMC, Vrije Universiteit Amsterdam Amsterdam The Netherlands; ^33^ Department of Medical Imaging and Radiological Sciences Chang Gung University Taoyuan City Taiwan; ^34^ Department of Diagnostic Radiology Chang Gung Memorial Hospital, Keelung Branch Keelung City Taiwan; ^35^ Department of Psychiatry University of Pennsylvania Perelman School of Medicine Philadelphia Pennsylvania USA

**Keywords:** Parkinson's disease, MRI, brain, ENIGMA, disease severity

## Abstract

**Background:**

Brain structure abnormalities throughout the course of Parkinson's disease have yet to be fully elucidated.

**Objective:**

Using a multicenter approach and harmonized analysis methods, we aimed to shed light on Parkinson's disease stage‐specific profiles of pathology, as suggested by in vivo neuroimaging.

**Methods:**

Individual brain MRI and clinical data from 2357 Parkinson's disease patients and 1182 healthy controls were collected from 19 sources. We analyzed regional cortical thickness, cortical surface area, and subcortical volume using mixed‐effects models. Patients grouped according to Hoehn and Yahr stage were compared with age‐ and sex‐matched controls. Within the patient sample, we investigated associations with Montreal Cognitive Assessment score.

**Results:**

Overall, patients showed a thinner cortex in 38 of 68 regions compared with controls (*d*_max_ = −0.20, *d*_min_ = −0.09). The bilateral putamen (*d*_left_ = −0.14, *d*_right_ = −0.14) and left amygdala (*d* = −0.13) were smaller in patients, whereas the left thalamus was larger (*d* = 0.13). Analysis of staging demonstrated an initial presentation of thinner occipital, parietal, and temporal cortices, extending toward rostrally located cortical regions with increased disease severity. From stage 2 and onward, the bilateral putamen and amygdala were consistently smaller with larger differences denoting each increment. Poorer cognition was associated with widespread cortical thinning and lower volumes of core limbic structures.

**Conclusions:**

Our findings offer robust and novel imaging signatures that are generally incremental across but in certain regions specific to disease stages. Our findings highlight the importance of adequately powered multicenter collaborations. © 2021 The Authors. *Movement Disorders* published by Wiley Periodicals LLC on behalf of International Parkinson and Movement Disorder Society

## Graphical abstract

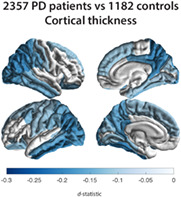

Parkinson's disease (PD) is the world's second most prevalent neurodegenerative disease. Apart from cardinal motor symptoms, patients may suffer from cognitive, neuropsychiatric, and autonomic dysfunction.^1^ Clinical features of PD are thought to arise in part from dysfunction of neural circuits, involving both cortical and subcortical regions.^2^ The use of neuroimaging to investigate macroscopic brain structural changes in PD may help in the understanding of patterns of the underlying pathology and potentially provide in vivo biomarkers of disease process and development.

Structural MRI of the brain allows for noninvasive assessment of cortical and subcortical morphology. Most imaging studies of PD report findings consistent with the atrophic process that underlies neurodegeneration, such as lower measures of subcortical volume and cortical thickness in PD compared with healthy controls.^3^ Reported atrophy patterns vary across studies in terms of location and effect size, and it is still poorly understood how disease severity relates to profiles of abnormal brain morphology.^3^, ^4^ The discrepancies may be explained, in part, by methodological factors, including small sample size for individual studies and differences in analysis methods. Heterogeneity with respect to demographics and clinical characteristics of the patient sample, regions of interest assessed, and algorithms used for segmentation and parcellation (eg, atlas‐based versus voxel‐ or vertex‐based) may also produce differences in reported findings, which in turn complicate the comparability of study outcomes.

Large‐scale collaborations, such as the Enhancing Neuroimaging through Meta‐Analysis (ENIGMA) consortium, have been initiated to overcome these limitations by harmonizing data processing and analysis across studies and aggregating information across multiple samples worldwide.^5^

The ENIGMA‐PD Working Group is an international initiative set up to identify imaging signatures of pathology in PD and factors that influence them. In the largest study on PD brain morphology to date, we report differences in regional cortical thickness, cortical surface area, and subcortical volume between PD patients and healthy control subjects and provide clinicomorphological correlates, taking into account disease severity, age, and sex.

## Methods

### Samples

Data were collected between September 2016 and December 2019. We analyzed T1‐weighted MRI scans from 19 sites from 20 countries (Figure S1) comprising 2357 PD patients and 1182 control subjects. Clinical information from the PD subjects included Hoehn and Yahr (HY) stage,^6^ illness duration, and Montreal Cognitive Assessment (MoCA) score.^7^ Every site also supplied scans of healthy controls, if available, with identical MR imaging parameters. Individual‐site inclusion/exclusion criteria are provided in Table S1a. The 43 samples of PD patients and controls provided were defined as “cohorts,” such that sites may contribute multiple cohorts from separate testing environments. In particular, the Parkinson's Progression Markers Initiative collects data across multiple centers,^8^ and these were treated as independent cohorts. Disease severity was assessed using HY stage, ranging from 1 to 5, from HY1, unilateral motor impairment, to HY5, confinement to bed or wheelchair. The modified HY classification, which includes intermediate increments of 1.5 and 2.5 to complement stage 2,^9^ was used in 12 cohorts. We regrouped the cases so that HY1.5 (n = 83) and HY2.5 (n = 169) patients were included in the HY2 group. The HY4 (n = 66) and HY5 (n = 17) patient groups were merged. The nearest neighbor‐matching procedure, featured in the MatchIt software package for R,^10^ selected an age‐ and sex‐balanced subsample of controls for each HY group based on propensity score matching with replacement.

### Image Acquisition and Processing

Structural brain MRI scans were obtained with a 3‐dimensional gradient‐echo T1‐weighted sequence. Site‐specific parameters are summarized in Table S1b. Contributing sites processed their data locally using standardized ENIGMA protocols for harmonization and quality control (see http://enigma.ini.usc.edu/protocols/imaging-protocols/). Regional cortical thickness, cortical surface area, and subcortical volume metrics were extracted from the brain images using FreeSurfer 5.3. For each subject, per hemisphere, FreeSurfer parcellated 34 cortical regions of interest (ROIs) based on the Desikan‐Killiany atlas, and 8 subcortical ROIs.^11^, ^12^ Poorly parcellated regions were excluded from the statistical analysis, in accordance with the standardized protocols. All collaborators in our Working Group granted permission to share individual participant‐derived data, including demographic and clinical characteristics and FreeSurfer‐derived measures. All sites provided anonymized data with ethical approval from their local ethics committees and institutional review boards.

### Analysis of Cortical and Subcortical Properties

Between‐group differences were assessed using multivariable linear mixed‐effects regression on the pooled means of regional cortical thickness (mm), regional and total cortical surface area (mm^2^), regional subcortical volume, and intracranial volume (ICV; mm^3^). Independent variables diagnosis, age, sex, and ICV were used as fixed factors and cohort was included as a random intercept.

The main analysis examined differences between all patients and controls using model 1a (ROI ~ diagnosis + age + sex + ICV + cohort) for subcortical volume and regional cortical surface area, model 1b (ROI ~ diagnosis + age + sex + cohort) for cortical thickness and total cortical surface area, and model 1c for ICV (ICV ~ diagnosis + sex + cohort). Omitting ICV in the thickness model is consistent with previous research on nuisance factors.^13^ Differences between patients grouped by HY stage and age‐ and sex‐matched controls were assessed using model 2a (ROI ~ diagnosis[HY_N_] + ICV + cohort) for subcortical volume and regional cortical surface area and model 2b (ROI ~ diagnosis[HY_N_] + cohort) for cortical thickness and total cortical surface area. The d‐statistic appropriate for mixed‐effects models was estimated to quantify the effect size of the differences.^14^ The percentage difference of patients from controls was calculated using the least‐squares group means of the outcome measure.

In addition, we used a linear mixed‐effects regression model to examine within‐group associations between the morphometric measures and cognitive ability, and illness duration, incorporating model 1a for subcortical volume and cortical surface area and model 1b for cortical thickness. The *r*‐statistic appropriate for mixed‐effects models was reported as the effect size.^14^ To determine how representative the MoCA subgroup was for the complete PD sample, we performed a differential analysis between the PD group with available MoCA scores and the control group (supplementary material).

Significant results that passed Bonferroni correction for multiple comparisons were reported (ie, *P* = 0.05 divided by number of ROIs of each outcome measure); ICV is calculated differently from the subcortical volume measure and was treated as a separate outcome measure.^15^

## Results

### Complete Sample

**FIG. 1:**
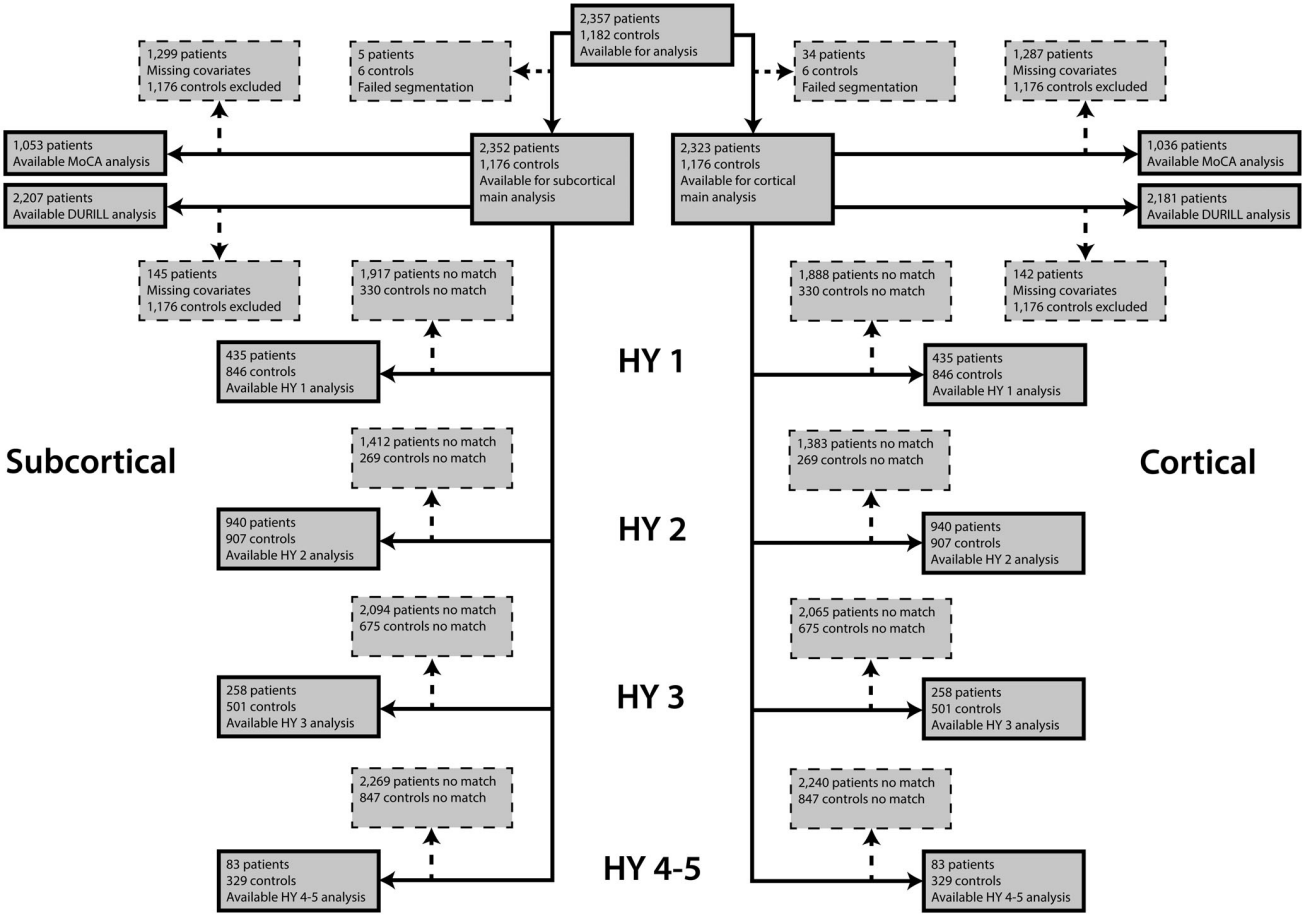
Flowchart of data inclusion. Schematic overview of derived subcortical and cortical samples for each analysis. HY, Hoehn & Yahr; med, medication; MoCA, Montreal Cognitive Assessment.

Data flow for each analysis is depicted in Figure 1 and participant data in Table 1. There was a significant difference in age, *t*_1947.3_ = 9.9, *P* < 0.001, and sex, χ^2^(1, n = 3539) = 35.2, *P* < 0.001, between patients and controls in the complete sample.

**TABLE 1:** Demographic characteristics of 2357 Parkinson's disease patients and 1182 controls stratified by cohort

Site	Cohort	n		Age (y), mean (SD)		Female %		DURILL (y), mean (SD)	
		HC	PD	HC	PD	HC	PD	HC	PD
Amsterdam	Amsterdam I	44	138	56.5 (9.48)	63.1 (10.81)	39	38	NA	2.1 (3.39)
	Amsterdam II	0	61	NA	62.5 (7.08)	NA	39	NA	5.3 (3.54)
Bern	BE I	23	52	54.1 (9.78)	62.9 (10.38)	30	52	NA	12.4 (4.29)
	BE II	30	3	68.2 (4.59)	59.7 (6.66)	70	67	NA	11.3 (7.57)
Campinas	UNICAMP	138	110	58.9 (7.91)	59.9 (10.2)	63	34	NA	7.3 (6.41)
Chang Gung	CGU	223	327	61 (7.28)	60.1 (9.63)	54	43	NA	8.7 (6.33)
Charlottesville	UVA I	0	116	NA	63.7 (8.52)	NA	28	NA	9.7 (5.09)
	UVA II	0	37	NA	62.4 (9.59)	NA	14	NA	8.7 (3.64)
	UVA III	0	24	NA	70.8 (6.77)	NA	29	NA	7.7 (3.23)
Christchurch	PDNZ	39	209	67.5 (8.52)	69.4 (7.77)	33	26	NA	5.7 (5.57)
Donders	Donders	23	59	62.7 (10.29)	60.8 (10.07)	48	44	NA	4.4 (3.79)
Graz	PROMOVE/ASPS I	124	100	63.4 (10.07)	63.2 (10.15)	27	29	NA	4.7 (4.77)
	PROMOVE/ASPS II	0	23	NA	64 (9.9)	NA	22	NA	4 (5.69)
Liege	Liege I	33	30	65.8 (4.29)	65.9 (6.61)	45	37	NA	7.2 (5.32)
	Liege II	43	45	64.8 (8.33)	66.9 (8.24)	49	44	NA	6 (3.93)
Milan	Milan	10	44	53.3 (10.53)	57.8 (7.71)	70	32	NA	11.4 (3.38)
NEUROCON	NEUROCON	15	27	66.7 (11.74)	68.7 (10.55)	80	37	NA	NA
NW‐England	NW‐England I	22	32	70 (7.27)	69.9 (8.58)	45	19	NA	6.8 (4.42)
	NW‐England II	13	14	64.6 (4.13)	65 (5.67)	38	29	NA	9.2 (6.02)
ON Japan	ON Japan	15	30	63.3 (5.25)	67.6 (6.81)	53	57	NA	NA
Oxford	Oxford DISCOVERY	57	115	65.6 (8.2)	63.9 (10.05)	39	36	NA	2.3 (1.58)
Pennsylvania	UDALL/U19	11	112	70.1 (5.86)	66.4 (7.87)	55	32	NA	7.3 (5.48)
PPMI	PPMI 1‐21	163	347	63.6 (16.73)	62.9 (8.19)	36	35	NA	0.6 (0.52)
Rome SLF	Rome SLF	125	239	36.6 (10.63)	62.7 (10.19)	41	37	NA	4.9 (4.17)
Stanford	Stanford	11	44	65.6 (6.47)	68.6 (8.49)	82	50	NA	5.6 (3.44)
Tao Wu	Tao Wu	20	19	64.8 (5.58)	65 (4.45)	40	47	NA	5.3 (4)
Total		1182	2357	59.4 (12.31)	63.4 (9.77)	46	36	NA	5.5 (5.47)

NA, not available; n, sample size; HC, healthy control; PD, Parkinson's disease; PPMI, Parkinson's Progression Markers Initiative; SD, standard deviation; DURILL, duration of illness; MoCA, Montreal Cognitive Assessment.

#### Cortical Thickness, Cortical Surface Area, and ICV

**FIG. 2:**
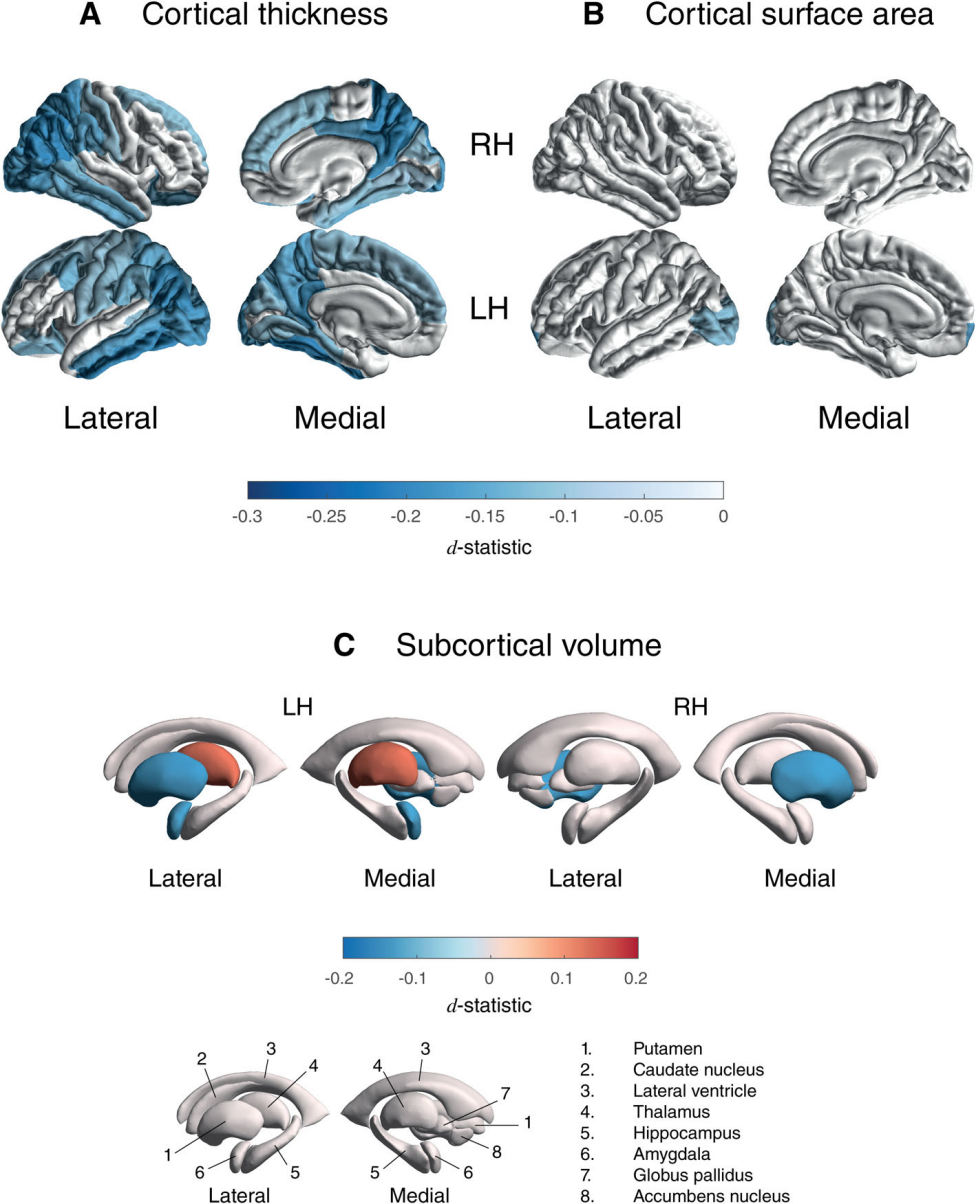
Cortical thickness, cortical surface area, and subcortical volume group differences for Parkinson's disease patients versus controls. *D*‐statistic effect size estimates for mean differences in (**A**) cortical thickness, (**B**) cortical surface area, and (**C**) subcortical volume. A negative *d‐*value indicates smaller measurements in Parkinson's disease patients. Cortical regions with *P* < 7.35 × 10^−4^ (ie, 0.05/68 ROIs) are depicted in the heat‐map colors. Subcortical regions with *P* < 3.13 × 10^−3^ (ie, 0.05/16 ROIs) are depicted as in the heat‐map colors. RH, right hemisphere; LH, left hemisphere; ROI, region of interest, L, left; R, right; n., nucleus. [Color figure can be viewed at wileyonlinelibrary.com]

PD patients showed a significantly thinner cortex compared with controls in 20 of 34 left‐hemisphere ROIs (*d*_max_ = −0.20, −1.79%; *d*_min_ = −0.10, −0.78%) and 18 of 34 right‐hemisphere ROIs (*d*_max_ = −0.19, −1.87%; *d*_min_ = −0.09, −0.88%; Fig. 2A and Table S2a). Differences appeared symmetrical in 16 ROIs. All but the right parahippocampal gyrus (*P =* 0.0891), left pars orbitalis (*P =* 0.0572), and left superior frontal gyrus (*P =* 0.0600) remained significant when corrected for ICV. Surface areas of the left frontal pole (*d* = −0.17, −3.08%) and lateral occipital cortex (*d* = −0.12, −1.48%) were significantly smaller in patients (Fig. 2B and Table S2b). We found no differences for total surface area between patients and controls (*P* = 0.5272). Patients had a higher ICV than controls (*P* = 0.010, *d* = 0.08, 0.98%).

#### Subcortical Volume

PD patients showed a significantly larger left thalamus (*d* = 0.13, 1.79%), smaller putamen bilaterally (*d*_left_ = −0.14, −2.03%; *d*_right_ = −0.14, −2.01%), and a smaller left amygdala (*d* = −0.13, −2.27%), compared with controls (Fig. 2C and Table S2c).

### HY Stages

The matching procedure selected 435 stage 1 patients (846 controls), 940 stage 2 patients (907 controls), 258 stage 3 patients (501 controls), and 83 stage 4 and 5 patients (329 controls) for the analyses (Table S8a–d). Controls partially overlapped across stages (Table S9). Mann–Whitney tests revealed significant differences in illness duration and MoCA score among all HY groups (Table S10).

#### Cortical Thickness

**FIG. 3:**
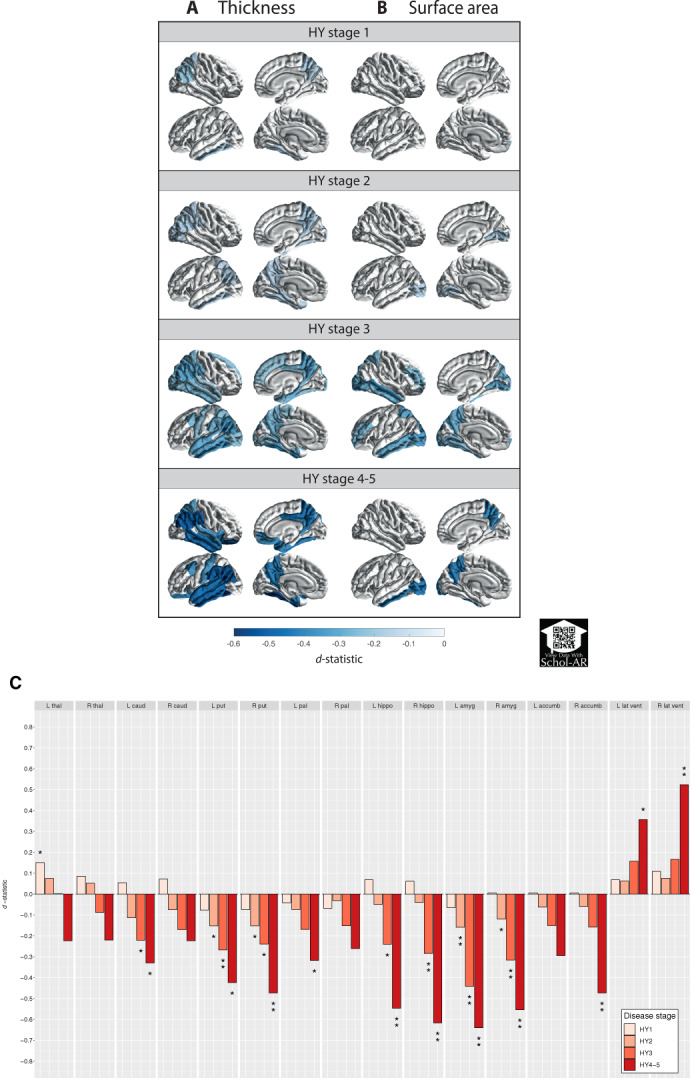
Cortical thickness, cortical surface area, and subcortical volume group differences for Parkinson's disease groups, at different Hoehn & Yahr stages versus age‐ and sex‐matched controls. *D*‐statistic effect size estimates for mean differences in (**A**) cortical thickness, (**B**) cortical surface area, and (**C**) subcortical volume. Cortical regions with *P* < 7.35 × 10^−4^ (ie, 0.05/68 ROIs) are depicted in the heat‐map colors. *Subcortical regions with *P* < 3.13 × 10^−3^ (ie, 0.05/16 ROIs); **structures with *P* < 6.25 × 10^−5^ (ie, 0.001/16 ROIs). Scan the QR code to download the Schol‐AR app and aim your camera at Figure 3 to see an augmented reality version of the supplementary videos. ROI, region of interest; L, left; R, right; thal, thalamus; amyg, amygdala; caud, caudate nucleus; hippo, hippocampus; accumb, accumbens nucleus; put, putamen; pal, globus pallidus; lat vent, lateral ventricle. [Color figure can be viewed at wileyonlinelibrary.com]

A summary of thickness results is shown in Figure 3A and Table S3a and complete results in Table S4a–d. Compared with controls, HY1 patients showed a thinner left fusiform (*d* = −0.16, −1.31%) and inferior temporal cortex (*d* = −0.18, −1.43%), right precuneus (*d* = −0.17, −1.46%), and inferior (*d* = −0.22, −1.91%) and superior (*d* = −0.17, −1.71%) parietal cortex. HY2 patients showed a thinner cortex in 8 left hemisphere ROIs (*d*_max_ = −0.17, −2.53%; *d*_min_ = −0.13, −1.22%) and 7 right hemisphere ROIs (*d*_max_ = −0.18, −1.97%; *d*_min_ = −0.13, −1.34%). HY3 patients showed a thinner cortex in 15 left hemisphere ROIs (*d*_max_ = −0.37, −3.67%; *d*_min_ = −0.21, −2.21%) and 17 right hemisphere ROIs (*d*_max_ = −0.33, −4.74%; *d*_min_ = −0.17, −1.89%). HY4‐5 patients showed a thinner cortex in 14 left hemisphere ROIs (*d*_max_ = −0.58, −5.24%; *d*_min_ = −0.34, −2.88%) and 15 right hemisphere ROIs (*d*_max_ = −0.52, −5.75%; *d*_min_ = −0.31, −3.19%). When corrected for ICV, all ROIs remained significant, except for the right temporal pole (*P =* 0.0512) in HY3 and the right isthmus cingulate gyrus (*P =* 0.0744) in HY4‐5.

#### Cortical Surface Area

Compared with controls, HY1 patients showed a smaller surface area of the left frontal pole (*d* = −0.22, −3.93%; Fig. 3B, Tables S3b and S4e–h). HY2 patients showed a smaller surface area of the lingual cortex (*d*_left_ = −0.15, −2.32%; *d*_right_ = −0.17, −2.57%), left lateral occipital cortex (*d* = −0.15, −1.99%), and right pericalcarine cortex (*d* = −0.17, −3.03%). HY3 patients showed a smaller surface area in 9 left hemisphere ROIs (*d*_max_ = −0.32, −5.82%; *d*_min_ = −0.25, −3.40%) and 7 right hemisphere ROIs (*d*_max_ = −0.35, −4.72%; *d*_min_ = −0.25, −3.11%). HY4‐5 patients combined showed a smaller surface area of the precuneus (*d*_left_ = −0.42, −5.10%; *d*_right_ = −0.40, −4.79%) and left inferior temporal (*d* = −0.40, −5.73%) and lateral occipital cortex (*d* = −0.42, −5.50%). We found no differences for total surface area between patients and controls for all HY stages (Table S12).

#### Subcortical Volume

Results of the subcortical analysis are depicted in Figure 3C and Tables S3c, and S4i–l. Compared with controls, HY1 patients showed a significantly larger left thalamus (*d* = 0.15, 2.15%). HY2 patients showed smaller bilateral amygdalae (*d*_left_ = −0.16, −2.99%; *d*_right_ = −0.12, −2.28%) and smaller putamen (*d*_left_ = −0.15, −2.45%; *d*_right_ = −0.15, −2.49%). At HY3, patients showed smaller amygdalae (*d*_left_ = −0.44, −8.63%; *d*_right_ = −0.32, −6.28%), putamen (*d*_left_ = −0.27, −4.67%; *d*_right_ = −0.24, −4.16%), hippocampi (*d*_left_ = −0.24, −3.43%; *d*_right_ = −0.28, −4.27%), and left caudate nucleus (*d* = −0.22, −3.72%). Finally, HY4‐5 patients showed smaller amygdalae (*d*_left_ = −0.64, −11.60%; *d*_right_ = −0.55, −9.98%), hippocampi (*d*_left_ = −0.55, −6.82%; *d*_right_ = −0.61, −8.11%), putamen (*d*_left_ = −0.42, −6.43%; *d*_right_ = −0.47, −7.47%), left caudate nucleus (*d* = −0.33, −5.20%), globus pallidus (*d* = −0.32, −6.97%), and right accumbens (*d* = −0.47, −12.45%). The lateral ventricles were larger in PD (*d*_left_ = 0.36, −18.42%; *d*_right_ = 0.52, −27.80%).

#### Post Hoc HY Side‐by‐Side Comparison

Comparisons between HY increments, using model 1, revealed mainly significantly thinner cortical and smaller subcortical ROIs in HY3 compared with HY2 that overlapped with the case–control findings (Table S7a–i). Comparisons between HY groups, also corrected for illness duration, revealed largely consistent volume differences of both hippocampi and left amygdala (Table S11a–c).

### MoCA

A total of 1057 patients had MoCA scores available for analysis (Table S12), including 425 patients (40.2%) with cognitive impairment (ie, MoCA < 26), of whom 88 patients (8.3%) had dementia (ie, MoCA < 21).

#### Cortical Thickness and Surface Area

**FIG. 4:**
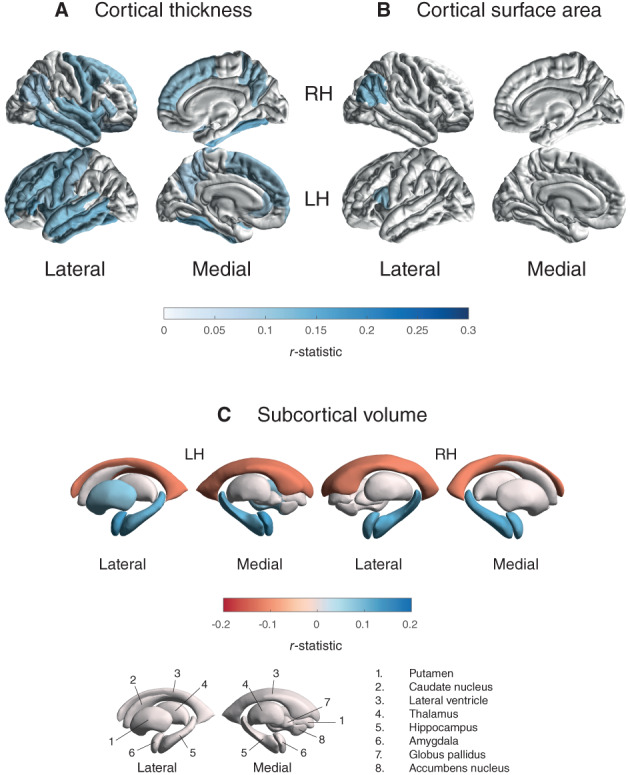
Cortical thickness, cortical surface area, and subcortical volume findings for the MoCA regression. *R*‐statistic estimates for the associations with (**A**) cortical thickness, (**B**) cortical surface area, and (**C**) subcortical volume. Cortical regions with *P* < 7.35 × 10^−4^ (ie, 0.05/68 ROIs) are depicted in the heat‐map colors. Subcortical regions with *P* < 3.13 × 10^−3^ (ie, 0.001/16 ROIs) are depicted in the heat‐map colors. Higher MoCA scores denote better cognitive performance. RH, right hemisphere; LH, left hemisphere; ROI, region of interest; n., nucleus. [Color figure can be viewed at wileyonlinelibrary.com]

Thickness results are depicted in Figure 4A and Tables S5a, and S15a. The analysis revealed a significant positive correlation between MoCA score and cortical thickness in 15 ROIs in the left hemisphere (*r*_max_ = 0.14; *r*_min_ = 0.09) and 13 ROIs in the right hemisphere (*r*_max_ = 0.14; *r*_min_ = 0.08). All ROIs but the left precuneus and right transverse temporal gyrus remained significant when corrected for illness duration. Surface area results are shown in Figure 4B and Tables S5b, and S15b. We found a significant positive correlation between MoCA score and cortical surface area in the left pars opercularis (*r* = 0.11) and the right inferior parietal cortex (*r* = 0.12). This remained significant when corrected for illness duration. MoCA results for PD patients versus control results are depicted in Table S6a,b.

#### Subcortical Volume

Volume results are depicted in Figure 4C and Tables S5c, and S15c. The analysis revealed a significant positive correlation between MoCA score and the hippocampus (*r*_left_ = 0.11; *r*_right_ = 0.12), amygdala (*r*_left_ = 0.13; *r*_right_ = 0.11), and left putamen (*r* = 0.08) volumes. In addition, we found a negative correlation between MoCA score and lateral ventricular volume bilaterally (*r*_left_ = −0.12; *r*_right_ = −0.11). All ROIs but the left putamen remained significant when corrected for illness duration. The MoCA results for patients versus controls are depicted in Table S6c.

### Illness Duration

A total of 2211 patients had illness duration scores available for analysis (Table S13).

#### Cortical Thickness and Surface Area

Thickness results are depicted in Table S14a. The analysis revealed a significant negative correlation between illness duration score and thinning of the precuneus (*r*_left_ = −0.06; *r*_right_ = −0.06), and left inferior (*r* = −0.06) and right (*r* = −0.06) superior parietal cortex. Surface area results are shown in Table S14b. We found a significant negative correlation between illness duration score and cortical surface area in the medial orbitofrontal cortex (*r* = −0.08). Results uncorrected for age are depicted in Table S14d,e.

#### Subcortical Volume

Volume results are depicted in Table S14c. The analysis revealed a significant negative correlation between illness duration score and the thalamus (*r*_lef*t*_ = −0.05; *r*_right_ = −0.06), amygdala (*r*_left_ = −0.11; *r*_right_ = −0.10), hippocampus (*r*_left_ = −0.06; *r*_right_ = −0.06), caudate (*r*_left_ = −0.10; *r*_right_ = −0.08), left putamen (*r* = −0.07), and left accumbens (*r* = −0.06) volumes. Results uncorrected for age are depicted in Table S14f.

## Discussion

### Main Findings

In this largest collaborative MRI study on PD to date, we found lower cortical thickness, on average, in patients compared with controls across all HY disease stages, more pronounced with higher disease severity. In the subcortex, a larger left thalamus in patients in stage 1 was followed by smaller putamen and amygdala bilaterally in stage 2 and onward. Late‐stage patients showed smaller hippocampus, left caudate nucleus, left globus pallidus, and right accumbens and larger lateral ventricles. Finally, we found that poorer cognitive performance was associated with widespread cortical atrophy and volume loss in core limbic structures.

### HY and Disease Staging

HY stage reliably tracks disease progression,^16^ although the relationship between the development of motor and cognitive symptoms has not been fully elucidated. Generally, both domains tend to worsen during the disease course, with a dementia prevalence up to 80% in the final stages of PD, in addition to severe movement disabilities.^17^ Our cortical and subcortical findings are strongly in line with an ongoing neurodegenerative process; each HY increment largely replicates the previous stage with additional implicated regions, emphasized by longer illness duration and poorer cognitive performance in patients. Furthermore, there was notable overlap in the implicated cortical and subcortical regions in illness duration and HY stage analysis, including parietal, striatal, and limbic structures. This is largely compatible with earlier work on the progression and propagation of atrophy in early PD except for the frontal cortex, which we found to be spared until later HY stages.^18^ Possibly, deformation‐based morphometry is more sensitive to capture these differences, whereas nuances may alternatively be driven by milder atrophy subtypes.^19^ The small effect sizes reflect the subtle differences that may be difficult to capture in smaller, underpowered studies. Notably, the stringent statistical method we used for better model fit is known to yield lower effect size estimates.^20^

### Cognitive Features

A thinner posterior and temporal cortex has been linked to cognitive impairment in the early symptomatic stages of PD.^21^ Indeed, we found that poorer cognition was associated with thinning in the parietal and inferior temporal regions, contingent on the thinner cortices, as demonstrated in HY1 and HY2 patients. However, we may assume the vast majority of early‐stage patients were cognitively normal,^22^ which would fit the notion that temporal and parietal degeneration may precede cognitive decline.^21^ In addition, the implication of the occipital cortex may relate to compromised visual functions in early‐stage PD patients.^23^ The diffuse pattern of thinner cortices alongside smaller hippocampi and amygdalae in HY3 patients agrees with the more advanced symptomatic stages associated with PD dementia^24^; we found those regions accordingly linked to worse cognitive performance. Patients in the final stages showed enlarged lateral ventricles, highlighting the severe atrophy in surrounding and adjacent structures such as the hippocampus. The spared occipital cortex in HY4‐5 contrasting HY2‐3 may be a surprising finding, because previous studies demonstrated gradual worsening of cholinergic denervation in this region, associated with cognitive decline and the appearance of dementia.^25^ It is possibly explained by the relatively small HY4‐5 sample size and dementia‐specific exclusion criteria related to 13 patients (15%), which together may have nuanced group differences. Interestingly, patterns associated with cognitive decline appeared largely independent of illness duration, possibly denoting the variable rate of cognitive symptom progression in PD.^24^

### HY and Relation to Staging Theories

An estimated 80% of striatal dopaminergic neurons are lost at the time of motor symptom onset in PD.^26^ Dysfunction of the nigrostriatal pathway is associated with motor symptoms and leads to reduced activity in the putamen.^27^ We observed lower putamen volumes, indicative of early abnormal atrophy in PD; the symmetry in HY2 corresponds to the transition from unilateral to bilateral motor impairment. Striatal degeneration is further highlighted by atrophy of the caudate nucleus in HY3 and onward. The globus pallidus appeared robust to volume loss until the final stages, contrasting with the role of pallidal dopamine depletion in tremor in PD.^28^ It should be noted, however, that the globus pallidus notoriously shows poor contrast on T1‐weighted scans,^29^ hampering adequate segmentation. Perhaps counterintuitively in view of a neurodegenerative disease, the volume of the left thalamus was higher in HY1 patients versus controls. Previous studies have reported local shape abnormalities of the thalamus in PD patients to suggest both atrophic and hypertrophic subregions.^30^, ^31^ It is proposed that the initial hypertrophy may be the result of hyperactivity in the cerebellothalamic circuit, which is thought to underlie Parkinson's tremor.^28^

According to Braak's staging model,^32^ Lewy body pathology spreads in an ascending fashion from brain stem regions toward the subcortex, finally reaching the neocortex through the mesocortex. Clinical symptoms manifest around Braak stage 4–5, when first limbic and then mesocortical structures become affected. Similarly, in this study the bilateral amygdalae are affected first at an early symptomatic stage, with reduced bilateral hippocampal volumes and thinner entorhinal, parahippocampal, and posterior cingulate regions in subsequent stages, denoting the transition to the neocortex. The ascending propagation of pathology as proposed by Braak is challenged by our finding of posterior cortical implication at an early stage, suggesting that neocortical degeneration may occur at least parallel to the onset of subcortical degeneration. The simultaneous development of pathology in multiple systems has been incorporated in alternative staging models and may offer a more comprehensive theory on the neurobiology of PD that better accounts for individual differences in manifestation, onset, and progression of motor and nonmotor symptoms such as cognitive impairment.^33^, ^34^ For example, unlike the frontostriatal dopaminergic circuits, the posterior cortex is heavily innervated by cholinergic projections, emphasizing the deterioration of distinct systems. Early‐stage dysfunction in the posterior cortex in PD patients has been linked to a phenotype showing rapid cognitive decline and conversion to dementia.^33^ However, we note that MRI‐derived findings should be cautiously interpreted in the context of α‐synuclein propagation.

### ICV and Brain Size

The larger ICV found in PD patients suggests that cranial overgrowth might be a risk factor for the disease, supported by earlier research demonstrating a shared genetic background between ICV and the risk of PD.^35^ Becaue of the congruent maturation of the brain and cranium and the unchanged cranial size through adulthood, ICV is considered a stable proxy for “maximal attained brain size.”^35^ It could be hypothesized that PD patients have a larger maximal attained brain size compared with healthy individuals, which would be a relevant early‐life marker. In the absence of premorbid data, we have evidence to suggest there is no difference in cerebral size, as measured by total cerebral surface area, between PD patients and healthy controls, despite early indications of abnormal atrophy in patients. Investigation of brain size in a premorbid group, such as patients suffering from prodromal REM‐sleep behavior disorder, could provide further insight into the possible role of brain overgrowth in PD.

### Limitations

The use of cross‐sectional data does not allow us to make clear inferences on atrophy patterns and disease progression as with a longitudinal design. The HY scale also does not encompass the variety of nonmotor symptoms that contribute to disease severity and progression. Moreover, because of the retrospective nature of data collection, some sites had specific inclusion/exclusion criteria related to psychiatric illness, cognitive impairment, and dementia, which may have made our sample less representative of the patient population, especially in the later HY stages, when these symptoms are more prevalent and severe. Nevertheless, we demonstrated that both longer illness duration and poorer cognitive performance were associated with each HY increment. Although the MoCA subgroup was representative of the full sample in terms of demographics, there may be hidden confounding clinical or environmental parameters influencing these results not picked up by our limited sample.

## Conclusions

To conclude, in this large multinational sample of PD patients versus healthy controls, we found widespread structural brain abnormalities on the cortical and subcortical level that may shed new light on the pathophysiology and progression of PD. The cortical and subcortical findings are strongly in line with an ongoing neurodegenerative process and with the development and extent of structural differences with increasing disease severity. The results correspond to earlier findings reported in individual studies and, importantly, overall correspond to the staging described by Braak,^32^ with some notable exceptions that fit alternative staging theories. The results of this study highlight the importance of adequately powered multicenter collaborations to reveal disease patterns.

## Author Roles

(1) Research Project: A. Conception, B. Organization, C. Execution; (2) Statistical Analysis: A. Design, B. Execution, C. Review and Critique; (3) Manuscript: A. Writing of the first draft, B. Review and Critique.

Y.D.v.d.W.: 1A, 1B, 2A, 2C, 3B

M.A.L.: 1A, 1B, 1C, 2A, 2B, 3A

J.K.B.: 1A, 1B, 1C, 2A, 2B, 3A

S.A.: 1C, 2C, 3B

T.J.A.: 1C, 2C, 3B

T.A.: 2C, 3B

F.A.: 1C, 2C, 3B

K.A.B.: 1C, 2C, 3B

H.W.B.: 1C, 2C, 3B

J.B.: 1C, 2C, 3B

F.C.: 1C, 2C, 3B

J.C.D.: 1C, 2C, 3B

R.M.A.d.B.: 1C, 2C, 3B

I.D.: 1C, 2C, 3B

M.F.D.: 1C, 2C, 3B

J.D.: 1C, 2C, 3B

H.C.A.E.: 1C, 2C, 3B

G.G.: 1C, 2C, 3B

R.P.G.: 1C, 2C, 3B

B.A.G.: 1A, 1C, 2C, 3B

R.C.H.: 1C, 2C, 3B

J.C.K.: 1C, 2C, 3B

C.E.M.: 1C, 2C, 3B

C.T.M.: 1C, 2C, 3B

T.R.M.: 1C, 2C, 3B

L.M.P.: 1C, 2C, 3B

F.P.: 1C, 2C, 3B

T.L.P.: 1C, 2C, 3B

K.L.P.: 1C, 2C, 3B

M.R.: 1C, 2C, 3B

L.F.R.: 1C, 2C, 3B

C.S.R.: 1C, 2C, 3B

C.R.: 1C, 2C, 3B

L.S.R.S.: 1C, 2C, 3B

R.S.: 1C, 2C, 3B

P.S.: 1C, 2C, 3B

G.S.: 1C, 2C, 3B

L.S.: 1C, 2C, 3B

O.A.v.d.H.: 1C, 2C, 3B

C.V.: 1C, 2C, 3B

J.W.: 1C, 2C, 3B

D.W.: 1C, 2C, 3B

R.W.: 1C, 2C, 3B

C.L.Y.: 1C, 2C, 3B

N.J.: 1A, 2C, 3B

P.M.T.: 1A, 2C, 3B

## Financial Disclosures of All Authors (for the Preceding 12 Months)

Y.D.v.d.W.: none. M.A.L.: none. J.K.B.: none. S.A.: grant from the Dowager Countess Eleanor Peel Trust Medical Research Grant. T.J.A.: none. T.A.: grants from Alzheimer's Disease Neuroimaging Initiative (U01AG02490), Neurodegeneration in Aging Down Syndrome (U01AG051406). F.A.: none. K.A.B.: none. H.W.B.: grants from ZonMw, Michael J. Fox Foundation. J.B.: none. F.C.: Employment at National Council for Scientific and Technological Development (CNPq). J.C.D.: grants from New Zealand Neurological Foundation, Health Research Council of New Zealand, Brain Research New Zealand, School of Psychology, Speech and Hearing. R.M.A.d.B.: research support paid to the institution from Medtronic, Lysosomal Therapeutics, Neuroderm, ZonMw, Parkinson Vereniging, Stichting Parkinson Nederland. I.D.: grants from Boston Scientific, AbbVie, Medtronic, UCB, Bial. Reimbursement: Zambon, Boston Scientific. M.F.D.: none. J.D.: none. H.C.A.E.: grants from NIHR, EPSRC, MRC. G.G.: none. R.P.G.: Employment at National Council for Scientific and Technological Development (CNPq). B.A.G.: grant from Alzheimer's Association Grant 2018‐AARG‐592081. R.C.H.: grants from the Michael J. Fox Foundation (grants 16048 and 15581), Netherlands Organization for Scientific Research (VENI grant 91617077). J.C.K.: none. C.E.M.: none. C.T.M.: none. T.R.M.: none. L.M.P.: grants from Alzheimer's Society, EPSRC UK, EU. F.P.: grants from Italian Ministry of Health RC 19, RC 20. T.L.P.: grant from New Zealand Brain Research Institute; honorarium from Stata Australia Ltd. K.L.P.: grants from the Michael J. Fox Foundation for Parkinson's Research, NIH; honoraria from invited scientific presentations to universities and professional societies not exceeding $5000/year; reimbursement from Sanofi, AstraZeneca, Sangamo BioSciences; consultancies from Allergan, Curasen. M.R.: none. L.F.R.: employment at National Council for Scientific and Technological Development (CNPq). C.S.R.: employment at National Council for Scientific and Technological Development (CNPq). C.R.: grants from Swiss National Science Foundation (projects CRSK‐3_190817/1 and CRSII5_180365), Novartis AG (FreeNovation research grant). L.S.R.S.: employment at National Council for Scientific and Technological Development (CNPq). R.S.: none. P.S.: honoraria from AbbVie, GmbH. G.S.: none. L.S.: none. O.A.v.d.H.: VIDI grant from The Netherlands Organization for Health Research (ZonMw; project number: 91717306); consultancy from Lundbeck. C.V.: none. J.W.: grants MOST 109‐2314‐B‐182‐021‐MY3, MOST 109‐2221‐E‐182‐009‐MY3, MOST 106‐2314‐B‐182‐018‐MY3. D.W.: grants from Michael J. Fox Foundation for Parkinson's Research, Alzheimer's Therapeutic Research Initiative (ATRI), Alzheimer's Disease Cooperative Study (ADCS), the International Parkinson and Movement Disorder Society (IPMDS), the National Institute on Aging (NIA); honoraria from Acadia, Aptinyx, CHDI Foundation, Clintrex LLC (Alkahest, Aptinyx, Avanir, Otsuka), Eisai, Enterin, Great Lake Neurotechnologies, Janssen, Sage, Scion, Signant Health, Sunovion, and Vanda. license fee payments from the University of Pennsylvania for the QUIP and QUIP‐RS. R.W.: grants from the Swiss National Science Foundation (projects CRSII5_180365, 320030L_170060), Swiss Personalized Health Network (driver project 2018DRI10), Swiss Innovation Council (project 43087.1 IP‐LS) University of Bern (sitem‐insel Support Funds 2019), Biogen (research project CHE‐TYS‐18‐11,316). C.L.Y.: employment at National Council for Scientific and Technological Development (CNPq). N.J.: grants from Biogen Inc. P.M.T.: NIH grants U01AG024904, R01MH111671.

## Supporting information


**Appendix S1.** Supplementary Information

## Data Availability

Open source datasets that support the findings of this study include PPMI (ppmi‐info.org), OpenNeuro Japan (openneuro.org/datasets/ds000245/), and Neurocon and Tao Wu's data set (fcon_1000.projects.nitrc.org/indi/retro/parkinsons.html). The other cohorts retain ownership of their scans and only share the anonymized outcome variables. Data are thus not openly available, but researchers are invited to register interest with the ENIGMA‐PD Working Group in order to formally request site data through secondary proposals. These proposals are then considered by the individual site's principal investigators.
