# Supplementary material for: International Multicenter Analysis of Brain Structure Across Clinical Stages of Parkinson's Disease
Source: Mov Disord. 2021 Jul 20;36(11):2583–94. doi: 10.1002/mds.28706 (PMC8595579; doi:10.1002/mds.28706)

Table of Contents

SUPPLEMENTARY TABLE 1A: SITE-SPECIFIC INCLUSION/EXCLUSION CRITERIA 3

SUPPLEMENTARY TABLE 1B: SITE-SPECIFIC MAGNETIC RESONANCE IMAGING (MRI) SCANNER INFORMATION 5

SUPPLEMENTARY TABLE 2A: CORTICAL THICKNESS ANALYSIS RESULTS – WHOLE SAMPLE PD VS CONTROLS 6

SUPPLEMENTARY TABLE 2B: CORTICAL SURFACE AREA ANALYSIS RESULTS – WHOLE SAMPLE PD VS CONTROLS 8

SUPPLEMENTARY TABLE 2C: SUBCORTICAL VOLUME ANALYSIS – WHOLE SAMPLE PD VS CONTROLS 9

SUPPLEMENTARY TABLE 3A: SUMMARY OF CORTICAL THICKNESS RESULTS IN ANALYSIS OF PD VS HEALTHY CONTROLS ACROSS HY STAGES 10

SUPPLEMENTARY TABLE 3B: SUMMARY OF SIGNIFICANT CORTICAL SURFACE AREA RESULTS IN ANALYSIS OF PD VS HEALTHY CONTROLS ACROSS HY STAGES 10

SUPPLEMENTARY TABLE 3C: SUMMARY OF SUBCORTICAL VOLUME RESULTS IN ANALYSIS OF PD VS HEALTHY CONTROLS ACROSS HY STAGES 11

SUPPLEMENTARY TABLE 4A: CORTICAL THICKNESS RESULTS - HY1 PD VS CONTROLS 11

SUPPLEMENTARY TABLE 4B: CORTICAL THICKNESS RESULTS – HY2 PD VS CONTROLS 13

SUPPLEMENTARY TABLE 4C: CORTICAL THICKNESS RESULTS – HY3 PD VS CONTROLS 14

SUPPLEMENTARY TABLE 4D: CORTICAL THICKNESS RESULTS - HY4&5 PD VS CONTROLS 16

SUPPLEMENTARY TABLE 4E: CORTICAL SURFACE AREA RESULTS - HY1 PD VS CONTROLS 18

SUPPLEMENTARY TABLE 4F: CORTICAL SURFACE AREA RESULTS - HY2 PD VS CONTROLS 19

SUPPLEMENTARY TABLE 4G: CORTICAL SURFACE AREA RESULTS - HY3 PD VS CONTROLS 21

SUPPLEMENTARY TABLE 4H: CORTICAL SURFACE AREA RESULTS - HY4&5 PD VS CONTROLS . 22

SUPPLEMENTARY TABLE 4I: SUBCORTICAL VOLUME RESULTS - HY1 PD VS CONTROLS 24

SUPPLEMENTARY TABLE 4J: SUBCORTICAL VOLUME RESULTS - HY2 PD VS CONTROLS 24

SUPPLEMENTARY TABLE 4K: SUBCORTICAL VOLUME RESULTS - HY3 PD VS CONTROLS 25

SUPPLEMENTARY TABLE 4L: SUBCORTICAL VOLUME RESULTS - HY4&5 PD VS CONTROLS 25

SUPPLEMENTARY TABLE 5A: RESULTS OF REGRESSION ANALYSIS OF MOCA SCORE AND CORTICAL THICKNESS 26

SUPPLEMENTARY TABLE 5B: RESULTS OF REGRESSION ANALYSIS OF MOCA SCORE AND SURFACE AREA 27

SUPPLEMENTARY TABLE 5C: RESULTS OF REGRESSION ANALYSIS OF MOCA SCORE AND SUBCORTICAL VOLUME 29

SUPPLEMENTARY TABLE 6A: CORTICAL THICKNESS RESULTS - PD (MOCA GROUP) VS CONTROLS 29

SUPPLEMENTARY TABLE 6B: CORTICAL SURFACE AREA RESULTS - PD (MOCA GROUP) VS CONTROLS 31

SUPPLEMENTARY TABLE 6C: PD (MOCA GROUP) VS CONTROLS – SUBCORTICAL VOLUMES RESULTS 32

SUPPLEMENTARY TABLE 7A: CORTICAL THICKNESS RESULTS - HY1 PD VS HY2 PD 33

SUPPLEMENTARY TABLE 7B: CORTICAL THICKNESS RESULTS - HY2 PD VS HY3PD 34

SUPPLEMENTARY TABLE 7C: CORTICAL THICKNESS RESULTS - HY3 PD VS HY4&5 PD 36

SUPPLEMENTARY TABLE 7D: CORTICAL SURFACE AREA RESULTS - HY1 PD VS HY2 PD 37
SUPPLEMENTARY TABLE 7E: CORTICAL SURFACE AREA RESULTS - HY2 PD VS HY3 PD 39
SUPPLEMENTARY TABLE 7F: CORTICAL SURFACE AREA RESULTS - HY3 PD VS HY4&5 PD 40
SUPPLEMENTARY TABLE 7G: SUBCORTICAL VOLUME RESULTS - HY1 PD VS HY2 PD 42
SUPPLEMENTARY TABLE 7H: SUBCORTICAL VOLUME RESULTS - HY2 PD VS HY3 PD 42
SUPPLEMENTARY TABLE 7I: SUBCORTICAL VOLUME RESULTS - HY3 PD VS HY4&5 PD 43
SUPPLEMENTARY TABLE 8A: HY STAGE 1 ANALYSIS SAMPLE CHARACTERISTICS 43
SUPPLEMENTARY TABLE 8B: HY STAGE 2 ANALYSIS SAMPLE CHARACTERISTICS 44
SUPPLEMENTARY TABLE 8C: HY STAGE 3 ANALYSIS SAMPLE CHARACTERISTICS 45
SUPPLEMENTARY TABLE 8D: HY STAGE 4&5 ANALYSIS SAMPLE CHARACTERISTICS 46
SUPPLEMENTARY TABLE 9: OVERLAP CONTROLS IN HY STAGE ANALYSES 47
SUPPLEMENTARY TABLE 10A: GROUP DIFFERENCES ON DURILL ACROSS HY STAGES 47
SUPPLEMENTARY TABLE 10B: GROUP DIFFERENCES ON MOCA ACROSS HY STAGES 48
SUPPLEMENTARY TABLE 11A: CORTICAL THICKNESS RESULTS - GROUP DIFFERENCES CORRECTED FOR DURILL ACROSS HY STAGES 48
SUPPLEMENTARY TABLE 11B: CORTICAL SURFACE AREA RESULTS - GROUP DIFFERENCES CORRECTED FOR DURILL ACROSS HY STAGES 50
SUPPLEMENTARY TABLE 11C: SUBCORTICAL VOLUME RESULTS - GROUP DIFFERENCES CORRECTED FOR DURILL ACROSS HY STAGES 51
SUPPLEMENTARY TABLE 12: MOCA SAMPLE CHARACTERISTICS 52
SUPPLEMENTARY TABLE 13: DURILL SAMPLE CHARACTERISTICS 52
SUPPLEMENTARY TABLE 14A: RESULTS OF REGRESSION ANALYSIS OF DURATION OF ILLNESS AND CORTICAL THICKNESS 53
SUPPLEMENTARY TABLE 14B: RESULTS OF REGRESSION ANALYSIS OF DURATION OF ILLNESS AND SURFACE AREA 55
SUPPLEMENTARY TABLE 14C: RESULTS OF REGRESSION ANALYSIS OF DURATION OF ILLNESS AND SUBCORTICAL VOLUME 56
SUPPLEMENTARY TABLE 14D: RESULTS OF REGRESSION ANALYSIS OF DURATION OF ILLNESS AND CORTICAL THICKNESS – UNCORRECTED FOR AGE 57
SUPPLEMENTARY TABLE 14E: RESULTS OF REGRESSION ANALYSIS OF DURATION OF ILLNESS AND CORTICAL SURFACE AREA – UNCORRECTED FOR AGE 58
SUPPLEMENTARY TABLE 14F: RESULTS OF REGRESSION ANALYSIS OF DURATION OF ILLNESS AND SUBCORTICAL VOLUME – UNCORRECTED FOR AGE 60
SUPPLEMENTARY TABLE 15A: CORTICAL THICKNESS RESULTS - MOCA REGRESSION ANALYSIS CORRECTED FOR DURILL 61
SUPPLEMENTARY TABLE 15B: CORTICAL SURFACE AREA RESULTS - MOCA REGRESSION ANALYSIS CORRECTED FOR DURILL 62
SUPPLEMENTARY TABLE 15C: SUBCORTICAL VOLUME RESULTS - MOCA REGRESSION ANALYSIS CORRECTED FOR DURILL 64
SUPPLEMENTARY FIGURE S1: WORLD MAP PARTICIPATING SITES 65

Supplementary Table 1a: Site-specific inclusion/exclusion criteria

Site	Cohort	Diagnostic criteria	Time between MRI and clinical assessment	Inclusion/Exclusion criteria PD	HC
Amsterdam	Amsterdam I	UKBB	Same day	**Inclusion:** consecutive patients seen at the movement disorders outpatient clinic. **Exclusion**: -	**Inclusion**: sex and age-matched **Exclusion**: -
	Amsterdam II (Cogtips)	UKBB	Same day	**Inclusion:** Subjective cognitive complaints (PD-CFRS > 3), HY stage < 4. **Exclusion:** dementia (SAGE <14 or MoCA < 22), drugs or alcohol abuse (CAGE AID > 1), depressive symptoms (BDI > 18), impulse control disorder (ICD criteria interview), psychotic symptoms (SAPS-PD criteria), tumors and significant vascular abnormalities.	No controls
Bern	BE I & II	UKBB	Within 7 days	**Inclusion:** PD and familial forms of typical Parkinsonian syndromes, motor complications of dopaminergic medication that are at least moderately bothersome to the patient. **Exclusion:** Age > 85 years, surgical or medical contraindications for a deep brain stimulation (DBS)-implantation, severe medical illness, severe personality disorder, dementia (DSM-V criteria and MMSE < 20, current psychosis, ongoing major depression (BDI-II > 23) or depression of any severity with suicidal ideation.	**Inclusion:** sex and age-matched **Exclusion**: -
Campinas	UNICAMP	UKBB	15.3 days on average (standard deviation=11.1)	**Inclusion**: idiopathic PD, taking antiparkinsonian medications, age > 30 years. **Exclusion:** clinically significant musculoskeletal, cardiovascular, respiratory or other neurological disease.	**Inclusion:** age > 30 years old. **Exclusion:** clinically significant musculoskeletal, cardiovascular, respiratory or neurological disease.
Chang Gung	CGU	NINDS	Within 30 days, except for one participant (45 days)	**Inclusion**: diagnosis of probable PD, ability to tolerate treatment discontinuation for 12 hours. **Exclusion:** major physical illnesses, psychiatric disorders, known brain abnormalities, history of intracranial surgery, pharmacotherapy for more than ten years or treatment with drugs able to cross the blood- brain-barrier (other than those used to treat PD).	**Inclusion:** aged between 50- 90. **Exclusion:** same as in PD.
Charlottesville	Charlottesville I-III	PD diagnosis confirmed by neurologist	73.9 days on average	**Inclusion:** PD diagnosis with a motor symptom that is not (or inconsistently) responsive to oral medication. **Exclusion:** -	No controls
Christchurch	Christchurch	UKBB	28 days on average (standard deviation=48)	**Inclusion:** met the UK Parkinson's Society criteria for PD, motor symptoms present for at least 1 year at study entry. **Exclusion:** atypical parkinsonian disorder, history of moderate/severe head injury, stroke, early-life learning disability, major psychiatric or medical illness in the previous 6 months, poor English (precluding testing).	**Inclusion**: - **Exclusion:** neurological disease/disorder; history of moderate/severe head injury, stroke, early-life learning disability, major psychiatric or medical illness in the previous 6 months, poor English (precluding testing).
Donders	Donders	UKBB	Same day	**Inclusion:** Idiopathic PD, UPDRS tremor-score > 2, dopaminergic therapy with a clear clinical response of non-tremor symptoms (bradykinesia, rigidity), HY stage 1-3. **Exclusion:** Neurological or psychiatric comorbidity, severe head tremor or dyskinesias, cognitive impairment (MMSE < 26), co-medication associated with elongated QT-time, pregnancy, age < 25 years.	**Inclusion:** same age/gender balance as PD patients **Exclusion**: Neurological or psychiatric disease, cognitive impairment (MMSE < 26), medication associated with elongated QT-time, pregnancy, age < 25 years.
Graz	PROMOVE/ ASPS I&II	QSBB	90% same day, maximum of 4 weeks	**Inclusion:** Clinical diagnosis of PD. **Exclusion:** MMSE <24, secondary parkinsonism, atypical parkinsonian diseases, a history of neuroleptic drugs,	**Inclusion:** No history of previous stroke or dementia and a normal neurologic examination. **Exclusion**: -

				structural abnormalities on routine MRI scans or a history of previous stroke.	
Liege	Liege I & II	UKBB	Same day	**Inclusion:** Non-demented PD patients.<br>**Exclusion**: -	**Inclusion:** age, sex, and highest achieved education level matched.<br>**Exclusion**: -
NW-England	NW-England I	UKBB	Same day	**Inclusion:** PD diagnosis without known clinical cardiovascular disease or dementia. No other significant neurological conditions.	**Inclusion:** age-matched to PD group and without a history of idiopathic PD or clinical CVD, or any other significant neurological condition.
	NW-England II	UKBB	Same day	**Inclusion:** as above.<br>**Exclusion**: -	**Inclusion:** age-matched to PD group and without a history of idiopathic PD or other significant neurological condition.
Milan	Milan	UKBB	Within 1 month	**Inclusion:** PD diagnosis.<br>**Exclusion**: -	**Inclusion:** -<br>**Exclusion**: -
NEUROCON	NEUROCON	MDS	Not available	**Inclusion:** Early- or moderate stage of PD.<br>**Exclusion**: -	**Inclusion:** no history of neurological or psychiatric disease.
ON Japan	ON Japan	UKBB	Not available	**Inclusion**: -<br>**Exclusion:** history of other neurological or psychiatric disease, focal white matter abnormalities. ACE-R score ≤ 88, psychiatric symptoms (hallucinations, depression etc)	**Inclusion**: -<br>**Exclusion:** neurological disease, family history of PD, or hyposmia, and with an ACE-R score > 88 in the study
Oxford	Oxford DISCOVERY	UKBB	108 days on average (standard deviation=104)	**Inclusion:** PD diagnosis within the past 3.5 years. Full details of criteria are available at: Szewczyk-Krolikowski K et. al. (2013). No atypical features to suggest an alternative diagnosis.<br>**Exclusion:** secondary parkinsonism due to head trauma or medication use, atypical parkinsonism syndromes (multiple system atrophy, progressive supra nuclear palsy, corticobasal degeneration, dementia with Lewy bodies), documented postural BP drop on standardized measurement or significant urinary symptoms.	**Inclusion:** controls without blood relatives with PD.
Pennsylvania	Pennsylvania	UKBB	MoCA: 53.7 days on average (standard deviation=67.1)<br><br>HY: 51.4 days on average (standard deviation=65.0)	**Inclusion:** clinical diagnosis of PD.<br>**Exclusion**: -	**Inclusion:** >40 years of age, MMSE > 27, a negative self-reported history of neurological or psychiatric condition, and MRI safe (e.g., no metal, claustrophobia).<br>**Exclusion**: -
PPMI	PPMI 1-21	MDS	Same day	**Inclusion & Exclusion** criteria detailed here: www.ppmi-info.org/wp-content/uploads/2013/02/PPMI-Protocol-AM5-Final-27Nov2012v6-2.pdf.	**Inclusion & Exclusion** criteria detailed here: www.ppmi-info.org/wp-content/uploads/2013/02/PPMI-Protocol-AM5-Final-27Nov2012v6-2.pdf.
Rome SLF	Rome SLF	MDS	1 day	**Inclusion:** diagnosis of idiopathic, MMSE score>26, no dementia.<br>**Exclusion:** presence of major non stabilized medical, known or suspected history of alcoholism, drug dependence and abuse, head trauma, and mental disorders (apart from mood or anxiety disorders, history of neurological diseases other than idiopathic PD, unclear history of chronic dopaminergic treatment responsiveness.	**Inclusion:** vision and hearing sufficient for compliance with testing procedures, laboratory values within normal reference intervals, neuropsychological domain scores above normal cognitive level cutoff scores, corrected for age and educational level.<br>**Exclusion:** dementia or MCI diagnosis, confirmed by a comprehensive neuropsychological battery,<br>MMSE score<26, presence of major non stabilized medical illnesses, known or suspected history of alcoholism, drug dependence and abuse, head trauma, and mental disorders (apart from mood or anxiety disorders).
Stanford	Stanford	UKBB	Within 3 months	**Inclusion:** > 20% improvement on MDS-UPDRS part III ON meds compared to OFF meds.<br>**Exclusion**: -	**Inclusion:** normal neurological exam and normal neuropsychiatric battery (within 1.5 SD of age- and education- adjusted norms).<br>**Exclusion**: -

Tao Wu	Tao Wu	MDS	1-2 days	**Inclusion:** diagnosis of PD based on medical history, physical and neurological examinations, response to levodopa or dopaminergic drugs, and laboratory tests and MRI scans to exclude other diseases. **Exclusion**: -	**Inclusion:** - **Exclusion**: -

Inclusion/exclusion criteria per sample for Parkinson's patients and healthy controls. PD, Parkinson's disease; MDS, Movement Disorders Society; NINDS, National Institute of Neurological Disorders and Stroke; UKBB, United Kingdom Parkinson's Disease Society Brain Bank; QSBB, Queen Square Brain Bank; HY, Hoehn & Yahr; MCI, mild cognitive impairment; MMSE, Mini Mental State Exam; MoCA, Montreal Cognitive Assessment; BDI, Beck's Depression Inventory; UPDRS, Unified Parkinson's Disease Rating Scale; PD-CFRS, Parkinson's Disease Cognitive Functional Rating Scale; SAGE, Self-administered Gerocognitive Examination; CAGE-AID, CAGE Adapted to Include Drugs.

Supplementary Table 1b: Site-specific magnetic resonance imaging (MRI) scanner information

Site	Cohort	Scanner Type	Field Strength	Protocol
Amsterdam	Amsterdam I	GE Discovery	3T	Sagittal 3-dimensional gradient-echo T1-weighted sequence (256 x 256 matrix; FOV = 25cm; voxel size = 1 x 0.98 x 0.98 mm; TR = 7.8 ms; TE = 3.0 ms; FA = 12°)
	Amsterdam II (Cogtips)	GE Discovery	3T	
Bern	BE I	Siemens Verio	3T	MDEFT sequence (1mm³ isotropic voxel; TR = 7.92ms, TE = 2.48ms, TI=910ms)
	BE II	Siemens Trio Tim	3T	As above
Campinas	UNICAMP	Philips Achieva	3T	3D T1 weighted image acquired on the sagittal plane (FOV of 240x240mm; 1mm³ isotropic voxel, TR = 7ms, TE = 3.2ms; FA = 8°)
Chang Gung	CGU	Siemens Magnetom Trio Tim	3T	T1-weighted images were acquired using an MPRAGE (224×256 matrix; FOV = 224 mm × 256 mm; 1mm³ isotropic voxel; TE = 2.63 ms; TR = 2000 ms, FA = 9°)
Charlottesville	PDNZ	Siemens	3T	Stock MPRAGE. Acquisition parameters vary by scanner protocol. Voxel size varied but did not exceed 1 × 1 × 1.2 mm.
Christchurch	Christchurch	General Electric HDx	3T	SPGR sequence
Donders	Donders	Siemens Trio	3T	
Graz	PROMOVE/ASPS I &II	3T Magnetom Trio/Prisma	3T	PD: structural T1-weighted MPRAGE sequence (1mm³ isotropic voxel; TR = 1900ms; TI = 900ms; FA = 9°; + TE: 2.19ms (101 patients) + TE: 2.7ms (23 patients) HC: structural T1-weighted MPRAGE sequence (1mm³ isotropic voxel; TR = 1900ms; TE = 2.19ms; TI = 900ms; FA = 9°)
Liege	Liege I	Siemens Magnetom Allegra	3T	3D multi-echo fast low angle shot (FLASH) sequence, (256 × 224 matrix; 1mm³ isotropic voxel; TR = 18.7 ms; TE = 2.2-14.7 ms; FA = 20°)
	Liege II	Siemens Magnetom Allegra	3T	As above
NW-England	NW-England I	Philips Achieva	3T	MPRAGE IR Method (voxel size 0.94 x 0.94 x 1 mm; FOV 240 (AP) x 192 (RL) mm TR=8.4ms, TE=3.9ms, TI=1150ms, FA = 8°)
	NW-England II	Philips Achieva	3T	As above
Milan	Milan	Philips Achieva	3T	240x240mm matrix; 1mm³ isotropic voxel; FOV = 33.7x24 cm; TR = 9.81ms; TE = 4.6ms; FA = 8°
NEUROCON	NEUROCON	Siemens Avanto	1.5T	MPRAGE IR Method. (voxel size 0.97 x 0.97 x 1mm; TR 1940ms TE 3.08ms)
ON Japan	ON Japan	Siemens Magnetom Verio	3T	High resolution T1-weighted images (256 × 256 matrix size; FOV = 256 mm; TR = 2.5 s, TE = 2.48 ms,)
Oxford	Oxford DISCOVERY	Siemens Trio	3T	MPRAGE (1mm³ isotropic voxel, TE = 4.7 ms; TR = 2040 ms; TI ¼ = 900ms; FA: 8°).
Pennsylvania	Pennsylvania	Siemens Trio/Prisma	3T	3D MPRAGE Sagittal & Axial (Slice thickness = 1mm; TR = 1620/1800/2300ms; TE = 2.95/3.8/3.09ms; TI = 900/950ms)

PPMI	PPMI 1-21	Siemens Trio Tim	3T	T1-3D e.g. MPRAGE, SPGR, Sagittal (56 x 256 x 170-200 matrix; Slice thickness = 1.2mm; voxel size 1x1x1.2mm)
Rome SLF	Rome SLF	Siemens Allegra	3T	T1 MDEFT (256x224 matrix; 1mm³ isotropic voxel; TR = 7.92ms; TE = 2.4ms; FA = 15°)
Stanford	Stanford	General Electric SIGNA	3T	FSPGR 3D T1 scan
Tao Wu	Tao Wu	Siemens Magnetom Trio	3T	MPRAGE IR method (1mm³ isotropic voxel; TR 1100ms; TE 3.39ms)

Scanner protocol information for each sample included in analyses. TR = Repetition Time; TE = Echo time; TI = Inversion time; FOV = field of view; FA = flip angle

Supplementary table 2a: Cortical thickness analysis results – whole sample PD vs controls

ROI Thickness	*p*	*d*	*b*	SE	Lower CI	Upper CI	% diff	NPD	NHC
**L Banks STS**	**<0.001**	**-0.104**	**-0.026**	**0.007**	**-0.040**	**-0.012**	**-1.105**	**2061**	**1081**
**R Banks STS**	**<0.001**	**-0.122**	**-0.031**	**0.007**	**-0.045**	**-0.017**	**-1.261**	**2208**	**1129**
L Caudal anterior cingulate	0.173	0.043	0.015	0.011	-0.006	0.036	0.567	2297	1169
R Caudal anterior cingulate	0.384	-0.027	-0.009	0.010	-0.028	0.011	-0.345	2295	1173
**L Caudal middle frontal**	**<0.001**	**-0.135**	**-0.027**	**0.006**	**-0.039**	**-0.015**	**-1.119**	**2300**	**1167**
R Caudal middle frontal	0.197	-0.040	-0.008	0.006	-0.020	0.004	-0.335	2300	1167
**L Cuneus**	**<0.001**	**-0.112**	**-0.021**	**0.006**	**-0.032**	**-0.010**	**-1.157**	**2142**	**1118**
**R Cuneus**	**<0.001**	**-0.108**	**-0.022**	**0.006**	**-0.033**	**-0.011**	**-1.225**	**2110**	**1085**
L Entorhinal	0.002	-0.096	-0.040	0.013	-0.066	-0.014	-1.240	2260	1163
**R Entorhinal**	**<0.001**	**-0.100**	**-0.051**	**0.014**	**-0.079**	**-0.024**	**-1.510**	**2201**	**1145**
L Frontal pole	0.747	-0.011	-0.003	0.011	-0.024	0.017	-0.128	2309	1169
R Frontal pole	0.003	-0.094	-0.030	0.010	-0.051	-0.010	-1.154	2306	1171
**L Fusiform**	**<0.001**	**-0.198**	**-0.043**	**0.006**	**-0.055**	**-0.030**	**-1.638**	**2274**	**1170**
**R Fusiform**	**<0.001**	**-0.117**	**-0.033**	**0.007**	**-0.047**	**-0.019**	**-1.260**	**2288**	**1172**
**L Inferior parietal**	**<0.001**	**-0.188**	**-0.036**	**0.006**	**-0.047**	**-0.025**	**-1.549**	**2213**	**1128**
**R Inferior parietal**	**<0.001**	**-0.194**	**-0.040**	**0.006**	**-0.051**	**-0.029**	**-1.694**	**2225**	**1153**
**L Inferior temporal**	**<0.001**	**-0.193**	**-0.042**	**0.007**	**-0.055**	**-0.029**	**-1.578**	**2211**	**1110**
**R Inferior temporal**	**<0.001**	**-0.141**	**-0.033**	**0.007**	**-0.046**	**-0.020**	**-1.231**	**2237**	**1129**
L Insula	0.269	-0.036	-0.008	0.007	-0.022	0.006	-0.273	2303	1168
R Insula	0.008	-0.077	-0.020	0.008	-0.036	-0.005	-0.710	2294	1163
**L Isthmus cingulate**	**<0.001**	**-0.177**	**-0.042**	**0.008**	**-0.057**	**-0.027**	**-1.792**	**2302**	**1173**
**R Isthmus cingulate**	**<0.001**	**-0.178**	**-0.044**	**0.008**	**-0.059**	**-0.029**	**-1.869**	**2297**	**1175**
**L Lateral occipital**	**<0.001**	**-0.166**	**-0.030**	**0.006**	**-0.042**	**-0.019**	**-1.440**	**2227**	**1151**
**R Lateral occipital**	**<0.001**	**-0.167**	**-0.033**	**0.006**	**-0.045**	**-0.021**	**-1.523**	**2246**	**1157**

**L Lateral orbitofrontal**	**<0.001**	**-0.125**	**-0.028**	**0.006**	**-0.040**	**-0.015**	**-1.089**	**2305**	**1168**
**R Lateral orbitofrontal**	**<0.001**	**-0.141**	**-0.034**	**0.006**	**-0.046**	**-0.021**	**-1.354**	**2304**	**1169**
**L Lingual**	**<0.001**	**-0.111**	**-0.019**	**0.005**	**-0.029**	**-0.009**	**-0.992**	**2244**	**1158**
**R Lingual**	**<0.001**	**-0.094**	**-0.018**	**0.005**	**-0.029**	**-0.008**	**-0.927**	**2203**	**1126**
L Medial orbitofrontal	0.001	-0.098	-0.020	0.006	-0.033	-0.008	-0.861	2286	1166
R Medial orbitofrontal	0.015	-0.067	-0.016	0.007	-0.029	-0.003	-0.696	2281	1165
**L Middle temporal**	**<0.001**	**-0.156**	**-0.036**	**0.007**	**-0.049**	**-0.022**	**-1.317**	**2101**	**1096**
**R Middle temporal**	**<0.001**	**-0.134**	**-0.033**	**0.007**	**-0.046**	**-0.020**	**-1.197**	**2224**	**1136**
**L Paracentral**	**<0.001**	**-0.109**	**-0.025**	**0.007**	**-0.038**	**-0.012**	**-1.116**	**2294**	**1170**
R Paracentral	0.076	-0.052	-0.012	0.007	-0.026	0.001	-0.532	2281	1173
**L Parahippocampal**	**<0.001**	**-0.122**	**-0.045**	**0.012**	**-0.068**	**-0.021**	**-1.708**	**2308**	**1169**
**R Parahippocampal**	**<0.001**	**-0.108**	**-0.035**	**0.010**	**-0.055**	**-0.016**	**-1.371**	**2296**	**1173**
L Pars opercularis	0.477	-0.020	-0.004	0.006	-0.016	0.007	-0.171	2300	1174
R Pars opercularis	0.364	-0.026	-0.006	0.006	-0.018	0.006	-0.230	2297	1172
**L Pars orbitalis**	**<0.001**	**-0.110**	**-0.028**	**0.008**	**-0.043**	**-0.013**	**-1.077**	**2297**	**1171**
R Pars orbitalis	0.004	-0.086	-0.022	0.008	-0.037	-0.007	-0.863	2296	1172
L Pars triangularis	0.077	-0.051	-0.010	0.006	-0.022	0.001	-0.453	2292	1174
R Pars triangularis	0.353	-0.027	-0.005	0.006	-0.017	0.006	-0.235	2284	1173
L Pericalcarine	0.052	-0.055	-0.011	0.006	-0.022	<0.001	-0.682	2149	1124
R Pericalcarine	0.026	-0.058	-0.013	0.006	-0.024	-0.002	-0.814	2077	1070
**L Postcentral**	**<0.001**	**-0.110**	**-0.019**	**0.005**	**-0.029**	**-0.009**	**-0.971**	**2175**	**1130**
**R Postcentral**	**<0.001**	**-0.104**	**-0.020**	**0.005**	**-0.030**	**-0.010**	**-0.998**	**2174**	**1134**
L Posterior cingulate	0.007	-0.082	-0.018	0.007	-0.030	-0.005	-0.736	2309	1173
**R Posterior cingulate**	**<0.001**	**-0.156**	**-0.036**	**0.006**	**-0.048**	**-0.023**	**-1.503**	**2308**	**1174**
**L Precentral**	**<0.001**	**-0.104**	**-0.023**	**0.006**	**-0.035**	**-0.010**	**-0.944**	**2205**	**1126**
R Precentral	0.086	-0.052	-0.011	0.007	-0.025	0.002	-0.485	2220	1131
**L Precuneus**	**<0.001**	**-0.156**	**-0.031**	**0.005**	**-0.042**	**-0.020**	**-1.373**	**2301**	**1173**
**R Precuneus**	**<0.001**	**-0.193**	**-0.038**	**0.006**	**-0.049**	**-0.027**	**-1.679**	**2304**	**1174**
L Rostral anterior cingulate	0.830	0.007	0.002	0.010	-0.017	0.021	0.075	2285	1164
R Rostral anterior cingulate	0.732	-0.010	-0.003	0.010	-0.022	0.015	-0.121	2282	1164
L Rostral middle frontal	0.001	-0.088	-0.016	0.005	-0.026	-0.007	-0.728	2286	1170
R Rostral middle frontal	0.063	-0.047	-0.010	0.005	-0.021	0.001	-0.452	2299	1168
**L Superior frontal**	**<0.001**	**-0.104**	**-0.020**	**0.006**	**-0.031**	**-0.009**	**-0.784**	**2257**	**1163**
**R Superior frontal**	**<0.001**	**-0.110**	**-0.022**	**0.006**	**-0.033**	**-0.011**	**-0.879**	**2258**	**1163**
**L Superior parietal**	**<0.001**	**-0.145**	**-0.028**	**0.005**	**-0.038**	**-0.017**	**-1.335**	**2233**	**1150**
**R Superior parietal**	**<0.001**	**-0.174**	**-0.036**	**0.005**	**-0.046**	**-0.025**	**-1.709**	**2254**	**1161**
L Superior temporal	0.001	-0.096	-0.021	0.007	-0.034	-0.008	-0.819	2019	1070
R Superior temporal	0.002	-0.077	-0.019	0.006	-0.032	-0.007	-0.739	2147	1114
**L Supramarginal**	**<0.001**	**-0.131**	**-0.026**	**0.006**	**-0.037**	**-0.014**	**-1.080**	**2182**	**1118**
**R Supramarginal**	**<0.001**	**-0.153**	**-0.031**	**0.006**	**-0.042**	**-0.019**	**-1.280**	**2209**	**1148**
L Temporal pole	0.002	-0.102	-0.040	0.013	-0.066	-0.015	-1.132	2215	1150
R Temporal pole	0.001	-0.096	-0.043	0.013	-0.069	-0.017	-1.174	2219	1142
L Transverse temporal	0.023	-0.072	-0.019	0.009	-0.036	-0.003	-0.884	2319	1174
R Transverse temporal	0.006	-0.068	-0.025	0.009	-0.043	-0.007	-1.110	2317	1175

Results of cortical thickness analysis in the whole sample of PD vs healthy controls. L – Left, R – Right, ROI – region of interest, Banks STS – Banks of the superior temporal sulcus. **Boldface** denotes significant results after Bonferroni correction

Supplementary table 2b: Cortical surface area analysis results – whole sample PD vs controls

ROI Surface Area	*p*	*d*	*b*	SE	Lower CI	Upper CI	% diff	N_{PD}	N_{HC}
L Banks STS	0.923	-0.003	-0.56	5.76	-11.86	10.75	-0.056	2059	1069
R Banks STS	0.280	0.036	5.25	4.86	-4.29	14.79	0.577	2209	1131
L Caudal anterior cingulate	0.138	0.051	7.21	4.85	-2.31	16.72	1.156	2296	1168
R Caudal anterior cingulate	0.566	0.019	3.19	5.56	-7.71	14.08	0.425	2291	1172
L Caudal middle frontal	0.261	0.039	13.40	11.92	-9.98	36.78	0.605	2297	1165
R Caudal middle frontal	0.960	-0.002	-0.62	12.13	-24.40	23.17	-0.030	2301	1166
L Cuneus	0.002	-0.112	-23.49	7.50	-38.19	-8.79	-1.620	2142	1110
R Cuneus	0.002	-0.113	-23.57	7.55	-38.38	-8.76	-1.572	2106	1063
L Entorhinal	0.446	-0.026	-2.42	3.18	-8.65	3.81	-0.586	2260	1162
R Entorhinal	0.146	-0.051	-4.25	2.92	-9.97	1.48	-1.187	2204	1143
**L Frontal pole**	**<0.001**	**-0.167**	**-6.53**	**1.20**	**-8.89**	**-4.17**	**-3.081**	**2307**	**1169**
R Frontal pole	0.185	-0.043	-2.04	1.54	-5.05	0.98	-0.734	2308	1171
L Fusiform	0.231	-0.040	-15.47	12.92	-40.80	9.86	-0.497	2274	1170
R Fusiform	0.198	-0.043	-16.44	12.78	-41.49	8.61	-0.540	2288	1171
L Inferior parietal	0.388	-0.029	-17.28	19.99	-56.47	21.92	-0.389	2214	1127
R Inferior parietal	0.794	0.009	5.98	22.87	-38.86	50.82	0.114	2229	1153
L Inferior temporal	0.084	-0.059	-26.98	15.61	-57.58	3.62	-0.840	2214	1107
R Inferior temporal	0.151	-0.049	-20.44	14.22	-48.32	7.44	-0.669	2239	1128
L Insula	0.570	0.018	4.54	7.98	-11.11	20.19	0.206	2302	1160
R Insula	0.986	0.001	0.17	9.26	-17.98	18.31	0.007	2294	1156
L Isthmus cingulate	0.007	0.093	15.71	5.79	4.36	27.07	1.572	2302	1171
R Isthmus cingulate	0.003	0.100	15.66	5.22	5.42	25.90	1.696	2296	1173
**L Lateral occipital**	**<0.001**	**-0.118**	**-69.95**	**20.05**	**-109.27**	**-30.63**	**-1.477**	**2229**	**1151**
R Lateral occipital	0.006	-0.093	-56.27	20.62	-96.70	-15.85	-1.220	2247	1155
L Lateral orbitofrontal	0.413	-0.028	-7.65	9.35	-25.98	10.68	-0.302	2305	1168
R Lateral orbitofrontal	0.113	0.053	16.13	10.18	-3.84	36.10	0.652	2302	1170
L Lingual	0.036	-0.072	-30.93	14.71	-59.77	-2.09	-1.038	2241	1155
R Lingual	<0.001	-0.114	-48.71	14.60	-77.33	-20.08	-1.609	2204	1118
L Medial orbitofrontal	0.646	-0.016	-3.52	7.67	-18.55	11.51	-0.194	2285	1160
R Medial orbitofrontal	0.219	-0.041	-8.27	6.72	-21.44	4.90	-0.459	2281	1160
L Middle temporal	0.009	-0.092	-35.19	13.42	-61.49	-8.88	-1.178	2101	1087
R Middle temporal	0.022	-0.077	-30.35	13.28	-56.39	-4.30	-0.922	2224	1136
L Paracentral	0.003	0.103	19.14	6.42	6.56	31.73	1.441	2291	1169
R Paracentral	0.005	0.097	21.13	7.45	6.53	35.73	1.403	2281	1172
L Parahippocampal	0.338	0.032	3.49	3.64	-3.65	10.62	0.515	2307	1170
R Parahippocampal	0.024	0.076	7.53	3.33	1.00	14.06	1.154	2293	1173
L Pars opercularis	0.684	-0.014	-3.52	8.65	-20.47	13.43	-0.222	2303	1172
R Pars opercularis	0.926	-0.003	-0.69	7.45	-15.29	13.90	-0.052	2298	1168
L Pars orbitalis	0.016	-0.081	-6.71	2.77	-12.15	-1.27	-1.080	2295	1171
R Pars orbitalis	0.802	0.009	0.87	3.49	-5.96	7.71	0.116	2299	1172
L Pars triangularis	0.764	-0.011	-1.99	6.62	-14.97	10.99	-0.161	2292	1173

R Pars triangularis	0.737	0.012	2.76	8.22	-13.36	18.88	0.194	2285	1170
L Pericalcarine	0.037	-0.074	-18.74	8.98	-36.35	-1.13	-1.378	2147	1113
R Pericalcarine	0.094	-0.060	-15.76	9.42	-34.22	2.70	-1.065	2076	1044
L Postcentral	0.293	0.036	15.95	15.16	-13.78	45.68	0.390	2176	1132
R Postcentral	0.939	-0.003	-1.15	15.14	-30.85	28.54	-0.029	2172	1136
L Posterior cingulate	0.102	0.056	11.36	6.95	-2.27	25.00	1.005	2309	1169
R Posterior cingulate	0.090	0.056	10.32	6.07	-1.59	22.23	0.893	2306	1172
L Precentral	0.277	0.038	18.29	16.81	-14.67	51.24	0.380	2204	1126
R Precentral	0.027	0.076	37.80	17.04	4.39	71.22	0.784	2221	1134
L Precuneus	0.305	-0.035	-14.98	14.61	-43.63	13.67	-0.407	2304	1171
R Precuneus	0.687	0.014	6.21	15.43	-24.04	36.45	0.162	2304	1172
L Rostral anterior cingulate	0.661	0.015	2.42	5.53	-8.42	13.27	0.296	2280	1141
R Rostral anterior cingulate	0.944	-0.002	-0.33	4.76	-9.66	9.00	-0.050	2284	1157
L Rostral middle frontal	0.225	-0.040	-27.57	22.70	-72.08	16.95	-0.506	2286	1171
R Rostral middle frontal	0.092	-0.056	-40.04	23.75	-86.60	6.52	-0.709	2302	1168
L Superior frontal	0.411	-0.028	-19.93	24.22	-67.42	27.56	-0.286	2259	1149
R Superior frontal	0.745	0.011	7.90	24.29	-39.72	55.52	0.118	2261	1161
L Superior parietal	0.035	-0.073	-44.16	20.97	-85.27	-3.06	-0.835	2233	1150
R Superior parietal	0.673	-0.014	-8.64	20.47	-48.78	31.49	-0.164	2252	1160
L Superior temporal	0.617	-0.018	-7.18	14.34	-35.30	20.94	-0.195	2019	1062
R Superior temporal	0.862	0.006	2.14	12.27	-21.92	26.21	0.061	2148	1113
L Supramarginal	0.884	0.005	2.78	19.03	-34.53	40.09	0.073	2181	1116
R Supramarginal	0.715	0.013	6.17	16.91	-27.00	39.33	0.172	2215	1149
L Temporal pole	0.141	-0.050	-3.26	2.21	-7.60	1.08	-0.691	2217	1150
R Temporal pole	0.301	-0.035	-2.40	2.32	-6.95	2.15	-0.564	2219	1141
L Transverse temporal	0.010	0.088	6.92	2.67	1.68	12.16	1.549	2318	1174
R Transverse temporal	0.259	0.039	2.16	1.91	-1.59	5.91	0.649	2318	1176

Results of cortical surface area analysis in the whole sample of PD vs healthy controls. L – Left, R – Right, ROI – region of interest, Banks STS – Banks of the superior temporal sulcus. **Boldface** denotes significant results after Bonferroni correction

Supplementary table 2c: Subcortical volume analysis – whole sample PD vs controls

ROI Subcortical	*p*	*d*	*b*	SE	Lower CI	Upper CI	% diff	NPD	NHC
**L Amygdala**	**<0.001**	**-0.130**	**-33.64**	**7.99**	**-49.3**	**-17.97**	**-2.267**	**2284**	**1158**
R Amygdala	0.011	-0.077	-21.25	8.35	-37.63	-4.86	-1.338	2279	1152
L Caudate nucleus	0.028	-0.071	-37.44	16.99	-70.75	-4.13	-1.083	2312	1162
R Caudate nucleus	0.295	-0.033	-17.78	16.99	-51.08	15.52	-0.506	2319	1173
L Globus pallidus	0.026	-0.066	-21.63	9.74	-40.73	-2.52	-1.420	2177	1063
R Globus pallidus	0.069	-0.053	-15.82	8.69	-32.85	1.21	-1.032	2248	1143
L Hippocampus	0.529	-0.020	-10.54	16.75	-43.38	22.29	-0.268	2265	1141
R Hippocampus	0.314	-0.031	-17.25	17.13	-50.84	16.34	-0.425	2302	1151
L Lateral ventricle	0.174	0.047	343.41	252.63	-151.91	838.74	2.613	2342	1169
R Lateral ventricle	0.028	0.077	511.96	233.14	54.86	969.05	4.243	2343	1170
L Nucleus accumbens	0.363	-0.028	-3.65	4.02	-11.53	4.22	-0.768	2302	1158
R Nucleus accumbens	0.098	-0.052	-6.39	3.86	-13.95	1.18	-1.283	2264	1147

**L Putamen**	**<0.001**	**-0.136**	**-101.89**	**24.2**	**-149.34**	**-54.44**	**-2.031**	**2163**	**1068**
**R Putamen**	**<0.001**	**-0.135**	**-96.94**	**22.76**	**-141.56**	**-52.31**	**-2.014**	**2230**	**1125**
**L Thalamus**	**<0.001**	**0.131**	**127.97**	**28.29**	**72.5**	**183.44**	**1.785**	**2193**	**1125**
R Thalamus	0.009	0.078	61.44	23.45	15.46	107.42	0.918	2263	1139

Results of subcortical analysis in the whole sample of PD vs healthy controls. L – Left, R – Right, ROI – region of interest. **Boldface** denotes significant results after Bonferroni correction

Supplementary table 3a: Summary of cortical thickness results in analysis of PD vs healthy controls across HY stages

HY1 N_{PD} 437 N_{HC} 847		HY2 N_{PD} 941 N_{HC} 908		HY3 N_{PD} 263 N_{HC} 502		HY4&5 N_{PD} 85 N_{HC} 329	
Significant ROIs ($P<7.35x10^{-4}$)	Cohen's *d*	Significant ROIs ($P<7.35x10^{-4}$)	Cohen's *d*	Significant ROIs ($P<7.35x10^{-4}$)	Cohen's *d*	Significant ROIs ($P<7.35x10^{-4}$)	Cohen's *d*
L Fusiform	-0.160	L Fusiform	-0.173	L Caudal middle frontal	-0.258	L Banks STS	-0.473
L Inferior temporal	-0.182	L Inferior parietal	-0.175	L Entorhinal	-0.276	L Caudal middle frontal	-0.379
R Inferior parietal	-0.217	L Inferior temporal	-0.141	L Fusiform	-0.372	L Entorhinal	-0.399
R Precuneus	-0.168	L Isthmus cingulate	-0.160	L Inferior parietal	-0.359	L Fusiform	-0.583
R Superior parietal	-0.170	L Parahippocampal	-0.173	L Inferior temporal	-0.336	L Inferior parietal	-0.522
		L Precuneus	-0.150	L Isthmus cingulate	-0.283	L Inferior temporal	-0.474
		L Superior parietal	-0.134	L Lateral occipital	-0.220	L Isthmus cingulate	-0.427
		L Temporal pole	-0.158	L Lingual	-0.217	L Lateral orbitofrontal	-0.402
		R Fusiform	-0.132	L Middle temporal	-0.311	L Middle temporal	-0.484
		R Inferior parietal	-0.172	L Paracentral	-0.211	L Postcentral	-0.343
		R Isthmus cingulate	-0.173	L Precentral	-0.244	L Precuneus	-0.421
		R Lateral occipital	-0.136	L Precuneus	-0.267	L Superior temporal	-0.467
		R Precuneus	-0.183	L Superior parietal	-0.226	L Supramarginal	-0.495
		R Superior parietal	-0.151	L Superior temporal	-0.304	L Transverse temporal	-0.367
		R Supramarginal	-0.146	L Supramarginal	-0.265	R Entorhinal	-0.370
				R Banks STS	-0.249	R Fusiform	-0.407
				R Entorhinal	-0.280	R Inferior parietal	-0.517
				R Fusiform	-0.204	R Inferior temporal	-0.466
				R Inferior parietal	-0.321	R Insula	-0.393
				R Inferior temporal	-0.273	R Isthmus cingulate	-0.366
				R Isthmus cingulate	-0.237	R Lateral orbitofrontal	-0.484
				R Lateral occipital	-0.239	R Medial orbitofrontal	-0.366
				R Middle temporal	-0.230	R Middle temporal	-0.461
				R Parahippocampal	-0.298	R Posterior cingulate	-0.463
				R Posterior cingulate	-0.256	R Precuneus	-0.449
				R Precuneus	-0.326	R Superior parietal	-0.312
				R Superior frontal	-0.220	R Superior temporal	-0.368
				R Superior parietal	-0.261	R Supramarginal	-0.521
				R Superior temporal	-0.191	R Temporal pole	-0.428
				R Supramarginal	-0.300		

Supplementary table 3b: Summary of significant cortical surface area results in analysis of PD vs healthy controls across HY stages

HY1 N_{PD} 437	HY2 N_{PD} 941	HY3 N_{PD} 263	HY4&5 N_{PD} 85

N_{HC} 847		N_{HC} 908		N_{HC} 502		N_{HC} 329	
Significant ROIs ($P<7.35\times10^{-4}$)	Cohen's *d*	Significant ROIs ($P<7.35\times10^{-4}$)	Cohen's *d*	Significant ROIs ($P<7.35\times10^{-4}$)	Cohen's *d*	Significant ROIs ($P<7.35\times10^{-4}$)	Cohen's *d*
L Frontal Pole	-0.219	L Lateral occipital	-0.148	L Frontal Pole	-0.291	L Inferior temporal	-0.405
		L Lingual	-0.154	L Fusiform	-0.260	L Lateral occipital	-0.423
		R Lingual	-0.175	L Inferior temporal	-0.260	L Precuneus	-0.424
		R Pericalcarine	-0.168	L Lateral occipital	-0.324	R Precuneus	-0.400
				L Lingual	-0.253		
				L Middle temporal	-0.300		
				L Precuneus	-0.284		
				L Rostral middle frontal	-0.256		
				L Superior parietal	-0.306		
				R Cuneus	-0.271		
				R Inferior temporal	-0.270		
				R Lateral occipital	-0.286		
				R Lingual	-0.294		
				R Middle temporal	-0.353		
				R Rostral middle frontal	-0.248		
				R Superior parietal	-0.257		

Supplementary table 3c: Summary of subcortical volume results in analysis of PD vs healthy controls across HY stages

HY1 N_{PD} 437 N_{HC} 847		HY2 N_{PD} 941 N_{HC} 908		HY3 N_{PD} 263 N_{HC} 502		HY4&5 N_{PD} 85 N_{HC} 329	
Significant ROIs ($P<6.25\times10^{-5}$)	Cohen's *d*	Significant ROIs ($P<6.25\times10^{-5}$)	Cohen's *d*	Significant ROIs ($P<6.25\times10^{-5}$)	Cohen's *d*	Significant ROIs ($P<6.25\times10^{-5}$)	Cohen's *d*
L Thalamus	0.151	L Amygdala	-0.159	L Amygdala	-0.441	L Amygdala	-0.640
		L Putamen	-0.152	L Caudate nucleus	-0.221	L Caudate nucleus	-0.330
		R Amygdala	-0.119	L Hippocampus	-0.240	L Globus pallidus	-0.317
		R Putamen	-0.152	L Putamen	-0.267	L Hippocampus	-0.546
				R Amygdala	-0.316	L Lateral ventricle	-0.357
				R Hippocampus	-0.283	L Putamen	-0.423
				R Putamen	-0.239	R Amygdala	-0.554
						R Hippocampus	-0.616
						R Lateral ventricle	0.523
						R Nucleus accumbens	-0.473
						R Putamen	-0.473

Supplementary Table 4a: Cortical thickness results - HY1 PD vs controls

ROI Thickness	*p*	*d*	*b*	SE	Lower CI	Upper CI	% diff	NPD	NHC
L Banks STS	0.156	-0.071	-0.017	0.012	-0.041	0.007	-0.746	383	784
R Banks STS	0.013	-0.118	-0.030	0.012	-0.053	-0.006	-1.233	418	810
L Caudal anterior cingulate	0.594	-0.028	-0.009	0.017	-0.043	0.025	-0.356	433	840
R Caudal anterior cingulate	0.638	-0.024	-0.007	0.015	-0.036	0.022	-0.284	431	843
L Caudal middle frontal	0.002	-0.160	-0.031	0.010	-0.050	-0.011	-1.284	430	840
R Caudal middle frontal	0.302	-0.054	-0.011	0.010	-0.031	0.010	-0.447	430	840

L Cuneus	0.061	-0.089	-0.016	0.009	-0.033	0.001	-0.913	404	807
R Cuneus	0.005	-0.121	-0.025	0.009	-0.043	-0.008	-1.403	400	785
L Entorhinal	0.516	-0.034	-0.014	0.021	-0.056	0.028	-0.426	425	837
R Entorhinal	0.415	-0.035	-0.018	0.022	-0.061	0.025	-0.526	418	825
L Frontal pole	0.623	0.026	0.008	0.017	-0.025	0.042	0.318	429	841
R Frontal pole	0.389	-0.046	-0.014	0.017	-0.047	0.018	-0.553	430	843
**L Fusiform**	**<0.001**	**-0.160**	**-0.034**	**0.010**	**-0.053**	**-0.015**	**-1.313**	**426**	**841**
R Fusiform	0.020	-0.097	-0.028	0.012	-0.051	-0.004	-1.053	430	843
L Inferior parietal	0.003	-0.147	-0.028	0.009	-0.046	-0.010	-1.217	426	815
**R Inferior parietal**	**<0.001**	**-0.217**	**-0.045**	**0.009**	**-0.063**	**-0.026**	**-1.906**	**421**	**830**
**L Inferior temporal**	**<0.001**	**-0.182**	**-0.038**	**0.010**	**-0.058**	**-0.018**	**-1.433**	**409**	**804**
R Inferior temporal	0.003	-0.131	-0.030	0.010	-0.050	-0.010	-1.121	423	812
L Insula	0.987	0.001	<0.001	0.011	-0.022	0.022	0.006	430	840
R Insula	0.271	-0.052	-0.013	0.012	-0.037	0.010	-0.459	429	835
L Isthmus cingulate	0.008	-0.140	-0.034	0.013	-0.060	-0.009	-1.451	433	843
R Isthmus cingulate	0.002	-0.151	-0.038	0.012	-0.062	-0.013	-1.608	432	846
L Lateral occipital	0.002	-0.157	-0.028	0.009	-0.045	-0.010	-1.314	420	827
**R Lateral occipital**	**<0.001**	**-0.165**	**-0.033**	**0.010**	**-0.052**	**-0.014**	**-1.525**	**426**	**832**
L Lateral orbitofrontal	0.003	-0.140	-0.029	0.010	-0.048	-0.010	-1.152	426	840
R Lateral orbitofrontal	0.031	-0.094	-0.021	0.010	-0.041	-0.002	-0.866	428	841
L Lingual	0.027	-0.105	-0.018	0.008	-0.034	-0.002	-0.938	426	829
R Lingual	0.159	-0.060	-0.012	0.008	-0.029	0.005	-0.606	417	807
L Medial orbitofrontal	0.016	-0.122	-0.025	0.010	-0.045	-0.005	-1.066	425	836
R Medial orbitofrontal	0.327	-0.045	-0.010	0.011	-0.031	0.010	-0.453	425	837
L Middle temporal	0.020	-0.115	-0.026	0.011	-0.048	-0.004	-0.971	386	787
R Middle temporal	0.006	-0.120	-0.029	0.011	-0.050	-0.008	-1.067	417	812
L Paracentral	0.008	-0.125	-0.028	0.011	-0.049	-0.007	-1.251	432	841
R Paracentral	0.076	-0.084	-0.020	0.011	-0.042	0.002	-0.863	431	844
L Parahippocampal	0.027	-0.119	-0.042	0.019	-0.079	-0.005	-1.604	432	841
R Parahippocampal	0.007	-0.136	-0.043	0.016	-0.074	-0.011	-1.644	431	844
L Pars opercularis	0.828	-0.010	-0.002	0.009	-0.021	0.016	-0.085	434	844
R Pars opercularis	0.217	-0.060	-0.013	0.010	-0.032	0.007	-0.521	433	845
L Pars orbitalis	0.068	-0.094	-0.023	0.013	-0.048	0.002	-0.910	425	841
R Pars orbitalis	0.165	-0.068	-0.018	0.013	-0.042	0.007	-0.697	427	842
L Pars triangularis	0.479	-0.034	-0.007	0.010	-0.026	0.012	-0.297	425	844
R Pars triangularis	0.661	-0.020	-0.004	0.009	-0.022	0.014	-0.179	427	846
L Pericalcarine	0.533	-0.027	-0.005	0.008	-0.022	0.011	-0.334	411	807
R Pericalcarine	0.442	-0.030	-0.007	0.009	-0.025	0.011	-0.440	395	771
L Postcentral	0.009	-0.120	-0.022	0.008	-0.039	-0.005	-1.117	404	813
R Postcentral	0.014	-0.107	-0.021	0.008	-0.037	-0.004	-1.070	409	816
L Posterior cingulate	0.122	-0.073	-0.015	0.010	-0.035	0.004	-0.643	434	843
R Posterior cingulate	0.014	-0.109	-0.025	0.010	-0.045	-0.005	-1.053	434	845
L Precentral	0.009	-0.127	-0.027	0.011	-0.048	-0.007	-1.140	412	812
R Precentral	0.037	-0.106	-0.023	0.011	-0.045	-0.001	-0.981	421	813
L Precuneus	0.002	-0.137	-0.027	0.009	-0.044	-0.010	-1.196	430	844
**R Precuneus**	**<0.001**	**-0.168**	**-0.033**	**0.009**	**-0.050**	**-0.016**	**-1.461**	**431**	**844**
L Rostral anterior cingulate	0.885	-0.007	-0.002	0.015	-0.032	0.027	-0.081	426	835
R Rostral anterior cingulate	0.383	-0.041	-0.013	0.014	-0.041	0.016	-0.472	428	837
L Rostral middle frontal	0.032	-0.095	-0.017	0.008	-0.033	-0.002	-0.769	424	841

R Rostral middle frontal	0.690	-0.016	-0.004	0.009	-0.021	0.014	-0.162	427	842
L Superior frontal	0.012	-0.121	-0.024	0.009	-0.042	-0.005	-0.924	423	839
R Superior frontal	0.014	-0.113	-0.023	0.009	-0.041	-0.005	-0.899	423	840
L Superior parietal	0.013	-0.116	-0.022	0.009	-0.038	-0.005	-1.037	416	826
**R Superior parietal**	**<0.001**	**-0.170**	**-0.036**	**0.009**	**-0.053**	**-0.018**	**-1.708**	**426**	**837**
L Superior temporal	0.082	-0.087	-0.019	0.011	-0.041	0.002	-0.740	378	766
R Superior temporal	0.332	-0.041	-0.010	0.011	-0.031	0.010	-0.391	404	800
L Supramarginal	0.071	-0.090	-0.018	0.010	-0.037	0.002	-0.754	413	809
R Supramarginal	0.016	-0.114	-0.023	0.010	-0.043	-0.004	-0.977	419	829
L Temporal pole	0.144	-0.078	-0.029	0.020	-0.068	0.010	-0.812	413	829
R Temporal pole	0.965	0.002	0.001	0.020	-0.039	0.041	0.025	416	819
L Transverse temporal	0.905	0.006	0.002	0.014	-0.026	0.029	0.077	434	844
R Transverse temporal	0.975	0.001	<0.001	0.015	-0.029	0.030	0.021	435	845

Results of cortical thickness analysis of HY1 stage PD vs matched healthy controls. L – Left, R – Right, ROI – region of interest, Banks STS – Banks of the superior temporal sulcus. **Boldface** denotes significant results after Bonferroni correction

Supplementary Table 4b: Cortical thickness results – HY2 PD vs controls

ROI Thickness	*p*	*d*	*b*	SE	Lower CI	Upper CI	% diff	NPD	NHC
L Banks STS	0.006	-0.104	-0.027	0.010	-0.047	-0.008	-1.184	819	832
R Banks STS	0.005	-0.109	-0.029	0.010	-0.048	-0.009	-1.185	887	870
L Caudal anterior cingulate	0.150	0.060	0.021	0.015	-0.008	0.050	0.815	928	901
R Caudal anterior cingulate	0.980	0.001	<0.001	0.013	-0.025	0.026	0.014	926	906
L Caudal middle frontal	0.011	-0.100	-0.021	0.008	-0.038	-0.005	-0.884	933	898
R Caudal middle frontal	0.321	-0.041	-0.008	0.008	-0.025	0.008	-0.354	929	898
L Cuneus	0.002	-0.121	-0.024	0.008	-0.038	-0.009	-1.335	871	867
R Cuneus	0.018	-0.085	-0.019	0.008	-0.035	-0.003	-1.054	841	843
L Entorhinal	0.005	-0.115	-0.049	0.018	-0.083	-0.015	-1.518	912	899
R Entorhinal	<0.001	-0.117	-0.063	0.019	-0.100	-0.026	-1.858	881	883
L Frontal pole	0.950	0.003	0.001	0.014	-0.027	0.029	0.034	935	902
R Frontal pole	0.106	-0.068	-0.023	0.014	-0.051	0.005	-0.879	932	905
**L Fusiform**	**<0.001**	**-0.173**	**-0.039**	**0.009**	**-0.057**	**-0.022**	**-1.531**	**922**	**901**
**R Fusiform**	**<0.001**	**-0.132**	**-0.041**	**0.010**	**-0.060**	**-0.021**	**-1.556**	**930**	**904**
**L Inferior parietal**	**<0.001**	**-0.175**	**-0.036**	**0.008**	**-0.051**	**-0.020**	**-1.558**	**899**	**875**
**R Inferior parietal**	**<0.001**	**-0.172**	**-0.038**	**0.008**	**-0.054**	**-0.022**	**-1.629**	**901**	**890**
**L Inferior temporal**	**<0.001**	**-0.141**	**-0.032**	**0.009**	**-0.050**	**-0.015**	**-1.219**	**905**	**867**
R Inferior temporal	0.004	-0.107	-0.027	0.009	-0.044	-0.009	-0.988	910	876
L Insula	0.134	-0.064	-0.015	0.010	-0.034	0.005	-0.511	934	901
R Insula	0.049	-0.077	-0.021	0.011	-0.042	<0.001	-0.739	926	897
**L Isthmus cingulate**	**<0.001**	**-0.160**	**-0.041**	**0.011**	**-0.061**	**-0.020**	**-1.731**	**933**	**904**
**R Isthmus cingulate**	**<0.001**	**-0.173**	**-0.046**	**0.011**	**-0.066**	**-0.025**	**-1.974**	**928**	**906**
L Lateral occipital	0.001	-0.131	-0.025	0.008	-0.041	-0.010	-1.220	921	883
**R Lateral occipital**	**<0.001**	**-0.136**	**-0.029**	**0.008**	**-0.046**	**-0.013**	**-1.359**	**916**	**891**
L Lateral orbitofrontal	0.054	-0.073	-0.016	0.008	-0.033	<0.001	-0.647	936	900
R Lateral orbitofrontal	0.005	-0.100	-0.024	0.009	-0.041	-0.007	-0.980	931	901
L Lingual	0.006	-0.105	-0.020	0.007	-0.034	-0.006	-1.028	903	891
R Lingual	0.043	-0.072	-0.015	0.008	-0.030	<0.001	-0.773	884	865

L Medial orbitofrontal	0.007	-0.111	-0.023	0.009	-0.040	-0.006	-0.996	924	898
R Medial orbitofrontal	0.124	-0.058	-0.014	0.009	-0.032	0.004	-0.608	919	899
**L Middle temporal**	**<0.001**	**-0.136**	**-0.032**	**0.010**	**-0.051**	**-0.014**	**-1.214**	**854**	**846**
R Middle temporal	0.009	-0.095	-0.024	0.009	-0.042	-0.006	-0.896	901	874
L Paracentral	0.008	-0.103	-0.025	0.010	-0.044	-0.007	-1.134	929	901
R Paracentral	0.113	-0.061	-0.015	0.010	-0.034	0.004	-0.677	922	904
**L Parahippocampal**	**<0.001**	**-0.173**	**-0.066**	**0.016**	**-0.097**	**-0.035**	**-2.533**	**937**	**902**
R Parahippocampal	0.005	-0.114	-0.038	0.014	-0.065	-0.012	-1.487	925	905
L Pars opercularis	0.902	0.005	0.001	0.008	-0.015	0.017	0.041	935	905
R Pars opercularis	0.262	-0.043	-0.009	0.008	-0.026	0.007	-0.391	932	906
L Pars orbitalis	0.109	-0.064	-0.017	0.011	-0.038	0.004	-0.668	931	903
R Pars orbitalis	0.059	-0.075	-0.020	0.010	-0.040	0.001	-0.787	929	904
L Pars triangularis	0.443	-0.029	-0.006	0.008	-0.022	0.010	-0.278	935	905
R Pars triangularis	0.241	-0.044	-0.009	0.008	-0.025	0.006	-0.411	927	907
L Pericalcarine	0.016	-0.086	-0.017	0.007	-0.031	-0.003	-1.092	865	870
R Pericalcarine	0.128	-0.051	-0.012	0.008	-0.028	0.003	-0.761	828	827
L Postcentral	0.011	-0.095	-0.018	0.007	-0.032	-0.004	-0.913	890	864
R Postcentral	0.006	-0.099	-0.020	0.007	-0.034	-0.006	-1.021	884	871
L Posterior cingulate	0.151	-0.057	-0.013	0.009	-0.030	0.005	-0.537	935	904
R Posterior cingulate	0.001	-0.122	-0.029	0.009	-0.046	-0.012	-1.227	934	905
L Precentral	0.070	-0.071	-0.017	0.009	-0.035	0.001	-0.702	898	860
R Precentral	0.309	-0.040	-0.010	0.009	-0.028	0.009	-0.412	896	867
**L Precuneus**	**<0.001**	**-0.150**	**-0.032**	**0.008**	**-0.047**	**-0.017**	**-1.427**	**934**	**904**
**R Precuneus**	**<0.001**	**-0.183**	**-0.040**	**0.008**	**-0.055**	**-0.024**	**-1.769**	**930**	**905**
L Rostral anterior cingulate	0.649	0.020	0.006	0.013	-0.020	0.031	0.218	922	895
R Rostral anterior cingulate	0.881	-0.006	-0.002	0.013	-0.027	0.023	-0.071	919	897
L Rostral middle frontal	0.360	-0.033	-0.006	0.007	-0.020	0.007	-0.286	924	901
R Rostral middle frontal	0.972	-0.001	<0.001	0.007	-0.015	0.014	-0.011	929	901
L Superior frontal	0.045	-0.078	-0.016	0.008	-0.032	<0.001	-0.629	909	894
R Superior frontal	0.010	-0.096	-0.020	0.008	-0.035	-0.005	-0.790	910	894
**L Superior parietal**	**<0.001**	**-0.134**	**-0.028**	**0.007**	**-0.042**	**-0.013**	**-1.337**	**904**	**888**
**R Superior parietal**	**<0.001**	**-0.151**	**-0.033**	**0.008**	**-0.048**	**-0.018**	**-1.618**	**908**	**898**
L Superior temporal	0.009	-0.102	-0.024	0.009	-0.043	-0.006	-0.945	802	824
R Superior temporal	0.002	-0.107	-0.028	0.009	-0.045	-0.011	-1.084	846	866
L Supramarginal	0.018	-0.093	-0.020	0.008	-0.036	-0.003	-0.836	886	867
**R Supramarginal**	**<0.001**	**-0.146**	**-0.032**	**0.008**	**-0.048**	**-0.016**	**-1.340**	**898**	**888**
**L Temporal pole**	**<0.001**	**-0.158**	**-0.064**	**0.018**	**-0.099**	**-0.030**	**-1.811**	**890**	**886**
R Temporal pole	0.001	-0.122	-0.057	0.018	-0.091	-0.022	-1.560	889	879
L Transverse temporal	0.013	-0.102	-0.029	0.012	-0.053	-0.006	-1.355	939	905
R Transverse temporal	0.002	-0.097	-0.039	0.013	-0.064	-0.014	-1.735	937	907

Results of cortical thickness analysis of HY2 stage PD vs matched healthy controls. L – Left, R – Right, ROI – region of interest, Banks STS – Banks of the superior temporal sulcus. **Boldface** denotes significant results after Bonferroni correction

Supplementary Table 4c: Cortical thickness results – HY3 PD vs controls

ROI Thickness	*p*	*d*	*b*	SE	Lower CI	Upper CI	% diff	NPD	NHC
L Banks STS	0.007	-0.172	-0.047	0.017	-0.081	-0.013	-2.077	227	459
**R Banks STS**	**<0.001**	**-0.249**	**-0.065**	**0.016**	**-0.097**	**-0.034**	**-2.737**	**239**	**481**

L Caudal anterior cingulate	0.204	0.087	0.032	0.025	-0.017	0.080	1.226	255	497
R Caudal anterior cingulate	0.752	-0.021	-0.007	0.023	-0.052	0.038	-0.293	256	500
**L Caudal middle frontal**	**<0.001**	**-0.258**	**-0.055**	**0.014**	**-0.082**	**-0.028**	**-2.308**	**258**	**499**
R Caudal middle frontal	0.015	-0.160	-0.035	0.014	-0.063	-0.007	-1.469	256	495
L Cuneus	0.039	-0.129	-0.026	0.013	-0.052	-0.001	-1.521	223	475
R Cuneus	0.014	-0.130	-0.032	0.013	-0.057	-0.006	-1.786	228	458
**L Entorhinal**	**<0.001**	**-0.276**	**-0.118**	**0.029**	**-0.174**	**-0.062**	**-3.672**	**257**	**499**
**R Entorhinal**	**<0.001**	**-0.280**	**-0.159**	**0.032**	**-0.222**	**-0.097**	**-4.743**	**243**	**487**
L Frontal pole	0.964	0.003	0.001	0.023	-0.044	0.046	0.039	258	500
R Frontal pole	0.373	-0.062	-0.021	0.024	-0.069	0.026	-0.824	255	500
**L Fusiform**	<0.001	-0.372	-0.089	0.014	-0.117	-0.061	-3.476	252	499
**R Fusiform**	**<0.001**	**-0.204**	**-0.068**	**0.018**	**-0.103**	**-0.034**	**-2.632**	**253**	**499**
**L Inferior parietal**	**<0.001**	**-0.359**	**-0.077**	**0.013**	**-0.103**	**-0.051**	**-3.427**	**248**	**483**
**R Inferior parietal**	**<0.001**	**-0.321**	**-0.076**	**0.014**	**-0.102**	**-0.049**	**-3.295**	**250**	**492**
**L Inferior temporal**	**<0.001**	**-0.336**	**-0.077**	**0.014**	**-0.106**	**-0.049**	**-2.928**	**232**	**481**
**R Inferior temporal**	**<0.001**	**-0.273**	**-0.068**	**0.014**	**-0.097**	**-0.040**	**-2.566**	**240**	**480**
L Insula	0.895	0.009	0.002	0.016	-0.030	0.034	0.074	253	499
R Insula	0.244	-0.072	-0.021	0.018	-0.057	0.014	-0.744	254	494
**L Isthmus cingulate**	**<0.001**	**-0.283**	**-0.073**	**0.017**	**-0.107**	**-0.039**	**-3.154**	**257**	**501**
**R Isthmus cingulate**	**<0.001**	**-0.237**	**-0.064**	**0.017**	**-0.097**	**-0.031**	**-2.786**	**256**	**501**
**L Lateral occipital**	**<0.001**	**-0.220**	**-0.046**	**0.013**	**-0.071**	**-0.020**	**-2.210**	**246**	**489**
**R Lateral occipital**	**<0.001**	**-0.239**	**-0.055**	**0.014**	**-0.083**	**-0.026**	**-2.579**	**250**	**491**
L Lateral orbitofrontal	0.011	-0.166	-0.035	0.014	-0.062	-0.008	-1.397	258	500
R Lateral orbitofrontal	0.046	-0.114	-0.028	0.014	-0.056	-0.001	-1.152	257	499
**L Lingual**	**<0.001**	**-0.217**	**-0.044**	**0.012**	**-0.068**	**-0.020**	**-2.320**	**242**	**490**
R Lingual	0.024	-0.124	-0.029	0.013	-0.054	-0.004	-1.491	243	476
L Medial orbitofrontal	0.937	-0.005	-0.001	0.014	-0.029	0.027	-0.048	254	496
R Medial orbitofrontal	0.535	0.037	0.009	0.014	-0.019	0.037	0.391	254	493
**L Middle temporal**	**<0.001**	**-0.311**	**-0.078**	**0.016**	**-0.109**	**-0.048**	**-2.954**	**229**	**470**
**R Middle temporal**	**<0.001**	**-0.230**	**-0.060**	**0.015**	**-0.089**	**-0.031**	**-2.242**	**249**	**478**
**L Paracentral**	**<0.001**	**-0.211**	**-0.055**	**0.015**	**-0.085**	**-0.025**	**-2.466**	**255**	**498**
R Paracentral	0.026	-0.133	-0.035	0.016	-0.067	-0.004	-1.564	252	499
L Parahippocampal	0.007	-0.183	-0.074	0.028	-0.128	-0.020	-2.895	257	499
**R Parahippocampal**	**<0.001**	**-0.298**	**-0.108**	**0.023**	**-0.154**	**-0.063**	**-4.257**	**256**	**500**
L Pars opercularis	0.119	-0.095	-0.021	0.013	-0.047	0.005	-0.868	257	501
R Pars opercularis	0.106	-0.098	-0.022	0.014	-0.049	0.005	-0.924	257	501
L Pars orbitalis	0.061	-0.121	-0.035	0.018	-0.071	0.002	-1.371	258	499
R Pars orbitalis	0.574	-0.036	-0.010	0.018	-0.045	0.025	-0.402	256	499
L Pars triangularis	0.006	-0.165	-0.037	0.014	-0.064	-0.011	-1.663	258	501
R Pars triangularis	0.270	-0.065	-0.015	0.014	-0.042	0.012	-0.663	257	501
L Pericalcarine	0.607	-0.031	-0.007	0.013	-0.032	0.019	-0.436	217	473
R Pericalcarine	0.073	-0.094	-0.024	0.013	-0.051	0.002	-1.546	220	451
L Postcentral	0.186	-0.077	-0.016	0.012	-0.039	0.008	-0.810	246	481
R Postcentral	0.012	-0.142	-0.030	0.012	-0.053	-0.006	-1.553	243	481
L Posterior cingulate	0.145	-0.094	-0.021	0.015	-0.050	0.007	-0.907	257	500
**R Posterior cingulate**	**<0.001**	**-0.256**	**-0.064**	**0.015**	**-0.094**	**-0.034**	**-2.738**	**257**	**501**
**L Precentral**	**<0.001**	**-0.244**	**-0.060**	**0.015**	**-0.088**	**-0.031**	**-2.522**	**245**	**480**

R Precentral	<0.001	-0.202	-0.051	0.015	-0.081	-0.021	-2.194	246	477
**L Precuneus**	**<0.001**	**-0.267**	**-0.059**	**0.013**	**-0.085**	**-0.034**	**-2.701**	**255**	**501**
**R Precuneus**	**<0.001**	**-0.326**	**-0.072**	**0.013**	**-0.097**	**-0.047**	**-3.242**	**256**	**501**
L Rostral anterior cingulate	0.275	0.075	0.023	0.021	-0.018	0.064	0.838	255	494
R Rostral anterior cingulate	0.025	0.137	0.048	0.021	0.006	0.090	1.779	253	492
L Rostral middle frontal	0.018	-0.141	-0.029	0.012	-0.052	-0.005	-1.282	256	499
R Rostral middle frontal	0.822	-0.012	-0.003	0.013	-0.029	0.023	-0.135	257	498
L Superior frontal	0.001	-0.206	-0.042	0.013	-0.068	-0.017	-1.674	251	494
**R Superior frontal**	**<0.001**	**-0.220**	**-0.047**	**0.013**	**-0.073**	**-0.022**	**-1.887**	**250**	**495**
**L Superior parietal**	**<0.001**	**-0.226**	**-0.050**	**0.013**	**-0.076**	**-0.025**	**-2.463**	**242**	**495**
**R Superior parietal**	**<0.001**	**-0.261**	**-0.064**	**0.013**	**-0.089**	**-0.038**	**-3.109**	**245**	**496**
**L Superior temporal**	**<0.001**	**-0.304**	**-0.073**	**0.015**	**-0.103**	**-0.043**	**-2.854**	**210**	**454**
**R Superior temporal**	**<0.001**	**-0.191**	**-0.054**	**0.015**	**-0.083**	**-0.024**	**-2.099**	**237**	**478**
**L Supramarginal**	**<0.001**	**-0.265**	**-0.057**	**0.013**	**-0.083**	**-0.031**	**-2.424**	**244**	**477**
**R Supramarginal**	**<0.001**	**-0.300**	**-0.068**	**0.014**	**-0.094**	**-0.041**	**-2.854**	**248**	**491**
L Temporal pole	0.103	-0.117	-0.044	0.027	-0.098	0.009	-1.257	237	494
**R Temporal pole**	**<0.001**	**-0.198**	**-0.093**	**0.027**	**-0.147**	**-0.040**	**-2.573**	**240**	**487**
L Transverse temporal	0.003	-0.194	-0.061	0.020	-0.100	-0.021	-2.808	258	501
**R Transverse temporal**	**<0.001**	**-0.167**	**-0.074**	**0.021**	**-0.116**	**-0.032**	**-3.268**	**257**	**500**

Results of cortical thickness analysis of HY3 stage PD vs matched healthy controls. L – Left, R – Right, ROI – region of interest, Banks STS – Banks of the superior temporal sulcus. **Boldface** denotes significant results after Bonferroni correction

Supplementary Table 4d: Cortical thickness results - HY4&5 PD vs controls

ROI Thickness	*p*	*d*	*b*	SE	Lower CI	Upper CI	% diff	NPD	NHC
**L Banks STS**	**<0.001**	**-0.473**	**-0.115**	**0.026**	**-0.167**	**-0.064**	**-5.154**	**75**	**301**
R Banks STS	<0.001	-0.346	-0.084	0.025	-0.132	-0.035	-3.554	78	318
L Caudal anterior cingulate	0.067	0.205	0.071	0.039	-0.005	0.147	2.732	82	327
R Caudal anterior cingulate	0.051	-0.212	-0.067	0.034	-0.134	<0.001	-2.708	83	329
**L Caudal middle frontal**	**<0.001**	**-0.379**	**-0.076**	**0.021**	**-0.118**	**-0.035**	**-3.231**	**82**	**327**
R Caudal middle frontal	0.366	-0.099	-0.020	0.022	-0.064	0.024	-0.863	82	326
L Cuneus	0.131	-0.145	-0.027	0.018	-0.062	0.008	-1.560	76	309
R Cuneus	0.449	-0.067	-0.015	0.020	-0.053	0.024	-0.845	75	296
**L Entorhinal**	**<0.001**	**-0.399**	**-0.166**	**0.044**	**-0.253**	**-0.079**	**-5.243**	**83**	**325**
**R Entorhinal**	**<0.001**	**-0.370**	**-0.195**	**0.047**	**-0.287**	**-0.103**	**-5.753**	**83**	**317**
L Frontal pole	0.438	-0.089	-0.026	0.034	-0.093	0.040	-1.009	83	325
R Frontal pole	<0.001	-0.368	-0.118	0.035	-0.188	-0.048	-4.557	83	326
**L Fusiform**	**<0.001**	**-0.583**	**-0.122**	**0.021**	**-0.164**	**-0.081**	**-4.834**	**83**	**328**
**R Fusiform**	**<0.001**	**-0.407**	**-0.127**	**0.027**	**-0.180**	**-0.074**	**-4.938**	**81**	**329**
**L Inferior parietal**	**<0.001**	**-0.522**	**-0.097**	**0.019**	**-0.134**	**-0.061**	**-4.355**	**80**	**312**
**R Inferior parietal**	**<0.001**	**-0.517**	**-0.111**	**0.021**	**-0.152**	**-0.070**	**-4.869**	**82**	**321**
**L Inferior temporal**	**<0.001**	**-0.474**	**-0.093**	**0.020**	**-0.133**	**-0.054**	**-3.582**	**81**	**317**
**R Inferior temporal**	**<0.001**	**-0.466**	**-0.102**	**0.021**	**-0.143**	**-0.061**	**-3.870**	**81**	**324**
L Insula	0.027	-0.246	-0.057	0.026	-0.107	-0.007	-1.969	83	327
**R Insula**	**<0.001**	**-0.393**	**-0.112**	**0.029**	**-0.169**	**-0.055**	**-3.942**	**83**	**326**
**L Isthmus cingulate**	**<0.001**	**-0.427**	**-0.103**	**0.025**	**-0.153**	**-0.053**	**-4.419**	**83**	**327**
**R Isthmus cingulate**	**<0.001**	**-0.366**	**-0.091**	**0.025**	**-0.140**	**-0.042**	**-3.960**	**83**	**329**

L Lateral occipital	0.113	-0.164	-0.032	0.020	-0.071	0.008	-1.546	78	319
R Lateral occipital	0.002	-0.320	-0.066	0.021	-0.108	-0.024	-3.133	80	321
**L Lateral orbitofrontal**	**<0.001**	**-0.402**	**-0.072**	**0.019**	**-0.108**	**-0.035**	**-2.883**	**83**	**328**
**R Lateral orbitofrontal**	**<0.001**	**-0.484**	**-0.106**	**0.020**	**-0.145**	**-0.066**	**-4.325**	**83**	**328**
L Lingual	0.002	-0.287	-0.054	0.018	-0.089	-0.019	-2.880	79	325
R Lingual	0.140	-0.129	-0.028	0.019	-0.065	0.009	-1.444	77	317
L Medial orbitofrontal	0.016	-0.260	-0.051	0.021	-0.093	-0.009	-2.209	83	326
**R Medial orbitofrontal**	**<0.001**	**-0.366**	**-0.081**	**0.022**	**-0.124**	**-0.038**	**-3.568**	**83**	**326**
**L Middle temporal**	**<0.001**	**-0.484**	**-0.108**	**0.023**	**-0.154**	**-0.063**	**-4.152**	**77**	**308**
**R Middle temporal**	**<0.001**	**-0.461**	**-0.105**	**0.022**	**-0.148**	**-0.063**	**-3.976**	**82**	**318**
L Paracentral	0.047	-0.185	-0.046	0.023	-0.090	-0.001	-2.081	83	326
R Paracentral	0.548	-0.058	-0.015	0.025	-0.064	0.034	-0.673	83	326
L Parahippocampal	0.001	-0.360	-0.131	0.040	-0.210	-0.051	-5.137	82	327
R Parahippocampal	0.001	-0.351	-0.114	0.035	-0.183	-0.045	-4.501	83	328
L Pars opercularis	0.002	-0.310	-0.061	0.020	-0.099	-0.022	-2.583	83	327
R Pars opercularis	0.005	-0.300	-0.058	0.020	-0.098	-0.018	-2.466	83	329
L Pars orbitalis	0.007	-0.282	-0.070	0.025	-0.120	-0.020	-2.814	83	327
R Pars orbitalis	0.013	-0.270	-0.069	0.028	-0.124	-0.015	-2.801	82	328
L Pars triangularis	0.022	-0.237	-0.045	0.020	-0.083	-0.007	-2.022	82	328
R Pars triangularis	0.462	-0.076	-0.015	0.021	-0.056	0.026	-0.687	83	329
L Pericalcarine	0.349	-0.090	-0.017	0.018	-0.053	0.019	-1.138	76	311
R Pericalcarine	0.953	-0.005	-0.001	0.019	-0.038	0.036	-0.072	71	294
**L Postcentral**	**<0.001**	**-0.343**	**-0.061**	**0.017**	**-0.094**	**-0.028**	**-3.165**	**81**	**309**
R Postcentral	<0.001	-0.306	-0.056	0.017	-0.088	-0.023	-2.935	81	314
L Posterior cingulate	0.025	-0.236	-0.051	0.023	-0.095	-0.006	-2.146	83	328
**R Posterior cingulate**	**<0.001**	**-0.463**	**-0.107**	**0.023**	**-0.152**	**-0.062**	**-4.613**	**83**	**329**
L Precentral	0.008	-0.265	-0.060	0.022	-0.104	-0.016	-2.565	80	309
R Precentral	0.139	-0.141	-0.033	0.022	-0.076	0.011	-1.414	81	312
**L Precuneus**	**<0.001**	**-0.421**	**-0.083**	**0.018**	**-0.119**	**-0.047**	**-3.837**	**82**	**327**
**R Precuneus**	**<0.001**	**-0.449**	**-0.090**	**0.019**	**-0.128**	**-0.053**	**-4.125**	**83**	**328**
L Rostral anterior cingulate	0.454	-0.085	-0.023	0.030	-0.082	0.037	-0.836	83	325
R Rostral anterior cingulate	0.346	-0.094	-0.029	0.031	-0.089	0.031	-1.078	83	325
L Rostral middle frontal	0.012	-0.243	-0.041	0.016	-0.074	-0.009	-1.888	83	328
R Rostral middle frontal	0.002	-0.265	-0.054	0.018	-0.089	-0.020	-2.486	82	324
L Superior frontal	0.046	-0.209	-0.039	0.020	-0.078	-0.001	-1.561	81	322
R Superior frontal	0.105	-0.163	-0.031	0.019	-0.068	0.007	-1.252	81	320
L Superior parietal	0.001	-0.305	-0.059	0.018	-0.095	-0.023	-2.911	83	317
**R Superior parietal**	**<0.001**	**-0.312**	**-0.065**	**0.018**	**-0.101**	**-0.029**	**-3.194**	**83**	**321**
**L Superior temporal**	**<0.001**	**-0.467**	**-0.103**	**0.023**	**-0.148**	**-0.058**	**-4.102**	**76**	**297**
**R Superior temporal**	**<0.001**	**-0.368**	**-0.091**	**0.021**	**-0.133**	**-0.049**	**-3.624**	**79**	**312**
**L Supramarginal**	**<0.001**	**-0.495**	**-0.096**	**0.020**	**-0.135**	**-0.057**	**-4.155**	**83**	**311**
**R Supramarginal**	**<0.001**	**-0.521**	**-0.109**	**0.021**	**-0.150**	**-0.068**	**-4.656**	**82**	**320**
L Temporal pole	0.001	-0.361	-0.131	0.040	-0.210	-0.052	-3.746	80	324
**R Temporal pole**	**<0.001**	**-0.428**	**-0.177**	**0.040**	**-0.256**	**-0.098**	**-4.892**	**82**	**316**
**L Transverse temporal**	**<0.001**	**-0.367**	**-0.104**	**0.030**	**-0.162**	**-0.046**	**-4.922**	**83**	**328**
R Transverse temporal	0.056	-0.153	-0.063	0.033	-0.129	0.002	-2.881	82	329

Results of cortical thickness analysis of HY stage 4 and 5 PD vs matched healthy controls. L – Left, R – Right, ROI – region of interest, Banks STS – Banks of the superior temporal sulcus. **Boldface** denotes significant results after Bonferroni correction

Supplementary Table 4e: Cortical surface area results - HY1 PD vs controls

ROI Surface Area	*p*	*d*	*b*	SE	Lower CI	Upper CI	% diff	NPD	NHC
L Banks STS	0.942	-0.004	-0.68	9.34	-19.00	17.65	-0.07	383	776
R Banks STS	0.441	0.043	6.00	7.78	-9.27	21.26	0.66	417	813
L Caudal anterior cingulate	0.352	0.053	7.04	7.56	-7.80	21.87	1.13	432	839
R Caudal anterior cingulate	0.562	0.033	5.16	8.91	-12.31	22.63	0.68	430	842
L Caudal middle frontal	0.178	0.076	25.80	19.14	-11.75	63.34	1.17	429	838
R Caudal middle frontal	0.665	0.025	8.44	19.50	-29.81	46.69	0.41	430	839
L Cuneus	0.074	-0.106	-22.06	12.32	-46.23	2.11	-1.52	404	802
R Cuneus	0.009	-0.155	-32.27	12.35	-56.50	-8.03	-2.14	400	770
L Entorhinal	0.411	-0.048	-4.17	5.07	-14.13	5.78	-1.01	425	836
R Entorhinal	0.014	-0.141	-11.02	4.49	-19.82	-2.21	-3.09	419	823
**L Frontal pole**	**<0.001**	**-0.219**	**-8.25**	**1.98**	**-12.12**	**-4.37**	**-3.93**	**428**	**842**
R Frontal pole	0.001	-0.169	-7.94	2.48	-12.81	-3.07	-2.87	429	843
L Fusiform	0.344	0.054	19.69	20.81	-21.15	60.52	0.63	425	841
R Fusiform	0.922	0.005	2.02	20.50	-38.21	42.24	0.07	430	842
L Inferior parietal	0.607	-0.029	-16.47	32.01	-79.28	46.34	-0.37	426	814
R Inferior parietal	0.174	0.076	51.64	37.93	-22.78	126.07	0.99	421	830
L Inferior temporal	0.603	0.029	13.16	25.34	-36.55	62.87	0.41	409	803
R Inferior temporal	0.877	-0.009	-3.61	23.28	-49.28	42.06	-0.12	423	811
L Insula	0.914	-0.006	-1.37	12.69	-26.27	23.54	-0.06	430	832
R Insula	0.648	-0.025	-6.52	14.29	-34.56	21.52	-0.29	429	829
L Isthmus cingulate	0.102	0.091	15.34	9.38	-3.07	33.74	1.54	433	841
R Isthmus cingulate	0.189	0.072	11.21	8.53	-5.52	27.94	1.22	432	843
L Lateral occipital	0.496	-0.038	-22.32	32.78	-86.63	41.99	-0.47	421	827
R Lateral occipital	0.164	-0.078	-45.95	33.02	-110.73	18.84	-1.00	426	830
L Lateral orbitofrontal	0.530	0.035	9.22	14.69	-19.60	38.04	0.37	426	840
R Lateral orbitofrontal	0.032	0.117	34.39	16.05	2.90	65.88	1.39	428	841
L Lingual	0.320	0.056	23.46	23.60	-22.84	69.75	0.78	426	826
R Lingual	0.693	-0.023	-9.31	23.57	-55.54	36.93	-0.31	418	801
L Medial orbitofrontal	0.958	0.003	0.64	12.20	-23.30	24.59	0.04	425	830
R Medial orbitofrontal	0.708	-0.020	-3.91	10.43	-24.38	16.56	-0.22	425	833
L Middle temporal	0.100	-0.095	-35.15	21.35	-77.03	6.74	-1.18	386	782
R Middle temporal	0.744	-0.019	-6.89	21.07	-48.23	34.45	-0.21	417	812
L Paracentral	0.241	0.066	12.18	10.38	-8.19	32.54	0.92	432	840
R Paracentral	0.218	0.068	13.73	11.14	-8.13	35.58	0.91	431	844
L Parahippocampal	0.173	0.078	8.05	5.91	-3.54	19.63	1.19	431	842
R Parahippocampal	0.079	0.099	8.67	4.93	-1.01	18.35	1.33	430	844
L Pars opercularis	0.865	-0.010	-2.41	14.15	-30.17	25.34	-0.15	435	842
R Pars opercularis	0.071	0.105	22.64	12.53	-1.95	47.22	1.68	433	842
L Pars orbitalis	0.774	-0.016	-1.23	4.30	-9.66	7.19	-0.20	425	841
R Pars orbitalis	0.459	0.042	4.09	5.52	-6.74	14.93	0.55	427	842
L Pars triangularis	0.729	0.020	3.57	10.29	-16.61	23.76	0.29	425	843
R Pars triangularis	0.425	0.046	10.38	13.01	-15.14	35.89	0.72	427	844
L Pericalcarine	0.551	-0.035	-8.25	13.83	-35.39	18.89	-0.60	411	798
R Pericalcarine	0.881	-0.009	-2.25	15.06	-31.80	27.30	-0.15	395	753
L Postcentral	0.709	0.021	9.05	24.23	-38.49	56.58	0.22	404	815

R Postcentral	0.111	-0.090	-37.99	23.84	-84.76	8.78	-0.97	409	818
L Posterior cingulate	0.217	0.070	12.48	10.10	-7.34	32.31	1.10	434	839
R Posterior cingulate	0.337	0.053	9.70	10.10	-10.12	29.51	0.84	435	843
L Precentral	0.819	-0.013	-6.04	26.39	-57.82	45.75	-0.13	412	812
R Precentral	0.683	0.023	10.98	26.90	-41.80	63.76	0.23	421	816
L Precuneus	0.222	-0.067	-28.64	23.46	-74.66	17.39	-0.78	431	842
R Precuneus	0.784	-0.015	-6.83	24.86	-55.61	41.95	-0.18	431	842
L Rostral anterior cingulate	0.989	0.001	0.12	8.42	-16.41	16.64	0.01	425	813
R Rostral anterior cingulate	0.535	0.034	4.71	7.60	-10.20	19.63	0.71	428	831
L Rostral middle frontal	0.728	0.019	12.19	35.00	-56.48	80.86	0.22	424	842
R Rostral middle frontal	0.768	-0.016	-11.46	38.84	-87.67	64.75	-0.20	427	842
L Superior frontal	0.793	0.014	10.10	38.48	-65.39	85.58	0.15	423	826
R Superior frontal	0.493	0.038	25.77	37.56	-47.92	99.46	0.38	423	838
L Superior parietal	0.987	-0.001	-0.56	34.19	-67.63	66.51	-0.01	416	826
R Superior parietal	0.957	0.003	1.82	34.05	-64.99	68.62	0.03	426	836
L Superior temporal	0.393	-0.050	-19.17	22.44	-63.19	24.86	-0.52	378	762
R Superior temporal	0.810	0.014	4.65	19.30	-33.23	42.53	0.13	404	801
L Supramarginal	0.259	0.065	34.26	30.34	-25.27	93.79	0.91	412	807
R Supramarginal	0.873	0.009	4.33	27.17	-48.97	57.63	0.12	420	828
L Temporal pole	0.278	-0.059	-3.76	3.47	-10.56	3.04	-0.79	413	829
R Temporal pole	0.446	-0.043	-2.78	3.65	-9.94	4.37	-0.66	416	819
L Transverse temporal	0.271	0.061	4.76	4.32	-3.72	13.25	1.05	434	844
R Transverse temporal	0.529	0.036	1.89	3.00	-4.00	7.79	0.57	435	846

Results of cortical surface area analysis of HY stage 1 PD vs matched healthy controls. L – Left, R – Right, ROI – region of interest, Banks STS – Banks of the superior temporal sulcus. **Boldface** denotes significant results after Bonferroni correction

Supplementary Table 4f: Cortical surface area results - HY2 PD vs controls

ROI Surface Area	*p*	*d*	*b*	SE	Lower CI	Upper CI	% diff	NPD	NHC
L Banks STS	0.260	0.052	8.76	7.77	-6.48	24.00	0.88	817	821
R Banks STS	0.070	0.080	12.04	6.64	-0.98	25.06	1.33	888	874
L Caudal anterior cingulate	0.080	0.079	11.20	6.40	-1.36	23.76	1.80	928	900
R Caudal anterior cingulate	0.858	-0.008	-1.33	7.46	-15.97	13.31	-0.18	924	905
L Caudal middle frontal	0.615	-0.022	-7.93	15.76	-38.84	22.98	-0.36	932	897
R Caudal middle frontal	0.457	-0.034	-12.20	16.40	-44.37	19.96	-0.59	930	897
L Cuneus	0.001	-0.156	-33.62	9.98	-53.19	-14.05	-2.32	871	861
R Cuneus	0.002	-0.146	-31.86	10.25	-51.96	-11.77	-2.13	838	826
L Entorhinal	0.644	0.021	1.96	4.25	-6.36	10.29	0.48	912	898
R Entorhinal	0.665	-0.020	-1.69	3.91	-9.36	5.97	-0.48	881	881
L Frontal pole	0.003	-0.121	-4.92	1.65	-8.15	-1.69	-2.37	934	902
R Frontal pole	0.979	0.001	0.05	2.05	-3.97	4.07	0.02	933	905
L Fusiform	0.263	-0.049	-19.80	17.68	-54.48	14.88	-0.64	921	901
R Fusiform	0.076	-0.077	-31.43	17.70	-66.14	3.28	-1.03	929	903
L Inferior parietal	0.624	-0.022	-13.16	26.83	-65.79	39.47	-0.29	899	874
R Inferior parietal	0.561	-0.026	-17.65	30.37	-77.21	41.91	-0.34	902	890
L Inferior temporal	0.303	-0.045	-21.24	20.63	-61.70	19.21	-0.67	907	864

R Inferior temporal	0.492	-0.030	-13.52	19.69	-52.13	25.09	-0.44	910	875
L Insula	0.590	0.022	5.64	10.46	-14.87	26.14	0.26	934	896
R Insula	0.737	-0.014	-4.13	12.32	-28.29	20.02	-0.18	926	892
L Isthmus cingulate	0.578	0.024	4.33	7.78	-10.93	19.59	0.43	934	903
R Isthmus cingulate	0.428	0.034	5.43	6.85	-8.01	18.87	0.58	928	906
L Lateral occipital	0.001	-0.148	-94.35	27.52	-148.32	-40.39	-1.99	921	883
R Lateral occipital	0.003	-0.129	-81.27	27.78	-135.76	-26.78	-1.76	917	889
L Lateral orbitofrontal	0.071	-0.079	-22.71	12.57	-47.36	1.93	-0.90	935	900
R Lateral orbitofrontal	0.895	-0.006	-1.80	13.63	-28.53	24.93	-0.07	930	902
**L Lingual**	**0.001**	**-0.154**	**-69.27**	**19.93**	**-108.35**	**-30.18**	**-2.32**	**903**	**889**
**R Lingual**	**<0.001**	**-0.175**	**-77.65**	**19.84**	**-116.57**	**-38.74**	**-2.57**	**883**	**858**
L Medial orbitofrontal	0.548	-0.026	-6.20	10.33	-26.47	14.06	-0.34	924	892
R Medial orbitofrontal	0.232	-0.052	-10.75	9.00	-28.41	6.90	-0.60	920	895
L Middle temporal	0.064	-0.084	-33.40	18.04	-68.79	1.98	-1.12	854	837
R Middle temporal	0.704	-0.017	-6.90	18.18	-42.56	28.76	-0.21	901	874
L Paracentral	0.084	0.078	14.94	8.64	-2.00	31.89	1.12	928	901
R Paracentral	0.215	0.055	12.06	9.72	-6.99	31.12	0.80	923	903
L Parahippocampal	0.197	0.055	6.31	4.89	-3.27	15.90	0.93	936	903
R Parahippocampal	0.202	0.056	5.96	4.67	-3.20	15.12	0.91	924	905
L Pars opercularis	0.806	-0.011	-2.79	11.32	-25.00	19.42	-0.18	935	903
R Pars opercularis	0.319	-0.045	-9.81	9.84	-29.11	9.49	-0.73	933	902
L Pars orbitalis	0.088	-0.074	-6.34	3.72	-13.64	0.95	-1.03	930	903
R Pars orbitalis	0.388	0.038	4.08	4.72	-5.18	13.34	0.54	930	904
L Pars triangularis	0.591	-0.024	-4.82	8.97	-22.41	12.78	-0.39	935	904
R Pars triangularis	0.316	-0.045	-10.88	10.84	-32.13	10.38	-0.76	927	904
L Pericalcarine	0.001	-0.147	-36.57	11.49	-59.10	-14.03	-2.70	864	862
**R Pericalcarine**	**<0.001**	**-0.168**	**-44.85**	**12.40**	**-69.18**	**-20.53**	**-3.03**	**827**	**807**
L Postcentral	0.294	0.047	21.60	20.60	-18.80	62.01	0.53	890	866
R Postcentral	0.390	0.039	17.46	20.32	-22.39	57.31	0.44	883	873
L Posterior cingulate	0.634	0.021	4.34	9.10	-13.52	22.20	0.38	937	902
R Posterior cingulate	0.681	0.018	3.39	8.23	-12.75	19.53	0.29	933	904
L Precentral	0.702	0.017	8.42	22.03	-34.79	51.63	0.17	896	860
R Precentral	0.151	0.063	32.66	22.72	-11.91	77.23	0.68	896	869
L Precuneus	0.171	-0.059	-27.24	19.91	-66.30	11.81	-0.74	934	904
R Precuneus	0.772	0.012	6.04	20.87	-34.89	46.96	0.16	930	904
L Rostral anterior cingulate	0.685	0.018	2.99	7.38	-11.48	17.47	0.37	918	880
R Rostral anterior cingulate	0.525	-0.027	-4.09	6.44	-16.71	8.53	-0.61	921	894
L Rostral middle frontal	0.033	-0.092	-66.31	31.11	-127.33	-5.29	-1.22	924	902
R Rostral middle frontal	0.042	-0.088	-65.22	32.02	-128.02	-2.42	-1.16	931	901
L Superior frontal	0.013	-0.106	-83.00	33.52	-148.73	-17.26	-1.19	910	886
R Superior frontal	0.236	-0.052	-39.64	33.46	-105.27	25.98	-0.59	910	893
L Superior parietal	0.009	-0.117	-74.87	28.56	-130.89	-18.85	-1.41	904	888
R Superior parietal	0.105	-0.072	-45.76	28.24	-101.14	9.63	-0.86	906	897
L Superior temporal	0.427	-0.037	-15.76	19.85	-54.69	23.17	-0.43	800	816
R Superior temporal	0.268	0.049	18.41	16.62	-14.19	51.01	0.53	846	866

L Supramarginal	0.720	0.016	8.97	25.03	-40.12	58.06	0.24	887	865
R Supramarginal	0.285	0.047	24.45	22.88	-20.42	69.32	0.68	900	890
L Temporal pole	0.162	-0.061	-4.18	2.99	-10.05	1.68	-0.88	891	886
R Temporal pole	0.232	-0.054	-3.74	3.13	-9.88	2.39	-0.88	888	879
L Transverse temporal	0.172	0.060	4.91	3.59	-2.13	11.95	1.10	940	905
R Transverse temporal	0.578	0.024	1.45	2.60	-3.66	6.55	0.43	936	907

Results of cortical surface area analysis of HY stage 2 PD vs matched healthy controls. L – Left, R – Right, ROI – region of interest, Banks STS – Banks of the superior temporal sulcus. **Boldface** denotes significant results after Bonferroni correction

Supplementary Table 4g: Cortical surface area results - HY3 PD vs controls

ROI Surface Area	*p*	*d*	*b*	SE	Lower CI	Upper CI	% diff	NPD	NHC
L Banks STS	0.017	-0.177	-31.00	12.91	-56.36	-5.64	-3.14	228	453
R Banks STS	0.048	-0.144	-22.30	11.25	-44.39	-0.20	-2.45	238	482
L Caudal anterior cingulate	0.170	-0.103	-14.59	10.61	-35.43	6.25	-2.34	256	497
R Caudal anterior cingulate	0.417	-0.059	-10.04	12.37	-34.32	14.25	-1.34	256	499
L Caudal middle frontal	0.011	-0.184	-69.33	27.20	-122.73	-15.93	-3.13	257	499
R Caudal middle frontal	0.058	-0.142	-53.97	28.43	-109.78	1.84	-2.61	255	495
L Cuneus	0.003	-0.234	-50.80	17.09	-84.35	-17.24	-3.53	223	471
**R Cuneus**	**<0.001**	**-0.271**	**-62.06**	**17.61**	**-96.64**	**-27.49**	**-4.16**	**228**	**448**
L Entorhinal	0.232	-0.090	-8.36	6.98	-22.06	5.35	-2.01	256	497
R Entorhinal	0.442	-0.059	-4.95	6.43	-17.58	7.67	-1.38	243	486
**L Frontal pole**	**<0.001**	**-0.291**	**-12.22**	**2.72**	**-17.56**	**-6.88**	**-5.82**	**258**	**500**
R Frontal pole	0.233	-0.079	-4.27	3.57	-11.28	2.75	-1.55	257	500
**L Fusiform**	**<0.001**	**-0.260**	**-105.68**	**29.16**	**-162.94**	**-48.43**	**-3.40**	**252**	**499**
R Fusiform	0.004	-0.207	-86.45	29.73	-144.82	-28.08	-2.85	252	499
L Inferior parietal	0.005	-0.208	-132.53	47.16	-225.13	-39.93	-2.99	247	482
R Inferior parietal	0.007	-0.197	-149.07	55.23	-257.51	-40.63	-2.83	250	492
**L Inferior temporal**	**<0.001**	**-0.260**	**-127.58**	**36.53**	**-199.30**	**-55.86**	**-3.96**	**232**	**478**
**R Inferior temporal**	**<0.001**	**-0.270**	**-122.26**	**33.71**	**-188.44**	**-56.07**	**-4.00**	**240**	**480**
L Insula	0.385	-0.060	-16.77	19.28	-54.62	21.08	-0.76	253	495
R Insula	0.024	-0.160	-49.43	21.87	-92.37	-6.49	-2.16	254	490
L Isthmus cingulate	0.682	-0.029	-5.33	13.02	-30.89	20.23	-0.53	258	499
R Isthmus cingulate	0.531	0.042	7.08	11.29	-15.08	29.24	0.76	255	500
**L Lateral occipital**	**<0.001**	**-0.324**	**-211.56**	**46.95**	**-303.75**	**-119.38**	**-4.49**	**246**	**489**
**R Lateral occipital**	**<0.001**	**-0.286**	**-192.27**	**49.49**	**-289.44**	**-95.09**	**-4.18**	**250**	**490**
L Lateral orbitofrontal	0.028	-0.160	-47.78	21.71	-90.40	-5.16	-1.89	258	500
R Lateral orbitofrontal	0.100	-0.118	-39.07	23.74	-85.67	7.53	-1.58	256	500
**L Lingual**	**<0.001**	**-0.253**	**-116.79**	**33.75**	**-183.06**	**-50.52**	**-3.98**	**241**	**488**
**R Lingual**	**<0.001**	**-0.294**	**-133.57**	**33.37**	**-199.09**	**-68.05**	**-4.48**	**243**	**473**
L Medial orbitofrontal	0.002	-0.222	-52.67	17.06	-86.17	-19.17	-2.89	254	493
R Medial orbitofrontal	0.012	-0.182	-39.49	15.67	-70.26	-8.73	-2.19	254	491
**L Middle temporal**	**<0.001**	**-0.300**	**-132.48**	**32.79**	**-196.86**	**-68.09**	**-4.43**	**229**	**464**
**R Middle temporal**	**<0.001**	**-0.353**	**-154.36**	**31.01**	**-215.25**	**-93.47**	**-4.72**	**249**	**478**
L Paracentral	0.567	0.042	8.65	15.11	-21.01	38.31	0.65	253	497
R Paracentral	0.968	-0.003	-0.68	17.09	-34.22	32.87	-0.04	252	499
L Parahippocampal	0.010	-0.190	-22.07	8.57	-38.88	-5.25	-3.28	257	499
R Parahippocampal	0.076	-0.130	-12.76	7.17	-26.84	1.32	-1.96	255	500

L Pars opercularis	0.021	-0.172	-44.80	19.30	-82.70	-6.90	-2.83	257	500
R Pars opercularis	0.156	-0.104	-23.15	16.30	-55.17	8.86	-1.74	257	499
L Pars orbitalis	0.017	-0.171	-15.16	6.33	-27.60	-2.73	-2.45	258	499
R Pars orbitalis	0.007	-0.195	-20.91	7.77	-36.16	-5.66	-2.80	256	499
L Pars triangularis	0.358	-0.068	-13.85	15.05	-43.40	15.71	-1.12	258	500
R Pars triangularis	0.261	-0.083	-20.29	18.05	-55.73	15.15	-1.43	257	499
L Pericalcarine	0.037	-0.166	-47.25	22.61	-91.64	-2.86	-3.53	217	467
R Pericalcarine	0.054	-0.147	-41.53	21.53	-83.81	0.75	-2.85	220	440
L Postcentral	0.009	-0.197	-89.95	34.16	-157.02	-22.87	-2.18	246	482
R Postcentral	0.029	-0.165	-76.55	34.94	-145.15	-7.96	-1.92	243	483
L Posterior cingulate	0.085	-0.125	-23.31	13.52	-49.85	3.22	-2.06	256	498
R Posterior cingulate	0.226	-0.085	-17.05	14.06	-44.65	10.56	-1.47	257	499
L Precentral	0.096	-0.123	-68.34	40.98	-148.80	12.12	-1.41	245	480
R Precentral	0.037	-0.155	-80.48	38.49	-156.06	-4.91	-1.66	246	480
**L Precuneus**	**<0.001**	**-0.284**	**-135.45**	**33.52**	**-201.27**	**-69.64**	**-3.67**	**256**	**499**
R Precuneus	0.001	-0.228	-108.97	33.77	-175.27	-42.67	-2.84	256	499
L Rostral anterior cingulate	0.571	0.042	7.17	12.65	-17.67	32.00	0.87	255	482
R Rostral anterior cingulate	0.163	-0.096	-15.02	10.76	-36.14	6.11	-2.24	253	489
**L Rostral middle frontal**	**<0.001**	**-0.256**	**-192.90**	**54.08**	**-299.08**	**-86.72**	**-3.51**	**255**	**500**
**R Rostral middle frontal**	**<0.001**	**-0.248**	**-194.43**	**55.56**	**-303.51**	**-85.36**	**-3.45**	**257**	**498**
L Superior frontal	0.026	-0.159	-133.08	59.62	-250.14	-16.01	-1.91	251	488
R Superior frontal	0.008	-0.193	-158.20	59.10	-274.22	-42.17	-2.35	250	494
**L Superior parietal**	**<0.001**	**-0.306**	**-199.57**	**49.33**	**-296.43**	**-102.71**	**-3.77**	**242**	**495**
**R Superior parietal**	**<0.001**	**-0.257**	**-165.69**	**48.27**	**-260.46**	**-70.92**	**-3.11**	**245**	**495**
L Superior temporal	0.049	-0.154	-68.84	34.84	-137.26	-0.42	-1.86	210	451
R Superior temporal	0.022	-0.170	-64.08	27.96	-118.99	-9.18	-1.84	237	478
L Supramarginal	0.013	-0.188	-110.99	44.78	-198.92	-23.06	-2.92	243	476
R Supramarginal	0.098	-0.124	-65.19	39.35	-142.45	12.07	-1.82	247	491
L Temporal pole	0.025	-0.162	-11.49	5.11	-21.53	-1.45	-2.42	237	494
R Temporal pole	0.141	-0.106	-7.78	5.28	-18.14	2.58	-1.82	240	487
L Transverse temporal	0.953	-0.004	-0.35	5.98	-12.09	11.39	-0.08	257	501
R Transverse temporal	0.248	-0.085	-4.77	4.13	-12.88	3.33	-1.43	257	501

Results of cortical surface area analysis of HY stage 3 PD vs matched healthy controls. L – Left, R – Right, ROI – region of interest, Banks STS – Banks of the superior temporal sulcus. **Boldface** denotes significant results after Bonferroni correction

Supplementary Table 4h: Cortical surface area results - HY4&5 PD vs controls

ROI Surface Area	*p*	*d*	*b*	SE	Lower CI	Upper CI	% diff	NPD	NHC
L Banks STS	0.150	-0.162	-26.46	18.36	-62.57	9.65	-2.70	74	299
R Banks STS	0.105	-0.187	-26.81	16.51	-59.28	5.66	-3.01	79	317
L Caudal anterior cingulate	0.451	-0.091	-11.67	15.48	-42.11	18.77	-1.92	82	325
R Caudal anterior cingulate	0.742	-0.038	-5.99	18.18	-41.74	29.77	-0.82	83	328
L Caudal middle frontal	0.543	-0.073	-23.20	38.09	-98.09	51.70	-1.06	82	327
R Caudal middle frontal	0.423	-0.095	-33.45	41.66	-115.36	48.47	-1.62	81	326
L Cuneus	0.090	-0.211	-44.79	26.35	-96.61	7.03	-3.13	76	306
R Cuneus	0.007	-0.334	-69.19	25.43	-119.22	-19.15	-4.68	74	290
L Entorhinal	0.320	-0.122	-10.17	10.21	-30.25	9.90	-2.46	83	324
R Entorhinal	0.757	-0.036	-2.96	9.55	-21.74	15.83	-0.86	83	316

L Frontal pole	0.085	-0.179	-6.97	4.04	-14.91	0.97	-3.34	83	325
R Frontal pole	0.507	0.074	3.51	5.28	-6.88	13.89	1.29	83	326
L Fusiform	0.028	-0.251	-90.07	40.88	-170.46	-9.69	-2.94	83	328
R Fusiform	<0.001	-0.379	-139.17	41.83	-221.43	-56.91	-4.64	83	329
L Inferior parietal	0.006	-0.320	-184.95	66.82	-316.36	-53.54	-4.17	81	312
R Inferior parietal	0.030	-0.249	-158.79	72.92	-302.19	-15.39	-3.04	83	321
**L Inferior temporal**	**<0.001**	**-0.405**	**-181.61**	**50.55**	**-281.03**	**-82.19**	**-5.73**	**81**	**315**
R Inferior temporal	0.229	-0.137	-54.20	45.01	-142.71	34.31	-1.81	82	323
L Insula	0.174	-0.146	-35.31	25.95	-86.33	15.72	-1.63	83	326
R Insula	0.002	-0.344	-93.40	30.47	-153.31	-33.48	-4.18	83	326
L Isthmus cingulate	0.901	0.015	2.19	17.65	-32.51	36.89	0.22	82	327
R Isthmus cingulate	0.231	0.137	20.15	16.80	-12.88	53.18	2.18	83	329
**L Lateral occipital**	**<0.001**	**-0.423**	**-257.06**	**70.62**	**-395.94**	**-118.17**	**-5.50**	**78**	**319**
R Lateral occipital	0.032	-0.249	-154.31	71.68	-295.28	-13.33	-3.38	80	321
L Lateral orbitofrontal	0.006	-0.320	-83.00	30.05	-142.09	-23.90	-3.34	83	328
R Lateral orbitofrontal	0.066	-0.211	-61.06	33.15	-126.24	4.12	-2.51	82	329
L Lingual	0.002	-0.366	-153.06	50.03	-251.44	-54.68	-5.20	78	325
R Lingual	0.009	-0.305	-135.01	51.41	-236.12	-33.90	-4.56	78	315
L Medial orbitofrontal	0.002	-0.351	-73.57	23.73	-120.23	-26.91	-4.10	83	323
R Medial orbitofrontal	0.419	-0.090	-17.94	22.17	-61.54	25.67	-1.01	83	325
L Middle temporal	0.029	-0.257	-102.39	46.75	-194.34	-10.44	-3.49	77	305
R Middle temporal	0.001	-0.375	-145.50	44.08	-232.19	-58.81	-4.50	82	318
L Paracentral	0.429	-0.095	-17.35	21.92	-60.44	25.75	-1.32	83	326
R Paracentral	0.484	-0.072	-15.49	22.09	-58.92	27.95	-1.04	83	326
L Parahippocampal	0.188	-0.151	-16.65	12.63	-41.47	8.18	-2.46	83	327
R Parahippocampal	0.724	-0.040	-3.29	9.29	-21.55	14.97	-0.51	83	328
L Pars opercularis	0.832	-0.026	-6.19	29.14	-63.49	51.11	-0.40	83	327
R Pars opercularis	0.029	-0.253	-54.52	24.89	-103.47	-5.57	-4.05	83	328
L Pars orbitalis	0.011	-0.297	-22.91	8.94	-40.49	-5.33	-3.77	83	327
R Pars orbitalis	0.009	-0.301	-29.71	11.39	-52.11	-7.30	-4.03	83	328
L Pars triangularis	0.086	-0.201	-39.29	22.85	-84.23	5.65	-3.24	82	328
R Pars triangularis	0.379	-0.105	-21.45	24.37	-69.38	26.47	-1.53	83	329
L Pericalcarine	0.031	-0.267	-67.07	31.00	-128.04	-6.10	-5.01	75	308
R Pericalcarine	0.003	-0.369	-97.01	32.61	-161.17	-32.84	-6.61	71	287
L Postcentral	0.281	-0.124	-54.76	50.71	-154.50	44.98	-1.33	81	309
R Postcentral	0.048	-0.230	-96.98	48.89	-193.12	-0.84	-2.45	81	314
L Posterior cingulate	0.212	-0.145	-24.99	20.01	-64.34	14.35	-2.27	83	328
R Posterior cingulate	0.037	-0.239	-42.40	20.21	-82.14	-2.67	-3.72	81	328
L Precentral	0.315	-0.117	-53.85	53.53	-159.14	51.44	-1.12	80	310
R Precentral	0.350	-0.106	-49.91	53.38	-154.90	55.08	-1.03	81	313
**L Precuneus**	**<0.001**	**-0.424**	**-186.28**	**48.56**	**-281.76**	**-90.81**	**-5.10**	**83**	**327**
**R Precuneus**	**<0.001**	**-0.400**	**-180.86**	**50.77**	**-280.69**	**-81.03**	**-4.79**	**83**	**328**
L Rostral anterior cingulate	0.451	-0.089	-13.14	17.41	-47.38	21.10	-1.61	83	318
R Rostral anterior cingulate	0.592	-0.059	-8.71	16.23	-40.63	23.21	-1.33	83	324
L Rostral middle frontal	0.010	-0.292	-189.61	73.58	-334.30	-44.91	-3.48	83	328
R Rostral middle frontal	0.106	-0.182	-126.62	78.10	-280.20	26.96	-2.28	83	324
L Superior frontal	0.007	-0.299	-216.21	80.30	-374.12	-58.31	-3.15	81	319
R Superior frontal	0.009	-0.297	-211.42	81.05	-370.81	-52.02	-3.19	82	319
L Superior parietal	0.011	-0.307	-186.21	72.72	-329.22	-43.19	-3.55	83	317

R Superior parietal	0.016	-0.279	-161.48	66.74	-292.73	-30.23	-3.09	83	321
L Superior temporal	0.026	-0.257	-109.15	48.84	-205.22	-13.08	-2.99	76	294
R Superior temporal	0.001	-0.361	-124.74	38.63	-200.72	-48.76	-3.59	80	311
L Supramarginal	0.103	-0.192	-97.91	59.85	-215.62	19.81	-2.66	83	311
R Supramarginal	0.039	-0.239	-108.40	52.40	-211.45	-5.35	-3.08	83	320
L Temporal pole	0.293	-0.126	-7.90	7.51	-22.68	6.87	-1.68	80	324
R Temporal pole	0.798	-0.029	-2.04	7.96	-17.69	13.61	-0.48	82	316
L Transverse temporal	0.726	-0.040	-3.16	9.00	-20.85	14.54	-0.70	83	328
R Transverse temporal	0.134	-0.174	-9.46	6.29	-21.84	2.91	-2.86	83	329

Results of cortical surface area analysis of HY stage 4&5 PD vs matched healthy controls. L – Left, R – Right, ROI – region of interest, Banks STS – Banks of the superior temporal sulcus. **Boldface** denotes significant results after Bonferroni correction

Supplementary Table 4i: Subcortical volume results - HY1 PD vs controls

ROI Subcortical	*p*	*d*	*b*	SE	Lower CI	Upper CI	% diff	NPD	NHC
L Amygdala	0.199	-0.064	-17.14	13.35	-43.33	9.06	-1.15	428	834
R Amygdala	0.901	0.006	1.68	13.5	-24.79	28.16	0.11	430	827
L Caudate nucleus	0.306	0.054	25.92	25.3	-23.72	75.55	0.76	430	835
R Caudate nucleus	0.157	0.072	36.6	25.85	-14.12	87.33	1.05	432	844
L Globus pallidus	0.396	-0.041	-12.66	14.91	-41.91	16.59	-0.86	418	762
R Globus pallidus	0.188	-0.068	-18.17	13.8	-45.25	8.9	-1.21	423	823
L Hippocampus	0.185	0.069	36.79	27.76	-17.67	91.24	0.93	428	819
R Hippocampus	0.225	0.062	34.19	28.13	-21.01	89.38	0.84	429	831
L Lateral ventricle	0.220	0.069	494	402.53	-295.73	1283.73	4.22	434	840
R Lateral ventricle	0.055	0.109	725.13	376.92	-14.35	1464.62	6.74	433	841
L Nucleus accumbens	0.907	0.006	0.83	7.08	-13.06	14.72	0.17	434	832
R Nucleus accumbens	0.916	0.005	0.71	6.67	-12.39	13.8	0.14	432	824
L Putamen	0.146	-0.077	-59.36	40.79	-139.39	20.68	-1.17	422	763
R Putamen	0.157	-0.074	-55.82	39.41	-133.14	21.49	-1.15	424	814
**L Thalamus**	**0.002**	**0.151**	**153.72**	**49.27**	**57.04**	**250.39**	**2.15**	**417**	**809**
R Thalamus	0.094	0.086	68.45	40.83	-11.66	148.56	1.03	420	815

Results of subcortical volume analysis of HY stage 1 PD vs matched healthy controls. L – Left, R – Right, ROI – region of interest. **Boldface** denotes significant results after Bonferroni correction

Supplementary Table 4j: Subcortical volume results - HY2 PD vs controls

ROI Subcortical	*p*	*d*	*b*	SE	Lower CI	Upper CI	% diff	NPD	NHC
**L Amygdala**	**<0.001**	**-0.159**	**-44.62**	**11.07**	**-66.34**	**-22.91**	**-2.99**	**927**	**894**
**R Amygdala**	**0.002**	**-0.119**	**-36.37**	**11.66**	**-59.24**	**-13.51**	**-2.28**	**924**	**891**
L Caudate nucleus	0.006	-0.113	-61.99	22.67	-106.45	-17.53	-1.80	929	895
R Caudate nucleus	0.066	-0.075	-41.79	22.71	-86.33	2.74	-1.20	925	904
L Globus pallidus	0.064	-0.074	-23.72	12.81	-48.84	1.39	-1.60	893	812
R Globus pallidus	0.429	-0.031	-9.04	11.44	-31.48	13.39	-0.61	919	880
L Hippocampus	0.225	-0.049	-29.14	23.99	-76.2	17.91	-0.74	915	882
R Hippocampus	0.305	-0.040	-24.39	23.8	-71.07	22.28	-0.60	922	888
L Lateral ventricle	0.165	0.062	489.92	352.6	-201.64	1181.47	3.69	937	902

R Lateral ventricle	0.089	0.076	543.48	319.86	-83.85	1170.81	4.47	937	903
L Nucleus accumbens	0.124	-0.062	-8.85	5.75	-20.13	2.43	-1.85	926	895
R Nucleus accumbens	0.138	-0.059	-8	5.39	-18.56	2.57	-1.62	919	886
**L Putamen**	**<0.001**	**-0.152**	**-123.03**	**33.36**	**-188.46**	**-57.61**	**-2.45**	**904**	**823**
**R Putamen**	**<0.001**	**-0.152**	**-119.63**	**31.18**	**-180.78**	**-58.48**	**-2.49**	**926**	**866**
L Thalamus	0.045	0.075	82.32	41.06	1.79	162.86	1.15	892	865
R Thalamus	0.188	0.052	46.04	34.93	-22.46	114.54	0.69	911	878

Results of subcortical volume analysis of HY stage 2 PD vs matched healthy controls. L – Left, R – Right, ROI – region of interest. **Boldface** denotes significant results after Bonferroni correction

Supplementary Table 4k: Subcortical volume results - HY3 PD vs controls

ROI Subcortical	*p*	*d*	*b*	SE	Lower CI	Upper CI	% diff	NPD	NHC
**L Amygdala**	**<0.001**	**-0.441**	**-126.03**	**19.43**	**-164.18**	**-87.88**	**-8.63**	**256**	**492**
**R Amygdala**	**<0.001**	**-0.316**	**-98.12**	**20.91**	**-139.18**	**-57.06**	**-6.28**	**257**	**491**
**L Caudate nucleus**	**0.001**	**-0.221**	**-128.51**	**39.79**	**-206.64**	**-50.39**	**-3.72**	**251**	**494**
R Caudate nucleus	0.010	-0.170	-103.46	39.86	-181.73	-25.20	-2.97	255	500
L Globus pallidus	0.008	-0.169	-59.24	22.43	-103.28	-15.19	-3.97	239	445
R Globus pallidus	0.020	-0.152	-48.34	20.66	-88.91	-7.77	-3.22	249	481
**L Hippocampus**	**<0.001**	**-0.240**	**-133.03**	**40.25**	**-212.07**	**-54.00**	**-3.43**	**249**	**477**
**R Hippocampus**	**<0.001**	**-0.283**	**-170.77**	**42.26**	**-253.74**	**-87.80**	**-4.27**	**255**	**487**
L Lateral ventricle	0.032	0.158	1426.00	664.17	122.04	2729.96	9.70	257	498
R Lateral ventricle	0.023	0.166	1356.27	597.40	183.40	2529.14	10.06	257	499
L Nucleus accumbens	0.023	-0.150	-23.16	10.13	-43.05	-3.27	-4.98	256	492
R Nucleus accumbens	0.013	-0.157	-23.31	9.38	-41.73	-4.89	-4.83	251	490
**L Putamen**	**<0.001**	**-0.267**	**-230.11**	**55.72**	**-339.53**	**-120.69**	**-4.67**	**242**	**450**
**R Putamen**	**<0.001**	**-0.239**	**-195.14**	**51.77**	**-296.78**	**-93.50**	**-4.16**	**249**	**473**
L Thalamus	0.980	0.002	1.73	69.11	-133.96	137.42	0.02	249	478
R Thalamus	0.185	-0.088	-80.69	60.84	-200.16	38.77	-1.23	250	487

Results of subcortical volume analysis of HY stage 3 PD vs matched healthy controls. L – Left, R – Right, ROI – region of interest. **Boldface** denotes significant results after Bonferroni correction

Supplementary Table 4l: Subcortical volume results - HY4&5 PD vs controls

ROI Subcortical	*p*	*d*	*b*	SE	Lower CI	Upper CI	% diff	NPD	NHC
**L Amygdala**	**<0.001**	**-0.640**	**-163.65**	**27.08**	**-216.90**	**-110.39**	**-11.61**	**83**	**323**
**R Amygdala**	**<0.001**	**-0.554**	**-154.92**	**28.86**	**-211.68**	**-98.16**	**-9.98**	**83**	**322**
**L Caudate nucleus**	**0.002**	**-0.329**	**-177.27**	**56.29**	**-287.96**	**-66.58**	**-5.20**	**82**	**327**
R Caudate nucleus	0.032	-0.223	-124.57	57.82	-238.27	-10.87	-3.62	81	329
**L Globus pallidus**	**0.002**	**-0.317**	**-102.32**	**32.14**	**-165.54**	**-39.09**	**-6.97**	**83**	**292**
R Globus pallidus	0.011	-0.261	-83.00	32.28	-146.49	-19.51	-5.67	82	314
**L Hippocampus**	**<0.001**	**-0.546**	**-257.72**	**55.09**	**-366.06**	**-149.37**	**-6.82**	**81**	**318**
**R Hippocampus**	**<0.001**	**-0.616**	**-318.63**	**58.09**	**-432.87**	**-204.39**	**-8.11**	**82**	**318**
**L Lateral ventricle**	**0.002**	**0.357**	**2761.46**	**885.08**	**1021.08**	**4501.84**	**18.43**	**83**	**329**
**R Lateral ventricle**	**<0.001**	**0.523**	**3724.85**	**814.85**	**2122.56**	**5327.15**	**27.80**	**83**	**328**
L Nucleus accumbens	0.007	-0.294	-39.29	14.46	-67.71	-10.86	-8.58	82	324
**R Nucleus accumbens**	**<0.001**	**-0.473**	**-59.94**	**13.14**	**-85.79**	**-34.10**	**-12.46**	**81**	**320**

**L Putamen**	**<0.001**	**-0.423**	**-314.82**	**78.06**	**-468.37**	**-161.27**	**-6.43**	**80**	**296**
**R Putamen**	**<0.001**	**-0.473**	**-350.88**	**77.40**	**-503.10**	**-198.66**	**-7.47**	**82**	**311**
L Thalamus	0.027	-0.223	-203.27	91.70	-383.62	-22.92	-2.99	81	310
R Thalamus	0.029	-0.220	-178.29	81.16	-337.90	-18.68	-2.77	82	317

Results of subcortical volume analysis of HY stages 4&5 PD vs matched healthy controls. L – Left, R – Right, ROI – region of interest. **Boldface** denotes significant results after Bonferroni correction

Supplementary table 5a: Results of regression analysis of MoCA score and cortical thickness

ROI Thickness	*p*	*r*	b	SE	Lower CI	Upper CI	NPD
L Banks STS	0.002	0.082	0.006	0.002	0.002	0.010	940
R Banks STS	0.002	0.077	0.006	0.002	0.002	0.010	1005
L Caudal anterior cingulate	0.437	0.021	0.002	0.003	-0.003	0.008	1026
R Caudal anterior cingulate	0.558	0.015	0.002	0.003	-0.004	0.007	1025
**L Caudal middle frontal**	**<0.001**	**0.119**	**0.007**	**0.002**	0.004	0.011	**1034**
**R Caudal middle frontal**	**<0.001**	**0.104**	**0.006**	**0.002**	0.003	0.009	**1034**
L Cuneus	0.158	0.039	0.002	0.002	-0.001	0.005	939
R Cuneus	0.414	0.022	0.001	0.002	-0.002	0.004	929
L Entorhinal	0.042	0.056	0.007	0.003	<0.001	0.013	1017
R Entorhinal	0.016	0.067	0.009	0.004	0.002	0.016	1011
**L Frontal pole**	**<0.001**	**0.107**	**0.011**	**0.003**	0.005	0.016	**1031**
R Frontal pole	0.008	0.074	0.007	0.003	0.002	0.013	1030
**L Fusiform**	**<0.001**	**0.139**	**0.009**	**0.002**	0.006	0.013	**1012**
**R Fusiform**	**<0.001**	**0.140**	**0.010**	**0.002**	0.006	0.013	**1019**
L Inferior parietal	0.004	0.073	0.004	0.002	0.001	0.007	1006
**R Inferior parietal**	**<0.001**	**0.092**	**0.006**	**0.002**	0.003	0.009	**1004**
**L Inferior temporal**	**<0.001**	**0.111**	**0.008**	**0.002**	0.004	0.011	**991**
**R Inferior temporal**	**<0.001**	**0.103**	**0.007**	**0.002**	0.004	0.010	**1007**
**L Insula**	**<0.001**	**0.116**	**0.008**	**0.002**	0.004	0.011	**1031**
**R Insula**	**<0.001**	**0.124**	**0.009**	**0.002**	0.005	0.013	**1028**
L Isthmus cingulate	0.028	0.060	0.004	0.002	<0.001	0.008	1031
R Isthmus cingulate	0.007	0.074	0.006	0.002	0.002	0.010	1031
L Lateral occipital	0.515	0.017	0.001	0.001	-0.002	0.004	1006
R Lateral occipital	0.012	0.064	0.004	0.001	0.001	0.006	1007
L Lateral orbitofrontal	0.007	0.064	0.005	0.002	0.001	0.008	1025
R Lateral orbitofrontal	<0.001	0.082	0.006	0.002	0.002	0.009	1026
L Lingual	0.003	0.082	0.004	0.001	0.001	0.007	998
R Lingual	0.006	0.074	0.004	0.001	0.001	0.007	981
L Medial orbitofrontal	0.001	0.085	0.006	0.002	0.002	0.009	1019
R Medial orbitofrontal	0.007	0.065	0.005	0.002	0.001	0.009	1022
**L Middle temporal**	**<0.001**	**0.133**	**0.010**	**0.002**	0.006	0.013	**943**
**R Middle temporal**	**<0.001**	**0.101**	**0.008**	**0.002**	0.004	0.011	**1005**
L Paracentral	0.002	0.082	0.006	0.002	0.002	0.009	1028
R Paracentral	0.002	0.084	0.005	0.002	0.002	0.009	1026
L Parahippocampal	0.180	0.037	0.004	0.003	-0.002	0.010	1030
R Parahippocampal	0.003	0.078	0.008	0.003	0.003	0.013	1030
**L Pars opercularis**	**<0.001**	**0.096**	**0.006**	**0.002**	0.003	0.009	**1025**
R Pars opercularis	0.002	0.081	0.005	0.002	0.002	0.008	1023
L Pars orbitalis	0.084	0.046	0.004	0.002	<0.001	0.008	1025
R Pars orbitalis	0.031	0.055	0.004	0.002	<0.001	0.008	1024

**L Pars triangularis**	**<0.001**	**0.135**	**0.009**	**0.002**	0.006	0.012	**1023**
**R Pars triangularis**	**<0.001**	**0.099**	**0.006**	**0.002**	0.003	0.009	**1022**
L Pericalcarine	0.274	0.030	0.002	0.002	-0.001	0.005	940
R Pericalcarine	0.295	0.029	0.002	0.002	-0.001	0.005	912
**L Postcentral**	**<0.001**	**0.097**	**0.005**	**0.001**	0.003	0.008	**995**
R Postcentral	0.002	0.079	0.004	0.001	0.002	0.007	996
L Posterior cingulate	0.002	0.077	0.005	0.002	0.002	0.009	1032
R Posterior cingulate	0.005	0.073	0.005	0.002	0.002	0.008	1031
**L Precentral**	**<0.001**	**0.125**	**0.008**	**0.002**	0.005	0.012	**1018**
**R Precentral**	**<0.001**	**0.118**	**0.008**	**0.002**	0.004	0.012	**1021**
**L Precuneus**	**<0.001**	**0.087**	**0.005**	**0.001**	0.002	0.008	**1033**
**R Precuneus**	**<0.001**	**0.102**	**0.006**	**0.002**	0.003	0.009	**1032**
**L Rostral anterior cingulate**	**<0.001**	**0.093**	**0.008**	**0.002**	0.004	0.013	**1022**
R Rostral anterior cingulate	0.355	0.024	0.002	0.003	-0.003	0.008	1025
**L Rostral middle frontal**	**<0.001**	**0.114**	**0.007**	**0.001**	0.004	0.009	**1026**
R Rostral middle frontal	0.002	0.070	0.004	0.001	0.001	0.007	1026
**L Superior frontal**	**<0.001**	**0.100**	**0.006**	**0.002**	0.003	0.009	**1012**
**R Superior frontal**	**<0.001**	**0.105**	**0.006**	**0.002**	0.003	0.009	**1012**
L Superior parietal	0.006	0.070	0.004	0.001	0.001	0.007	1003
**R Superior parietal**	0.004	0.071	0.004	0.001	0.001	0.007	1017
**L Superior temporal**	**<0.001**	**0.120**	**0.008**	**0.002**	0.005	0.012	**904**
**R Superior temporal**	**<0.001**	**0.116**	**0.008**	**0.002**	0.005	0.012	**979**
L Supramarginal	0.002	0.080	0.005	0.002	0.002	0.008	997
R Supramarginal	0.003	0.077	0.005	0.002	0.002	0.008	1005
L Temporal pole	0.011	0.074	0.009	0.004	0.002	0.016	971
R Temporal pole	0.002	0.090	0.011	0.004	0.004	0.018	999
L Transverse temporal	0.002	0.086	0.007	0.002	0.002	0.012	1036
R Transverse temporal	**<0.001**	**0.099**	**0.009**	**0.002**	0.004	0.013	**1035**

Results of regression analysis of MoCA score and cortical thickness in PD sample. L – Left, R – Right, ROI – region of interest. Banks STS – Banks of the superior temporal sulcus. **Boldface** denotes significant results after Bonferroni correction.

Supplementary table 5b: Results of regression analysis of MoCA score and surface area

ROI Surface Area	*p*	*r*	b	SE	Lower CI	Upper CI	NPD
L Banks STS	0.004	0.092	4.64	1.595	1.51	7.77	940
R Banks STS	0.151	0.043	1.99	1.388	-0.73	4.72	1005
L Caudal anterior cingulate	0.045	0.060	2.82	1.405	0.06	5.57	1026
R Caudal anterior cingulate	0.049	0.058	3.12	1.585	0.01	6.23	1025
L Caudal middle frontal	0.074	0.054	6.06	3.389	**-0.59**	**12.71**	1035
R Caudal middle frontal	0.277	0.033	3.60	3.307	**-2.90**	**10.09**	1035
L Cuneus	0.796	0.008	0.52	1.997	-3.40	4.44	939
R Cuneus	0.519	0.021	1.38	2.139	-2.82	5.58	929
L Entorhinal	0.860	-0.005	-0.16	0.905	-1.94	1.62	1017
R Entorhinal	0.940	-0.002	-0.06	0.839	-1.71	1.58	1012
L Frontal pole	0.426	0.021	0.25	0.311	**-0.36**	**0.86**	1031
R Frontal pole	0.225	0.034	0.50	0.414	-0.31	1.32	1030
L Fusiform	0.660	0.013	1.51	3.428	**-5.22**	**8.23**	1013
R Fusiform	0.485	0.021	2.30	3.290	**-4.16**	**8.75**	1018
L Inferior parietal	0.085	0.051	9.07	5.261	-1.26	19.39	1006

**R Inferior parietal**	**<0.001**	**0.122**	**23.83**	**5.904**	**12.25**	**35.42**	**1004**
L Inferior temporal	0.450	0.023	3.17	4.201	**-5.07**	**11.42**	991
R Inferior temporal	0.262	0.034	4.16	3.709	**-3.12**	**11.44**	1007
L Insula	0.632	0.013	0.98	2.052	**-3.04**	**5.01**	1031
R Insula	0.519	-0.019	-1.59	2.462	**-6.42**	**3.24**	1028
L Isthmus cingulate	0.100	0.048	2.44	1.484	-0.47	5.35	1031
R Isthmus cingulate	0.646	-0.013	-0.65	1.415	-3.43	2.13	1031
L Lateral occipital	0.727	0.010	1.82	5.204	-8.40	12.03	1006
R Lateral occipital	0.021	0.068	13.04	5.636	1.98	24.10	1007
L Lateral orbitofrontal	0.204	0.039	3.14	2.472	-1.71	8.00	1026
R Lateral orbitofrontal	0.152	0.043	3.90	2.722	-1.44	9.24	1027
L Lingual	0.151	0.044	5.48	3.811	-2.00	12.96	998
R Lingual	0.325	0.030	3.77	3.828	-3.74	11.28	981
L Medial orbitofrontal	0.197	0.039	2.64	2.047	-1.38	6.66	1018
R Medial orbitofrontal	0.770	-0.009	-0.53	1.810	-4.08	3.02	1021
L Middle temporal	0.142	0.046	5.27	3.582	**-1.76**	**12.30**	943
R Middle temporal	0.085	0.052	6.08	3.532	**-0.85**	**13.01**	1005
L Paracentral	0.950	0.002	0.10	1.625	-3.09	3.29	1028
R Paracentral	0.661	-0.013	-0.92	2.091	-5.02	3.19	1026
L Parahippocampal	0.081	0.052	1.66	0.952	-0.20	3.53	1030
R Parahippocampal	0.089	0.051	1.59	0.935	-0.24	3.43	1030
**L Pars opercularis**	**<0.001**	**0.111**	**8.24**	2.231	**3.86**	**12.62**	**1025**
R Pars opercularis	0.186	0.040	2.56	1.933	-1.24	6.35	1023
L Pars orbitalis	0.012	0.073	1.90	0.755	0.41	3.38	1025
R Pars orbitalis	0.068	0.054	1.73	0.945	-0.13	3.58	1024
L Pars triangularis	0.048	0.061	3.49	1.762	**0.03**	**6.95**	1023
R Pars triangularis	0.307	0.031	2.25	2.201	**-2.07**	**6.57**	1022
L Pericalcarine	0.903	-0.004	-0.31	2.569	-5.36	4.73	940
R Pericalcarine	0.751	-0.010	-0.80	2.534	-5.78	4.17	912
L Postcentral	0.206	0.039	5.04	3.981	**-2.77**	**12.85**	995
R Postcentral	0.104	0.050	6.55	4.021	-1.34	14.44	996
L Posterior cingulate	0.147	0.043	2.97	2.044	-1.04	6.98	1032
R Posterior cingulate	0.263	0.032	1.83	1.631	-1.38	5.03	1031
L Precentral	0.423	0.024	3.52	4.396	**-5.10**	**12.15**	1018
R Precentral	0.014	0.074	11.18	4.523	**2.30**	**20.06**	1021
L Precuneus	0.004	0.086	11.31	3.896	**3.66**	**18.95**	1033
R Precuneus	0.089	0.050	7.11	4.180	**-1.09**	**15.31**	1032
L Rostral anterior cingulate	0.673	-0.013	-0.67	1.587	**-3.78**	**2.44**	1022
R Rostral anterior cingulate	0.377	0.025	1.14	1.287	-1.39	3.66	1025
L Rostral middle frontal	0.475	0.021	4.45	6.218	**-7.75**	**16.65**	1026
R Rostral middle frontal	0.281	0.031	6.85	6.347	-5.61	19.30	1026
L Superior frontal	0.994	<0.001	-0.05	6.706	**-13.21**	**13.11**	1013
R Superior frontal	0.166	0.041	9.54	6.881	**-3.96**	**23.04**	1012
L Superior parietal	0.001	0.096	17.64	5.523	6.80	28.47	1003
R Superior parietal	0.001	0.096	17.04	5.344	6.55	27.53	1017
L Superior temporal	0.169	0.043	5.43	3.950	**-2.32**	**13.19**	904
R Superior temporal	0.331	0.029	3.22	3.307	**-3.27**	**9.71**	979
L Supramarginal	0.002	0.093	16.45	5.329	5.99	26.91	997
R Supramarginal	0.396	0.026	3.99	4.706	-5.24	13.23	1005
L Temporal pole	0.405	0.025	0.53	0.635	-0.72	1.77	971
R Temporal pole	0.545	-0.018	-0.38	0.630	-1.62	0.85	999
L Transverse temporal	0.014	0.074	1.84	0.749	0.37	3.31	1036

R Transverse temporal	0.040	0.062	1.02	0.497	**0.05**	**2.00**	1035

Results of regression analysis of MoCA score and cortical surface area in PD sample. L – Left, R – Right, ROI – region of interest. Banks STS – Banks of the superior temporal sulcus. **Boldface** denotes significant results after Bonferroni correction.

Supplementary table 5c: Results of regression analysis of MoCA score and subcortical volume

ROI Subcortical	*p*	*r*	b	SE	Lower CI	Upper CI	NPD
**L Amygdala**	**<0.001**	**0.133**	**10.70**	**2.08**	6.61	14.79	**1047**
**R Amygdala**	**<0.001**	**0.110**	**9.41**	**2.19**	5.11	13.71	**1049**
L Caudate nucleus	0.242	0.033	5.61	4.79	-3.8	15.01	1044
R Caudate nucleus	0.414	0.023	4.00	4.9	-5.61	13.62	1045
L Globus pallidus	0.957	0.001	0.14	2.52	-4.81	5.08	1023
R Globus pallidus	0.705	0.009	0.87	2.29	-3.62	5.36	1040
**L Hippocampus**	**<0.001**	**0.109**	**16.84**	**4.23**	**8.54**	**25.14**	**1033**
**R Hippocampus**	**<0.001**	**0.118**	**19.91**	**4.45**	**11.18**	**28.65**	**1046**
**L Lateral ventricle**	**<0.001**	**-0.117**	**-289.26**	**73.37**	**-433.24**	**-145.29**	**1051**
**R Lateral ventricle**	**<0.001**	**-0.113**	**-262.86**	**68.38**	**-397.05**	**-128.67**	**1051**
L Nucleus accumbens	0.016	0.063	2.76	1.15	0.51	5.01	1048
R Nucleus accumbens	0.150	0.040	1.55	1.08	-0.56	3.67	1049
**L Putamen**	**0.002**	**0.081**	**18.36**	**6.05**	**6.49**	**30.23**	**1024**
R Putamen	0.045	0.056	11.78	5.86	0.28	23.28	1039
L Thalamus	0.060	0.050	13.83	7.35	-0.61	28.26	1009
R Thalamus	0.083	0.046	10.76	6.2	-1.4	22.92	1023

Results of regression analysis of MoCA score and subcortical volume in PD sample. L – Left, R – Right, ROI – region of interest. **Boldface** denotes significant results after Bonferroni correction.

Supplementary Table 6a: Cortical thickness results - PD (MoCA group) vs controls

ROI	*p*	*d*	*b*	SE	Lower CI	Upper CI	% diff	NPD	NHC
L Banks STS	0.034	-0.073	-0.026	0.012	-0.050	-0.002	-1.127	940	1081
R Banks STS	0.002	-0.102	-0.037	0.012	-0.061	-0.013	-1.525	1005	1129
L Caudal anterior cingulate	0.551	0.023	0.011	0.018	-0.025	0.047	0.421	1026	1169
R Caudal anterior cingulate	0.581	-0.021	-0.009	0.017	-0.042	0.023	-0.372	1025	1173
L Caudal middle frontal	0.034	-0.078	-0.022	0.010	-0.042	-0.002	-0.905	1034	1167
R Caudal middle frontal	0.159	-0.052	-0.014	0.010	-0.034	0.006	-0.597	1034	1167
L Cuneus	0.787	0.010	0.003	0.009	-0.016	0.021	0.145	939	1118
R Cuneus	0.342	-0.031	-0.009	0.010	-0.029	0.010	-0.519	929	1085
L Entorhinal	0.797	-0.010	-0.006	0.022	-0.049	0.038	-0.176	1017	1163
R Entorhinal	0.127	-0.049	-0.036	0.024	-0.082	0.010	-1.057	1011	1145
L Frontal pole	0.647	-0.018	-0.008	0.018	-0.043	0.027	-0.308	1031	1169
R Frontal pole	0.183	-0.051	-0.024	0.018	-0.058	0.011	-0.898	1030	1171
L Fusiform	0.006	-0.093	-0.029	0.010	-0.049	-0.008	-1.114	1012	1170
R Fusiform	<0.001	-0.101	-0.041	0.012	-0.065	-0.017	-1.552	1019	1172
L Inferior parietal	0.022	-0.081	-0.021	0.009	-0.039	-0.003	-0.928	1006	1128
**R Inferior parietal**	**<0.001**	**-0.119**	**-0.035**	**0.010**	**-0.053**	**-0.016**	**-1.480**	**1004**	**1153**
L Inferior temporal	0.003	-0.102	-0.031	0.011	-0.052	-0.011	-1.176	991	1110
R Inferior temporal	0.021	-0.075	-0.025	0.011	-0.046	-0.004	-0.925	1007	1129
L Insula	0.149	-0.055	-0.017	0.012	-0.041	0.006	-0.600	1031	1168

R Insula	0.282	-0.038	-0.014	0.013	-0.040	0.012	-0.491	1028	1163
L Isthmus cingulate	0.011	-0.096	-0.033	0.013	-0.058	-0.007	-1.383	1031	1173
R Isthmus cingulate	0.022	-0.083	-0.030	0.013	-0.055	-0.004	-1.277	1031	1175
L Lateral occipital	0.008	-0.098	-0.025	0.009	-0.043	-0.006	-1.168	1006	1151
R Lateral occipital	0.003	-0.105	-0.030	0.010	-0.049	-0.010	-1.370	1007	1157
L Lateral orbitofrontal	0.011	-0.087	-0.027	0.011	-0.048	-0.006	-1.068	1025	1168
R Lateral orbitofrontal	0.164	-0.044	-0.015	0.011	-0.036	0.006	-0.599	1026	1169
L Lingual	0.614	-0.018	-0.004	0.009	-0.021	0.013	-0.229	998	1158
R Lingual	0.420	-0.026	-0.007	0.009	-0.025	0.011	-0.373	981	1126
L Medial orbitofrontal	0.222	-0.045	-0.013	0.011	-0.034	0.008	-0.565	1019	1166
R Medial orbitofrontal	0.981	-0.001	<0.001	0.012	-0.023	0.022	-0.012	1022	1165
L Middle temporal	0.012	-0.089	-0.029	0.012	-0.052	-0.006	-1.096	943	1096
R Middle temporal	0.049	-0.063	-0.022	0.011	-0.044	<0.001	-0.818	1005	1136
L Paracentral	0.461	-0.026	-0.008	0.011	-0.031	0.014	-0.373	1028	1170
R Paracentral	0.403	-0.029	-0.010	0.011	-0.032	0.013	-0.417	1026	1173
L Parahippocampal	0.007	-0.104	-0.053	0.020	-0.092	-0.015	-2.036	1030	1169
**R Parahippocampal**	**<0.001**	**-0.156**	**-0.073**	**0.017**	**-0.106**	**-0.039**	**-2.801**	**1030**	**1173**
L Pars opercularis	0.741	-0.011	-0.003	0.010	-0.022	0.016	-0.135	1025	1174
R Pars opercularis	0.987	-0.001	<0.001	0.010	-0.021	0.020	-0.007	1023	1172
L Pars orbitalis	0.233	-0.044	-0.016	0.013	-0.042	0.010	-0.618	1025	1171
R Pars orbitalis	0.766	0.011	0.004	0.013	-0.022	0.030	0.155	1024	1172
L Pars triangularis	0.650	-0.016	-0.005	0.010	-0.024	0.015	-0.198	1023	1174
R Pars triangularis	0.648	0.016	0.004	0.010	-0.015	0.024	0.195	1022	1173
L Pericalcarine	0.897	-0.004	-0.001	0.009	-0.020	0.017	-0.078	940	1124
R Pericalcarine	0.565	0.017	0.006	0.010	-0.014	0.025	0.361	912	1070
L Postcentral	0.093	-0.057	-0.014	0.009	-0.031	0.002	-0.728	995	1130
R Postcentral	0.079	-0.057	-0.015	0.009	-0.032	0.002	-0.779	996	1134
L Posterior cingulate	0.026	-0.079	-0.025	0.011	-0.047	-0.003	-1.040	1032	1173
R Posterior cingulate	0.055	-0.065	-0.022	0.011	-0.044	<0.001	-0.915	1031	1174
L Precentral	0.039	-0.073	-0.023	0.011	-0.044	-0.001	-0.946	1018	1126
R Precentral	0.163	-0.050	-0.016	0.011	-0.038	0.006	-0.663	1021	1131
**L Precuneus**	**<0.001**	**-0.111**	**-0.031**	**0.009**	**-0.049**	**-0.013**	**-1.393**	**1033**	**1173**
**R Precuneus**	**<0.001**	**-0.132**	**-0.038**	**0.009**	**-0.056**	**-0.019**	**-1.673**	**1032**	**1174**
L Rostral anterior cingulate	0.159	-0.053	-0.022	0.016	-0.054	0.009	-0.821	1022	1164
R Rostral anterior cingulate	0.507	-0.023	-0.011	0.016	-0.042	0.021	-0.399	1025	1164
L Rostral middle frontal	0.441	-0.025	-0.007	0.009	-0.023	0.010	-0.293	1026	1170
R Rostral middle frontal	0.351	0.028	0.009	0.009	-0.009	0.026	0.384	1026	1168
L Superior frontal	0.019	-0.083	-0.023	0.010	-0.042	-0.004	-0.896	1012	1163
R Superior frontal	0.046	-0.067	-0.019	0.010	-0.038	<0.001	-0.748	1012	1163
L Superior parietal	0.117	-0.052	-0.014	0.009	-0.032	0.004	-0.684	1003	1150
R Superior parietal	0.001	-0.102	-0.030	0.009	-0.048	-0.012	-1.453	1017	1161
L Superior temporal	0.004	-0.101	-0.032	0.011	-0.054	-0.010	-1.245	904	1070
R Superior temporal	0.009	-0.078	-0.029	0.011	-0.050	-0.007	-1.098	979	1114
L Supramarginal	0.007	-0.096	-0.027	0.010	-0.046	-0.007	-1.124	997	1118
R Supramarginal	0.002	-0.105	-0.030	0.010	-0.049	-0.011	-1.252	1005	1148
L Temporal pole	0.157	-0.055	-0.031	0.022	-0.073	0.012	-0.862	971	1150
R Temporal pole	0.127	-0.052	-0.034	0.022	-0.078	0.010	-0.934	999	1142
L Transverse temporal	0.807	-0.009	-0.004	0.015	-0.032	0.025	-0.164	1036	1174
R Transverse temporal	0.043	-0.060	-0.032	0.016	-0.064	-0.001	-1.417	1035	1175

Results of cortical thickness analysis of PD patients with MoCA scores vs healthy controls. L – Left, R – Right, ROI – region of intereerest. Banks STS – Banks of the superior temporal sulcus. **Boldface** denotes significant results after Bonferroni correction

Supplementary Table 6b: Cortical surface area results - PD (MoCA group) vs controls

ROI	*p*	*d*	*b*	SE	Lower CI	Upper CI	% diff	NPD	NHC
L Banks STS	0.855	-0.008	-1.77	9.64	-20.68	17.14	-0.18	940	1069
R Banks STS	0.884	-0.006	-1.20	8.20	-17.27	14.88	-0.13	1005	1131
L Caudal anterior cingulate	0.109	0.066	13.06	8.14	-2.91	29.03	2.09	1026	1168
R Caudal anterior cingulate	0.569	-0.023	-5.38	9.43	-23.87	13.11	-0.71	1025	1172
L Caudal middle frontal	0.109	0.066	30.80	19.22	-6.90	68.49	1.39	1035	1165
R Caudal middle frontal	0.538	0.026	11.94	19.38	-26.05	49.94	0.58	1035	1166
L Cuneus	0.396	-0.036	-10.13	11.93	-33.53	13.27	-0.70	939	1110
R Cuneus	0.014	-0.105	-30.82	12.50	-55.33	-6.30	-2.05	929	1063
L Entorhinal	0.195	0.054	6.77	5.22	-3.46	16.99	1.63	1017	1162
R Entorhinal	0.593	0.022	2.59	4.85	-6.93	12.11	0.73	1012	1143
L Frontal pole	0.444	-0.028	-1.54	2.02	-5.50	2.41	-0.73	1031	1169
R Frontal pole	0.599	0.020	1.37	2.61	-3.74	6.48	0.50	1030	1171
L Fusiform	0.599	0.021	11.06	21.02	-30.17	52.29	0.35	1013	1170
R Fusiform	0.110	-0.063	-32.71	20.44	-72.80	7.39	-1.07	1018	1171
L Inferior parietal	0.485	-0.028	-22.44	32.16	-85.51	40.63	-0.50	1006	1127
R Inferior parietal	0.699	0.016	14.34	37.05	-58.31	87.00	0.27	1004	1153
L Inferior temporal	0.453	-0.030	-19.03	25.39	-68.82	30.75	-0.59	991	1107
R Inferior temporal	0.627	-0.020	-11.28	23.18	-56.73	34.18	-0.37	1007	1128
L Insula	0.044	0.075	26.28	13.05	0.68	51.87	1.20	1031	1160
R Insula	0.938	-0.003	-1.19	15.23	-31.06	28.68	-0.05	1028	1156
L Isthmus cingulate	0.170	0.055	12.60	9.19	-5.42	30.62	1.26	1031	1171
R Isthmus cingulate	0.728	0.014	2.97	8.56	-13.81	19.76	0.32	1031	1173
L Lateral occipital	0.114	-0.063	-51.56	32.62	-115.54	12.43	-1.09	1006	1151
R Lateral occipital	0.425	-0.032	-27.21	34.11	-94.10	39.68	-0.59	1007	1155
L Lateral orbitofrontal	0.168	0.056	20.85	15.11	-8.79	50.49	0.82	1026	1168
R Lateral orbitofrontal	0.020	0.093	38.82	16.73	6.01	71.63	1.57	1027	1170
L Lingual	0.463	-0.030	-17.56	23.89	-64.41	29.30	-0.59	998	1155
R Lingual	0.200	-0.052	-30.90	24.12	-78.19	16.40	-1.02	981	1118
L Medial orbitofrontal	0.360	0.037	11.45	12.51	-13.07	35.98	0.63	1018	1160
R Medial orbitofrontal	0.361	0.036	10.10	11.06	-11.60	31.80	0.56	1021	1160
L Middle temporal	0.044	-0.083	-44.83	22.21	-88.38	-1.28	-1.49	943	1087
R Middle temporal	0.846	-0.008	-4.22	21.82	-47.01	38.56	-0.13	1005	1136
L Paracentral	0.010	0.106	25.70	10.02	6.05	45.36	1.93	1028	1169
R Paracentral	0.506	0.027	7.95	11.94	-15.47	31.38	0.53	1026	1172
L Parahippocampal	0.406	0.034	4.79	5.76	-6.50	16.08	0.71	1030	1170
R Parahippocampal	0.065	0.075	9.90	5.35	-0.60	20.40	1.51	1030	1173
L Pars opercularis	0.819	0.009	3.15	13.72	-23.76	30.06	0.20	1025	1172
R Pars opercularis	0.272	-0.045	-12.98	11.82	-36.16	10.20	-0.96	1023	1168
L Pars orbitalis	0.145	0.058	6.60	4.53	-2.29	15.50	1.07	1025	1171
R Pars orbitalis	0.053	0.078	11.05	5.71	-0.16	22.25	1.47	1024	1172
L Pars triangularis	0.073	0.075	18.66	10.40	-1.74	39.06	1.52	1023	1173
R Pars triangularis	0.696	0.016	4.97	12.72	-19.98	29.93	0.35	1022	1170

L Pericalcarine	0.544	-0.025	-8.97	14.77	-37.94	20.01	-0.66	940	1113
R Pericalcarine	0.070	-0.077	-27.99	15.44	-58.26	2.29	-1.89	912	1044
L Postcentral	0.272	-0.046	-26.23	23.86	-73.03	20.56	-0.63	995	1132
R Postcentral	0.265	-0.047	-26.47	23.73	-73.00	20.06	-0.67	996	1136
L Posterior cingulate	0.452	0.031	8.42	11.21	-13.56	30.40	0.74	1032	1169
R Posterior cingulate	0.968	0.002	0.41	10.11	-19.41	20.23	0.04	1031	1172
L Precentral	0.623	-0.020	-12.99	26.45	-64.87	38.88	-0.27	1018	1126
R Precentral	0.148	0.060	38.56	26.66	-13.72	90.83	0.80	1021	1134
L Precuneus	0.993	<0.001	0.21	23.99	-46.83	47.25	0.01	1033	1171
R Precuneus	0.043	0.081	50.73	25.06	1.59	99.87	1.33	1032	1172
L Rostral anterior cingulate	0.262	0.046	10.38	9.24	-7.74	28.50	1.26	1022	1141
R Rostral anterior cingulate	0.387	-0.033	-6.90	7.98	-22.55	8.75	-1.03	1025	1157
L Rostral middle frontal	0.404	-0.033	-31.04	37.15	-103.89	41.81	-0.57	1026	1171
R Rostral middle frontal	0.623	-0.019	-19.16	39.00	-95.64	57.31	-0.34	1026	1168
L Superior frontal	0.857	0.007	7.25	40.20	-71.58	86.08	0.10	1013	1149
R Superior frontal	0.205	0.051	51.07	40.30	-27.96	130.10	0.76	1012	1161
L Superior parietal	0.460	-0.031	-24.90	33.71	-91.01	41.20	-0.47	1003	1150
R Superior parietal	0.859	-0.007	-5.84	32.82	-70.20	58.52	-0.11	1017	1160
L Superior temporal	0.647	-0.019	-10.78	23.56	-56.98	35.42	-0.29	904	1062
R Superior temporal	0.464	0.030	14.68	20.04	-24.62	53.97	0.42	979	1113
L Supramarginal	0.399	0.035	26.27	31.16	-34.83	87.37	0.69	997	1116
R Supramarginal	0.702	0.016	10.51	27.52	-43.46	64.49	0.29	1005	1149
L Temporal pole	0.017	-0.097	-9.12	3.80	-16.59	-1.66	-1.92	971	1150
R Temporal pole	0.730	0.014	1.35	3.90	-6.31	9.00	0.32	999	1141
L Transverse temporal	0.206	0.051	5.62	4.44	-3.09	14.33	1.25	1036	1174
R Transverse temporal	0.842	0.008	0.61	3.04	-5.35	6.57	0.18	1035	1176

Results of cortical surface area analysis of PD patients with MoCA scores vs healthy controls. L – Left, R – Right, ROI – region of interest. Banks STS – Banks of the superior temporal sulcus. **Boldface** denotes significant results after Bonferroni correction

Supplementary Table 6c: PD (MoCA group) vs controls – subcortical volumes results

ROI	*p*	*d*	*b*	SE	Lower CI	Upper CI	% diff	NPD
**L Amygdala**	**0.002**	**-0.110**	**-41.39**	**13.56**	**-67.99**	**-14.79**	**-2.78**	**1047**
R Amygdala	0.211	-0.044	-17.61	14.09	-45.24	10.02	-1.11	1049
L Caudate nucleus	0.754	0.012	9.00	28.71	-47.29	65.30	0.26	1044
R Caudate nucleus	0.603	0.020	15.28	29.35	-42.28	72.84	0.44	1045
L Globus pallidus	0.027	0.073	34.92	15.81	3.92	65.92	2.33	1023
R Globus pallidus	0.016	0.081	34.93	14.44	6.61	63.25	2.30	1040
L Hippocampus	0.370	0.033	24.94	27.79	-29.55	79.43	0.64	1033
R Hippocampus	0.964	0.002	1.29	28.57	-54.75	57.32	0.03	1046
L Lateral ventricle	0.945	0.003	28.59	414.90	-785.06	842.25	0.21	1051
R Lateral ventricle	0.319	0.041	376.05	376.93	-363.14	1115.23	3.04	1051
L Nucleus accumbens	0.097	-0.059	-11.72	7.06	-25.56	2.13	-2.45	1048
R Nucleus accumbens	0.614	-0.019	-3.38	6.69	-16.49	9.74	-0.68	1049
L Putamen	0.015	-0.089	-94.81	39.06	-171.41	-18.20	-1.88	1024
R Putamen	0.006	-0.102	-103.34	37.67	-177.21	-29.47	-2.13	1039
L Thalamus	0.004	0.098	133.47	46.19	42.89	224.05	1.86	1009
R Thalamus	0.025	0.080	87.50	39.06	10.90	164.10	1.31	1023

Results of cortical thickness analysis of PD patients with MoCA scores vs healthy controls. L – Left, R – Right, ROI – region of interest. Banks STS – Banks of the superior temporal sulcus. **Boldface** denotes significant results after Bonferroni correction

Supplementary Table 7a: Cortical thickness results - HY1 PD vs HY2 PD

ROI	*p*	*d*	*b*	SE	Lower CI	Upper CI	% diff	NPD	NHC
L Banks STS	0.874	0.008	0.002	0.012	-0.022	0.026	0.086	919	386
R Banks STS	0.767	-0.014	-0.004	0.013	-0.028	0.021	-0.156	1003	421
L Caudal anterior cingulate	0.871	0.008	0.003	0.018	-0.032	0.037	0.110	1046	436
R Caudal anterior cingulate	0.734	-0.017	-0.005	0.016	-0.037	0.026	-0.224	1046	434
L Caudal middle frontal	0.576	0.028	0.006	0.011	-0.015	0.027	0.247	1051	433
R Caudal middle frontal	0.696	-0.020	-0.004	0.011	-0.025	0.017	-0.176	1048	433
L Cuneus	0.375	-0.044	-0.008	0.009	-0.026	0.010	-0.469	980	407
R Cuneus	0.462	-0.034	-0.007	0.010	-0.026	0.012	-0.393	953	403
L Entorhinal	0.761	-0.016	-0.007	0.022	-0.050	0.036	-0.207	1028	428
R Entorhinal	0.688	-0.017	-0.009	0.023	-0.055	0.037	-0.282	998	421
L Frontal pole	0.835	-0.011	-0.004	0.018	-0.038	0.031	-0.138	1053	432
R Frontal pole	0.278	-0.055	-0.019	0.017	-0.053	0.015	-0.725	1052	433
L Fusiform	0.494	-0.033	-0.007	0.011	-0.029	0.014	-0.291	1038	429
R Fusiform	0.366	-0.036	-0.011	0.012	-0.035	0.013	-0.427	1047	433
L Inferior parietal	0.532	-0.031	-0.006	0.010	-0.025	0.013	-0.264	1015	429
R Inferior parietal	0.991	-0.001	<0.001	0.010	-0.019	0.019	-0.005	1018	424
L Inferior temporal	0.686	0.020	0.005	0.011	-0.018	0.027	0.176	1020	412
R Inferior temporal	0.702	-0.017	-0.004	0.012	-0.027	0.018	-0.166	1025	426
L Insula	0.400	-0.045	-0.011	0.013	-0.035	0.014	-0.362	1052	433
R Insula	0.308	-0.047	-0.014	0.013	-0.040	0.013	-0.476	1043	432
L Isthmus cingulate	0.231	-0.061	-0.015	0.013	-0.040	0.010	-0.652	1052	436
R Isthmus cingulate	0.771	-0.014	-0.004	0.013	-0.029	0.022	-0.165	1047	435
L Lateral occipital	0.480	-0.035	-0.007	0.009	-0.025	0.012	-0.317	1036	423
R Lateral occipital	0.286	-0.053	-0.011	0.010	-0.030	0.009	-0.496	1035	429
L Lateral orbitofrontal	0.818	0.011	0.002	0.011	-0.019	0.024	0.099	1055	429
R Lateral orbitofrontal	0.235	-0.051	-0.013	0.011	-0.034	0.008	-0.522	1051	431
L Lingual	0.268	-0.053	-0.009	0.008	-0.026	0.007	-0.488	1020	429
R Lingual	0.122	-0.066	-0.013	0.009	-0.030	0.004	-0.679	996	420
L Medial orbitofrontal	0.980	-0.001	<0.001	0.011	-0.022	0.021	-0.012	1042	428
R Medial orbitofrontal	0.500	-0.030	-0.008	0.011	-0.030	0.015	-0.335	1037	428
L Middle temporal	0.295	-0.052	-0.012	0.012	-0.035	0.011	-0.461	960	389
R Middle temporal	0.604	-0.024	-0.006	0.012	-0.029	0.017	-0.227	1018	420
L Paracentral	0.987	0.001	<0.001	0.012	-0.023	0.023	0.009	1047	435
R Paracentral	0.855	-0.009	-0.002	0.012	-0.025	0.021	-0.095	1040	433
L Parahippocampal	0.131	-0.076	-0.029	0.019	-0.067	0.009	-1.133	1055	435
R Parahippocampal	0.620	-0.024	-0.008	0.017	-0.041	0.024	-0.324	1044	434
L Pars opercularis	0.742	-0.016	-0.003	0.010	-0.023	0.017	-0.140	1053	437
R Pars opercularis	0.974	-0.001	<0.001	0.010	-0.021	0.020	-0.014	1052	436
L Pars orbitalis	0.580	-0.028	-0.007	0.013	-0.032	0.018	-0.274	1050	428
R Pars orbitalis	0.182	-0.065	-0.017	0.013	-0.041	0.008	-0.663	1048	430

L Pars triangularis	0.682	0.020	0.004	0.010	-0.016	0.024	0.182	1053	428
R Pars triangularis	0.919	-0.005	-0.001	0.010	-0.021	0.019	-0.044	1045	430
L Pericalcarine	0.020	-0.105	-0.021	0.009	-0.039	-0.003	-1.346	976	414
R Pericalcarine	0.217	-0.052	-0.012	0.010	-0.031	0.007	-0.762	935	398
L Postcentral	0.998	<0.001	<0.001	0.009	-0.017	0.017	0.001	1002	407
R Postcentral	0.764	-0.013	-0.003	0.009	-0.020	0.015	-0.136	996	412
L Posterior cingulate	0.815	-0.011	-0.003	0.011	-0.024	0.019	-0.108	1054	437
R Posterior cingulate	0.346	-0.042	-0.010	0.011	-0.031	0.011	-0.435	1054	437
L Precentral	0.465	0.036	0.008	0.012	-0.014	0.031	0.357	1010	415
R Precentral	0.939	0.004	0.001	0.012	-0.022	0.024	0.038	1008	424
L Precuneus	0.501	-0.031	-0.006	0.010	-0.025	0.012	-0.292	1053	433
R Precuneus	0.294	-0.049	-0.010	0.010	-0.029	0.009	-0.454	1050	434
L Rostral anterior cingulate	0.540	0.031	0.010	0.016	-0.022	0.042	0.368	1040	429
R Rostral anterior cingulate	0.959	-0.002	-0.001	0.016	-0.033	0.031	-0.032	1038	431
L Rostral middle frontal	0.553	0.027	0.005	0.009	-0.012	0.022	0.228	1042	427
R Rostral middle frontal	0.348	-0.038	-0.009	0.009	-0.026	0.009	-0.381	1048	430
L Superior frontal	0.894	0.007	0.001	0.010	-0.018	0.021	0.052	1025	426
R Superior frontal	0.450	-0.034	-0.007	0.010	-0.027	0.012	-0.295	1026	426
L Superior parietal	0.251	-0.054	-0.011	0.009	-0.029	0.008	-0.521	1019	419
R Superior parietal	0.564	-0.025	-0.005	0.009	-0.024	0.013	-0.263	1023	429
L Superior temporal	0.679	-0.021	-0.005	0.012	-0.028	0.018	-0.186	903	381
R Superior temporal	0.189	-0.055	-0.015	0.011	-0.037	0.007	-0.567	958	407
L Supramarginal	0.632	-0.024	-0.005	0.010	-0.025	0.015	-0.209	1000	416
R Supramarginal	0.364	-0.044	-0.009	0.010	-0.029	0.011	-0.389	1013	422
L Temporal pole	0.285	-0.057	-0.024	0.023	-0.069	0.020	-0.686	1004	416
R Temporal pole	0.142	-0.071	-0.034	0.023	-0.079	0.011	-0.938	1001	419
L Transverse temporal	0.110	-0.083	-0.023	0.014	-0.051	0.005	-1.047	1058	437
R Transverse temporal	0.070	-0.071	-0.028	0.016	-0.059	0.002	-1.255	1057	438

Results of cortical surface area analysis of PD patients with MoCA scores vs healthy controls. L – Left, R – Right, ROI – region of interest. Banks STS – Banks of the superior temporal sulcus. **Boldface** denotes significant results after Bonferroni correction

Supplementary Table 7b: Cortical thickness results - HY2 PD vs HY3PD

ROI	*p*	*d*	*b*	SE	Lower CI	Upper CI	% diff	NPD	NHC
L Banks STS	0.005	-0.165	-0.042	0.015	-0.071	-0.013	-1.831	229	919
R Banks STS	0.013	-0.145	-0.038	0.015	-0.068	-0.008	-1.581	241	1003
L Caudal anterior cingulate	0.356	-0.058	-0.020	0.022	-0.064	0.023	-0.778	257	1046
R Caudal anterior cingulate	0.644	-0.029	-0.009	0.020	-0.049	0.030	-0.377	258	1046
L Caudal middle frontal	0.053	-0.116	-0.025	0.013	-0.049	<0.001	-1.027	259	1051
R Caudal middle frontal	0.046	-0.125	-0.026	0.013	-0.052	<0.001	-1.098	257	1048
L Cuneus	0.045	-0.126	-0.025	0.012	-0.049	-0.001	-1.407	225	980
R Cuneus	0.049	-0.110	-0.024	0.012	-0.048	<0.001	-1.348	230	953
L Entorhinal	0.008	-0.165	-0.071	0.027	-0.123	-0.019	-2.206	259	1028
R Entorhinal	0.019	-0.124	-0.070	0.030	-0.128	-0.012	-2.103	244	998
L Frontal pole	0.367	-0.059	-0.019	0.022	-0.062	0.023	-0.731	260	1053
R Frontal pole	0.474	-0.044	-0.016	0.022	-0.058	0.027	-0.600	257	1052
L Fusiform	0.002	-0.186	-0.042	0.013	-0.069	-0.016	-1.661	254	1038
R Fusiform	0.013	-0.123	-0.038	0.015	-0.068	-0.008	-1.477	255	1047
**L Inferior parietal**	**<0.001**	**-0.233**	**-0.047**	**0.012**	**-0.070**	**-0.023**	**-2.054**	**250**	**1015**
R Inferior parietal	0.005	-0.159	-0.034	0.012	-0.058	-0.010	-1.469	252	1018

L Inferior temporal	0.003	-0.183	-0.043	0.014	-0.071	-0.015	-1.627	234	1020
R Inferior temporal	0.166	-0.079	-0.020	0.014	-0.048	0.008	-0.749	242	1025
L Insula	0.905	-0.008	-0.002	0.015	-0.031	0.028	-0.062	255	1052
R Insula	0.980	0.001	<0.001	0.016	-0.032	0.033	0.015	256	1043
L Isthmus cingulate	0.016	-0.151	-0.037	0.015	-0.067	-0.007	-1.611	259	1052
R Isthmus cingulate	0.061	-0.114	-0.030	0.016	-0.062	0.001	-1.325	258	1047
L Lateral occipital	0.015	-0.148	-0.029	0.012	-0.052	-0.006	-1.389	248	1036
R Lateral occipital	0.011	-0.154	-0.031	0.012	-0.056	-0.007	-1.476	252	1035
L Lateral orbitofrontal	0.016	-0.141	-0.032	0.013	-0.058	-0.006	-1.267	260	1055
R Lateral orbitofrontal	0.310	-0.053	-0.014	0.013	-0.040	0.013	-0.553	259	1051
L Lingual	0.002	-0.185	-0.034	0.011	-0.055	-0.013	-1.766	244	1020
R Lingual	0.035	-0.112	-0.024	0.011	-0.046	-0.002	-1.219	245	996
L Medial orbitofrontal	0.979	0.002	<0.001	0.013	-0.026	0.027	0.015	256	1042
R Medial orbitofrontal	0.961	-0.003	-0.001	0.014	-0.028	0.027	-0.030	256	1037
**L Middle temporal**	**<0.001**	**-0.237**	**-0.055**	**0.014**	**-0.083**	**-0.027**	**-2.073**	**231**	**960**
R Middle temporal	0.007	-0.155	-0.038	0.014	-0.066	-0.011	-1.438	251	1018
L Paracentral	0.056	-0.113	-0.028	0.015	-0.057	0.001	-1.272	257	1047
R Paracentral	0.101	-0.098	-0.024	0.015	-0.053	0.005	-1.068	254	1040
L Parahippocampal	0.133	-0.093	-0.036	0.024	-0.082	0.011	-1.411	259	1055
R Parahippocampal	0.002	-0.188	-0.065	0.021	-0.106	-0.025	-2.599	258	1044
L Pars opercularis	0.048	-0.117	-0.024	0.012	-0.048	<0.001	-1.014	259	1053
R Pars opercularis	0.251	-0.067	-0.014	0.012	-0.039	0.010	-0.593	259	1052
L Pars orbitalis	0.179	-0.082	-0.021	0.016	-0.052	0.010	-0.836	260	1050
R Pars orbitalis	0.678	0.025	0.006	0.015	-0.024	0.037	0.254	258	1048
L Pars triangularis	0.004	-0.172	-0.036	0.013	-0.061	-0.012	-1.593	260	1053
R Pars triangularis	0.459	-0.044	-0.009	0.012	-0.033	0.015	-0.395	259	1045
L Pericalcarine	0.349	-0.055	-0.011	0.012	-0.035	0.012	-0.732	219	976
R Pericalcarine	0.109	-0.086	-0.021	0.013	-0.046	0.005	-1.300	222	935
L Postcentral	0.075	-0.101	-0.019	0.011	-0.040	0.002	-0.970	248	1002
R Postcentral	0.135	-0.080	-0.016	0.011	-0.038	0.005	-0.849	244	996
L Posterior cingulate	0.142	-0.091	-0.020	0.014	-0.048	0.007	-0.861	259	1054
R Posterior cingulate	0.006	-0.154	-0.037	0.013	-0.063	-0.010	-1.571	259	1054
L Precentral	0.017	-0.140	-0.033	0.014	-0.061	-0.006	-1.413	246	1010
R Precentral	0.042	-0.120	-0.029	0.014	-0.056	-0.001	-1.226	247	1008
**L Precuneus**	**<0.001**	**-0.207**	**-0.042**	**0.012**	**-0.066**	**-0.019**	**-1.920**	**257**	**1053**
R Precuneus	0.003	-0.176	-0.036	0.012	-0.059	-0.012	-1.605	258	1050
L Rostral anterior cingulate	0.361	-0.060	-0.018	0.020	-0.056	0.020	-0.649	257	1040
R Rostral anterior cingulate	0.019	0.140	0.048	0.020	0.008	0.088	1.790	255	1038
**L Rostral middle frontal**	**0.001**	**-0.197**	**-0.038**	**0.011**	**-0.059**	**-0.016**	**-1.672**	**258**	**1042**
R Rostral middle frontal	0.193	-0.068	-0.015	0.011	-0.037	0.007	-0.663	259	1048
L Superior frontal	0.014	-0.153	-0.030	0.012	-0.054	-0.006	-1.183	252	1025
R Superior frontal	0.034	-0.121	-0.025	0.012	-0.049	-0.002	-1.011	251	1026
L Superior parietal	0.006	-0.156	-0.032	0.012	-0.055	-0.009	-1.565	244	1019
R Superior parietal	0.008	-0.145	-0.031	0.012	-0.055	-0.008	-1.538	247	1023
**L Superior temporal**	**0.001**	**-0.214**	**-0.050**	**0.014**	**-0.078**	**-0.022**	**-1.940**	**212**	**903**
R Superior temporal	0.024	-0.115	-0.030	0.013	-0.057	-0.004	-1.188	239	958
L Supramarginal	0.001	-0.201	-0.042	0.012	-0.066	-0.017	-1.771	246	1000
R Supramarginal	0.005	-0.170	-0.035	0.012	-0.060	-0.011	-1.492	250	1013
L Temporal pole	0.847	-0.013	-0.005	0.028	-0.061	0.050	-0.155	238	1004
R Temporal pole	0.195	-0.077	-0.037	0.029	-0.093	0.019	-1.038	241	1001

L Transverse temporal	0.336	-0.059	-0.017	0.017	-0.051	0.017	-0.779	260	1058
R Transverse temporal	0.050	-0.093	-0.037	0.019	-0.075	<0.001	-1.686	259	1057

Results of cortical surface area analysis of PD patients with MoCA scores vs healthy controls. L – Left, R – Right, ROI – region of interest. Banks STS – Banks of the superior temporal sulcus. **Boldface** denotes significant results after Bonferroni correction

Supplementary Table 7c: Cortical thickness results - HY3 PD vs HY4&5 PD

ROI	*p*	*d*	*b*	SE	Lower CI	Upper CI	% diff	NPD	NHC
L Banks STS	0.028	-0.229	-0.055	0.025	-0.103	-0.006	-2.461	73	229
R Banks STS	0.630	-0.048	-0.011	0.023	-0.057	0.035	-0.487	76	241
L Caudal anterior cingulate	0.600	0.059	0.020	0.039	-0.056	0.096	0.766	80	257
R Caudal anterior cingulate	0.106	-0.182	-0.059	0.036	-0.130	0.013	-2.321	81	258
L Caudal middle frontal	0.182	-0.136	-0.028	0.021	-0.069	0.013	-1.184	81	259
R Caudal middle frontal	0.771	0.030	0.007	0.023	-0.038	0.051	0.282	81	257
L Cuneus	0.476	0.078	0.015	0.021	-0.026	0.056	0.861	74	225
R Cuneus	0.659	0.039	0.009	0.020	-0.030	0.047	0.489	73	230
L Entorhinal	0.915	0.012	0.005	0.044	-0.083	0.092	0.154	81	259
R Entorhinal	0.834	-0.018	-0.010	0.048	-0.105	0.085	-0.321	81	244
L Frontal pole	0.312	-0.124	-0.036	0.035	-0.105	0.034	-1.345	81	260
R Frontal pole	0.006	-0.295	-0.094	0.034	-0.161	-0.027	-3.607	81	257
L Fusiform	0.104	-0.155	-0.033	0.020	-0.073	0.007	-1.331	81	254
R Fusiform	0.081	-0.136	-0.044	0.025	-0.093	0.005	-1.726	79	255
L Inferior parietal	0.431	-0.078	-0.016	0.020	-0.056	0.024	-0.735	78	250
R Inferior parietal	0.148	-0.138	-0.031	0.021	-0.072	0.011	-1.369	80	252
L Inferior temporal	0.176	-0.144	-0.031	0.023	-0.076	0.014	-1.201	79	234
R Inferior temporal	0.066	-0.165	-0.039	0.021	-0.080	0.003	-1.482	79	242
L Insula	0.175	-0.161	-0.031	0.023	-0.076	0.014	-1.082	81	255
R Insula	0.008	-0.243	-0.070	0.027	-0.123	-0.018	-2.504	81	256
L Isthmus cingulate	0.148	-0.162	-0.036	0.025	-0.085	0.013	-1.600	81	259
R Isthmus cingulate	0.649	-0.048	-0.012	0.026	-0.064	0.040	-0.534	81	258
L Lateral occipital	0.286	0.108	0.022	0.020	-0.018	0.061	1.056	76	248
R Lateral occipital	0.381	-0.094	-0.018	0.021	-0.059	0.022	-0.857	78	252
L Lateral orbitofrontal	0.237	-0.121	-0.025	0.021	-0.065	0.016	-0.994	81	260
R Lateral orbitofrontal	0.003	-0.244	-0.060	0.020	-0.099	-0.020	-2.433	81	259
L Lingual	0.602	-0.051	-0.009	0.017	-0.043	0.025	-0.488	77	244
R Lingual	0.647	-0.040	-0.009	0.020	-0.048	0.030	-0.467	75	245
L Medial orbitofrontal	0.034	-0.250	-0.046	0.022	-0.089	-0.004	-1.958	81	256
R Medial orbitofrontal	0.002	-0.284	-0.067	0.021	-0.109	-0.026	-2.882	81	256
L Middle temporal	0.168	-0.146	-0.031	0.022	-0.074	0.013	-1.191	75	231
R Middle temporal	0.016	-0.222	-0.050	0.020	-0.090	-0.009	-1.901	80	251
L Paracentral	0.728	0.034	0.008	0.023	-0.038	0.054	0.372	81	257
R Paracentral	0.922	-0.010	-0.002	0.024	-0.051	0.046	-0.108	81	254
L Parahippocampal	0.778	-0.032	-0.011	0.040	-0.090	0.068	-0.462	80	259
R Parahippocampal	0.898	-0.014	-0.004	0.035	-0.073	0.064	-0.187	81	258
L Pars opercularis	0.040	-0.211	-0.040	0.019	-0.078	-0.002	-1.704	81	259
R Pars opercularis	0.298	-0.100	-0.021	0.020	-0.061	0.019	-0.883	81	259
L Pars orbitalis	0.071	-0.198	-0.051	0.028	-0.105	0.004	-2.019	81	260

R Pars orbitalis	0.013	-0.272	-0.067	0.027	-0.119	-0.014	-2.648	80	258
L Pars triangularis	0.974	-0.003	-0.001	0.021	-0.041	0.040	-0.030	80	260
R Pars triangularis	0.451	-0.076	-0.016	0.022	-0.059	0.026	-0.721	81	259
L Pericalcarine	0.614	-0.057	-0.012	0.023	-0.058	0.034	-0.768	74	219
R Pericalcarine	0.530	0.056	0.014	0.022	-0.029	0.056	0.873	69	222
L Postcentral	0.041	-0.200	-0.036	0.018	-0.071	-0.002	-1.877	79	248
R Postcentral	0.204	-0.107	-0.023	0.018	-0.058	0.012	-1.217	80	244
L Posterior cingulate	0.466	-0.082	-0.017	0.023	-0.062	0.028	-0.711	81	259
R Posterior cingulate	0.079	-0.157	-0.038	0.022	-0.081	0.004	-1.661	81	259
L Precentral	0.249	-0.108	-0.025	0.021	-0.066	0.017	-1.058	79	246
R Precentral	0.950	0.006	0.001	0.022	-0.042	0.044	0.060	80	247
L Precuneus	0.651	-0.044	-0.010	0.021	-0.051	0.032	-0.443	80	257
R Precuneus	0.565	-0.054	-0.011	0.019	-0.047	0.026	-0.495	81	258
L Rostral anterior cingulate	0.258	-0.132	-0.034	0.030	-0.094	0.025	-1.245	81	257
R Rostral anterior cingulate	0.285	-0.106	-0.037	0.034	-0.105	0.031	-1.352	81	255
L Rostral middle frontal	0.662	-0.043	-0.008	0.019	-0.045	0.029	-0.369	81	258
R Rostral middle frontal	0.008	-0.242	-0.057	0.021	-0.098	-0.015	-2.528	80	259
L Superior frontal	0.932	0.009	0.002	0.019	-0.036	0.039	0.065	80	252
R Superior frontal	0.868	0.016	0.003	0.019	-0.034	0.040	0.127	80	251
L Superior parietal	0.922	-0.009	-0.002	0.020	-0.042	0.038	-0.100	81	244
R Superior parietal	0.680	-0.035	-0.008	0.020	-0.047	0.030	-0.404	81	247
L Superior temporal	0.221	-0.136	-0.028	0.023	-0.072	0.017	-1.114	74	212
R Superior temporal	0.145	-0.121	-0.031	0.021	-0.072	0.011	-1.233	77	239
L Supramarginal	0.312	-0.097	-0.020	0.020	-0.058	0.019	-0.862	81	246
R Supramarginal	0.026	-0.216	-0.045	0.020	-0.085	-0.005	-1.954	80	250
L Temporal pole	0.058	-0.236	-0.079	0.041	-0.160	0.003	-2.252	78	238
R Temporal pole	0.119	-0.148	-0.068	0.043	-0.153	0.017	-1.925	80	241
L Transverse temporal	0.040	-0.205	-0.057	0.028	-0.112	-0.003	-2.768	81	260
R Transverse temporal	0.581	-0.038	-0.018	0.032	-0.081	0.045	-0.834	80	259

Results of cortical surface area analysis of PD patients with MoCA scores vs healthy controls. L – Left, R – Right, ROI – region of interest. Banks STS – Banks of the superior temporal sulcus. **Boldface** denotes significant results after Bonferroni correction

Supplementary Table 7d: Cortical surface area results - HY1 PD vs HY2 PD

ROI	*p*	*d*	*b*	SE	Lower CI	Upper CI	% diff	NPD	NHC
L Banks STS	0.086	0.100	16.49	9.58	-2.31	35.29	1.666	917	386
R Banks STS	0.096	0.090	13.67	8.21	-2.43	29.77	1.499	1004	420
L Caudal anterior cingulate	0.202	0.070	10.29	8.06	-5.52	26.10	1.624	1045	435
R Caudal anterior cingulate	0.786	-0.015	-2.51	9.25	-20.65	15.64	-0.328	1043	433
L Caudal middle frontal	0.265	-0.060	-22.01	19.75	-60.75	16.73	-0.974	1050	432
R Caudal middle frontal	0.649	-0.025	-8.96	19.64	-47.49	29.58	-0.431	1050	433
L Cuneus	0.346	-0.054	-11.66	12.36	-35.91	12.58	-0.813	980	407
R Cuneus	0.686	0.023	5.02	12.43	-19.37	29.42	0.340	950	403
L Entorhinal	0.791	0.015	1.40	5.30	-8.99	11.80	0.343	1028	428
R Entorhinal	0.278	0.060	5.27	4.86	-4.26	14.81	1.503	998	422
L Frontal pole	0.565	0.029	1.14	1.99	-2.76	5.04	0.563	1052	431
R Frontal pole	0.046	0.104	5.13	2.57	0.09	10.16	1.892	1053	432
L Fusiform	0.955	-0.003	-1.22	21.88	-44.15	41.70	-0.039	1038	428
R Fusiform	0.380	-0.047	-19.20	21.85	-62.07	23.67	-0.624	1045	433
L Inferior parietal	0.948	-0.003	-2.10	32.48	-65.81	61.61	-0.047	1015	429
R Inferior parietal	0.445	-0.042	-27.98	36.66	-99.91	43.94	-0.526	1020	424

L Inferior temporal	0.201	-0.070	-33.26	26.00	-84.27	17.75	-1.030	1022	412
R Inferior temporal	0.883	-0.008	-3.42	23.25	-49.03	42.18	-0.112	1025	426
L Insula	0.573	0.028	7.18	12.74	-17.82	32.17	0.327	1052	433
R Insula	0.597	0.027	7.90	14.95	-21.42	37.22	0.352	1043	432
L Isthmus cingulate	0.381	-0.047	-8.56	9.78	-27.74	10.61	-0.831	1052	436
R Isthmus cingulate	0.721	-0.019	-3.20	8.94	-20.74	14.35	-0.337	1047	435
L Lateral occipital	0.197	-0.068	-42.45	32.87	-106.93	22.04	-0.897	1037	424
R Lateral occipital	0.353	-0.050	-31.14	33.54	-96.95	34.66	-0.677	1036	429
L Lateral orbitofrontal	0.323	-0.055	-15.09	15.27	-45.04	14.87	-0.589	1054	429
R Lateral orbitofrontal	0.039	-0.111	-34.30	16.63	-66.92	-1.68	-1.358	1050	431
**L Lingual**	**<0.001**	**-0.224**	**-100.88**	**24.42**	**-148.78**	**-52.98**	**-3.329**	**1019**	**429**
R Lingual	0.008	-0.145	-64.58	24.34	-112.34	-16.82	-2.129	995	421
L Medial orbitofrontal	0.937	0.004	1.01	12.79	-24.07	26.10	0.055	1042	428
R Medial orbitofrontal	0.790	-0.014	-2.93	11.03	-24.56	18.70	-0.163	1038	428
L Middle temporal	0.987	-0.001	-0.35	21.40	-42.34	41.63	-0.012	960	389
R Middle temporal	0.623	0.026	10.60	21.58	-31.72	52.92	0.323	1018	420
L Paracentral	0.808	0.013	2.51	10.31	-17.72	22.74	0.185	1046	435
R Paracentral	0.748	0.017	4.05	12.61	-20.69	28.78	0.265	1041	433
L Parahippocampal	0.873	0.009	1.00	6.24	-11.25	13.25	0.146	1054	434
R Parahippocampal	0.437	0.042	4.59	5.91	-7.00	16.18	0.691	1043	433
L Pars opercularis	0.961	-0.003	-0.69	14.03	-28.20	26.83	-0.043	1053	438
R Pars opercularis	0.046	-0.111	-24.83	12.41	-49.18	-0.48	-1.819	1053	436
L Pars orbitalis	0.468	-0.039	-3.29	4.53	-12.18	5.60	-0.533	1049	428
R Pars orbitalis	0.971	-0.002	-0.21	5.74	-11.46	11.05	-0.027	1050	430
L Pars triangularis	0.399	-0.047	-9.03	10.70	-30.02	11.96	-0.725	1053	428
R Pars triangularis	0.330	-0.053	-13.53	13.88	-40.76	13.70	-0.942	1046	430
L Pericalcarine	0.012	-0.141	-35.32	14.02	-62.82	-7.82	-2.588	975	414
R Pericalcarine	0.011	-0.145	-38.45	15.12	-68.11	-8.79	-2.590	934	398
L Postcentral	0.727	0.019	8.84	25.30	-40.78	58.46	0.214	1002	407
R Postcentral	0.074	0.101	45.13	25.26	-4.42	94.68	1.147	994	412
L Posterior cingulate	0.741	0.018	4.32	13.08	-21.34	29.98	0.376	1056	437
R Posterior cingulate	0.655	0.023	4.54	10.14	-15.36	24.43	0.388	1053	438
L Precentral	0.539	0.033	16.69	27.14	-36.55	69.93	0.344	1009	415
R Precentral	0.540	0.032	17.48	28.50	-38.42	73.38	0.358	1009	424
L Precuneus	0.436	0.042	18.78	24.11	-28.51	66.06	0.510	1053	434
R Precuneus	0.210	0.067	32.78	26.14	-18.50	84.06	0.850	1050	434
L Rostral anterior cingulate	0.169	0.075	12.44	9.03	-5.28	30.16	1.514	1036	428
R Rostral anterior cingulate	0.720	-0.019	-2.84	7.91	-18.35	12.68	-0.422	1040	431
L Rostral middle frontal	0.070	-0.095	-68.83	37.97	-143.31	5.65	-1.257	1042	427
R Rostral middle frontal	0.241	-0.062	-45.96	39.22	-122.89	30.97	-0.811	1050	430
L Superior frontal	0.072	-0.093	-70.79	39.29	-147.86	6.28	-1.007	1026	426
R Superior frontal	0.451	-0.040	-29.90	39.66	-107.71	47.90	-0.439	1027	426
L Superior parietal	0.267	-0.060	-37.68	33.92	-104.22	28.86	-0.713	1019	419
R Superior parietal	0.305	-0.055	-34.65	33.78	-100.91	31.61	-0.652	1021	429
L Superior temporal	0.544	0.033	14.38	23.67	-32.06	60.83	0.391	902	381
R Superior temporal	0.121	0.083	31.74	20.43	-8.34	71.81	0.903	958	407
L Supramarginal	0.670	-0.023	-12.85	30.18	-72.06	46.36	-0.337	1001	415
R Supramarginal	0.300	0.056	28.85	27.85	-25.79	83.50	0.803	1016	423
L Temporal pole	0.581	-0.031	-2.05	3.72	-9.35	5.24	-0.437	1005	416
R Temporal pole	0.920	-0.005	-0.39	3.89	-8.02	7.24	-0.092	1000	419

L Transverse temporal	0.644	0.025	2.03	4.39	-6.58	10.64	0.446	1058	437
R Transverse temporal	0.885	0.008	0.46	3.17	-5.76	6.68	0.135	1056	438

Results of cortical surface area analysis of PD patients with MoCA scores vs healthy controls. L – Left, R – Right, ROI – region of interest. Banks STS – Banks of the superior temporal sulcus. **Boldface** denotes significant results after Bonferroni correction

Supplementary Table 7e: Cortical surface area results - HY2 PD vs HY3 PD

ROI	*p*	*d*	*b*	SE	Lower CI	Upper CI	% diff	NPD	NHC
L Banks STS	0.003	-0.209	-35.13	11.77	-58.22	-12.05	-3.49	230	917
R Banks STS	0.002	-0.203	-31.58	10.27	-51.73	-11.44	-3.43	240	1004
L Caudal anterior cingulate	0.040	-0.137	-20.94	10.19	-40.94	-0.95	-3.27	258	1045
R Caudal anterior cingulate	0.408	-0.055	-9.50	11.50	-32.06	13.05	-1.24	258	1043
L Caudal middle frontal	0.153	-0.094	-35.41	24.77	-84.01	13.18	-1.58	258	1050
R Caudal middle frontal	0.059	-0.127	-46.72	24.71	-95.19	1.76	-2.26	256	1050
L Cuneus	0.810	-0.017	-3.71	15.39	-33.91	26.49	-0.26	225	980
R Cuneus	0.032	-0.152	-32.51	15.16	-62.25	-2.76	-2.20	230	950
L Entorhinal	0.631	-0.032	-3.15	6.56	-16.01	9.72	-0.76	258	1028
R Entorhinal	0.134	-0.101	-9.21	6.14	-21.26	2.85	-2.58	245	998
L Frontal pole	0.006	-0.166	-6.71	2.42	-11.47	-1.96	-3.27	260	1052
R Frontal pole	0.135	-0.091	-4.72	3.15	-10.90	1.47	-1.70	259	1053
L Fusiform	0.005	-0.181	-76.04	27.01	-129.04	-23.04	-2.42	254	1038
R Fusiform	0.006	-0.180	-75.71	27.41	-129.48	-21.95	-2.48	254	1045
L Inferior parietal	0.007	-0.176	-111.35	41.05	-191.89	-30.81	-2.48	249	1015
R Inferior parietal	0.008	-0.176	-121.62	45.70	-211.29	-31.95	-2.29	252	1020
L Inferior temporal	0.048	-0.133	-63.40	31.97	-126.12	-0.69	-1.98	234	1022
R Inferior temporal	0.016	-0.159	-70.91	29.39	-128.57	-13.25	-2.32	242	1025
L Insula	0.233	-0.072	-19.17	16.08	-50.72	12.38	-0.87	255	1052
R Insula	0.052	-0.122	-36.94	18.99	-74.21	0.32	-1.63	256	1043
L Isthmus cingulate	0.848	-0.012	-2.32	12.15	-26.15	21.51	-0.23	260	1052
R Isthmus cingulate	0.643	-0.030	-5.05	10.87	-26.36	16.27	-0.53	257	1047
L Lateral occipital	0.001	-0.208	-131.24	41.20	-212.07	-50.41	-2.79	248	1037
R Lateral occipital	0.055	-0.128	-80.72	41.99	-163.11	1.66	-1.77	252	1036
L Lateral orbitofrontal	0.420	-0.053	-15.44	19.15	-53.02	22.13	-0.61	260	1054
R Lateral orbitofrontal	0.178	-0.087	-28.16	20.91	-69.19	12.87	-1.13	258	1050
L Lingual	0.608	-0.034	-15.62	30.46	-75.39	44.15	-0.53	243	1019
R Lingual	0.119	-0.103	-46.61	29.91	-105.29	12.07	-1.57	245	995
L Medial orbitofrontal	0.001	-0.217	-52.38	15.69	-83.16	-21.59	-2.86	256	1042
R Medial orbitofrontal	0.104	-0.105	-22.74	13.99	-50.20	4.71	-1.26	256	1038
L Middle temporal	0.021	-0.155	-62.68	27.03	-115.72	-9.65	-2.11	231	960
**R Middle temporal**	**<0.001**	**-0.269**	**-111.87**	**26.70**	**-164.26**	**-59.48**	**-3.38**	**251**	**1018**
L Paracentral	0.904	-0.008	-1.58	13.14	-27.37	24.21	-0.12	255	1046
R Paracentral	0.836	-0.014	-3.36	16.16	-35.06	28.35	-0.22	254	1041
L Parahippocampal	0.017	-0.154	-18.33	7.70	-33.43	-3.22	-2.66	259	1054
R Parahippocampal	0.002	-0.205	-23.63	7.54	-38.42	-8.84	-3.54	257	1043
L Pars opercularis	0.190	-0.086	-21.91	16.71	-54.69	10.87	-1.38	259	1053
R Pars opercularis	0.291	-0.071	-15.52	14.70	-44.36	13.32	-1.16	259	1053
L Pars orbitalis	0.884	-0.009	-0.83	5.72	-12.06	10.39	-0.14	260	1049

R Pars orbitalis	0.015	-0.160	-17.36	7.09	-31.28	-3.44	-2.29	258	1050
L Pars triangularis	0.819	0.015	3.04	13.32	-23.09	29.18	0.25	260	1053
R Pars triangularis	0.595	-0.035	-9.03	17.01	-42.40	24.34	-0.64	259	1046
L Pericalcarine	0.684	0.029	7.94	19.54	-30.40	46.28	0.60	219	975
R Pericalcarine	0.694	0.028	7.46	18.92	-29.66	44.58	0.51	222	934
L Postcentral	0.002	-0.206	-97.73	31.57	-159.68	-35.79	-2.36	248	1002
R Postcentral	0.127	-0.104	-49.14	32.20	-112.32	14.04	-1.23	244	994
L Posterior cingulate	0.134	-0.099	-24.70	16.48	-57.03	7.63	-2.14	257	1056
R Posterior cingulate	0.204	-0.081	-15.75	12.40	-40.08	8.57	-1.34	259	1053
L Precentral	0.046	-0.130	-69.20	34.70	-137.28	-1.12	-1.42	246	1009
R Precentral	0.009	-0.169	-93.78	35.72	-163.86	-23.70	-1.92	247	1009
L Precuneus	0.008	-0.174	-78.66	29.64	-136.81	-20.51	-2.12	258	1053
R Precuneus	0.025	-0.146	-71.20	31.78	-133.54	-8.86	-1.83	258	1050
L Rostral anterior cingulate	0.865	0.011	2.00	11.73	-21.01	25.00	0.24	257	1036
R Rostral anterior cingulate	0.170	-0.089	-13.29	9.67	-32.27	5.68	-1.98	255	1040
L Rostral middle frontal	0.059	-0.119	-92.27	48.82	-188.05	3.51	-1.70	257	1042
R Rostral middle frontal	0.098	-0.105	-80.73	48.73	-176.32	14.86	-1.43	259	1050
L Superior frontal	0.343	-0.060	-47.63	50.21	-146.14	50.89	-0.68	252	1026
R Superior frontal	0.116	-0.101	-80.88	51.35	-181.63	19.87	-1.20	251	1027
L Superior parietal	0.048	-0.132	-84.23	42.53	-167.66	-0.80	-1.60	244	1019
R Superior parietal	0.081	-0.115	-74.41	42.55	-157.90	9.08	-1.41	247	1021
L Superior temporal	0.300	-0.071	-32.22	31.08	-93.20	28.76	-0.87	212	902
R Superior temporal	0.001	-0.218	-84.51	25.65	-134.83	-34.18	-2.38	239	958
L Supramarginal	0.006	-0.181	-106.24	38.50	-181.77	-30.71	-2.77	245	1001
R Supramarginal	0.010	-0.172	-89.55	34.63	-157.50	-21.60	-2.47	249	1016
L Temporal pole	0.284	-0.074	-5.04	4.70	-14.26	4.18	-1.08	239	1005
R Temporal pole	0.488	0.047	3.35	4.83	-6.12	12.82	0.79	242	1000
L Transverse temporal	0.774	-0.019	-1.55	5.40	-12.15	9.04	-0.34	259	1058
R Transverse temporal	0.383	-0.057	-3.44	3.94	-11.16	4.29	-1.01	259	1056

Results of cortical surface area analysis of PD patients with MoCA scores vs healthy controls. L – Left, R – Right, ROI – region of interest. Banks STS – Banks of the superior temporal sulcus. **Boldface** denotes significant results after Bonferroni correction

Supplementary Table 7f: Cortical surface area results - HY3 PD vs HY4&5 PD

ROI	*p*	*d*	*b*	SE	Lower CI	Upper CI	% diff	NPD	NHC
L Banks STS	0.656	0.053	8.27	18.53	-28.21	44.74	0.85	72	230
R Banks STS	0.672	-0.053	-7.02	16.57	-39.62	25.59	-0.81	77	240
L Caudal anterior cingulate	0.369	-0.114	-14.93	16.60	-47.59	17.74	-2.44	80	258
R Caudal anterior cingulate	0.577	-0.070	-10.59	18.94	-47.86	26.68	-1.46	81	258
L Caudal middle frontal	0.087	0.208	74.25	43.19	-10.73	159.23	3.41	81	258
R Caudal middle frontal	0.121	0.193	67.20	43.28	-17.95	152.36	3.32	80	256
L Cuneus	0.101	0.222	42.51	25.83	-8.35	93.37	3.02	74	225
R Cuneus	0.552	0.079	15.50	26.02	-35.72	66.73	1.06	72	230
L Entorhinal	0.381	-0.102	-9.28	10.58	-30.09	11.53	-2.26	81	258
R Entorhinal	0.581	0.068	5.68	10.29	-14.56	25.92	1.60	81	245

L Frontal pole	0.037	0.213	7.76	3.70	0.48	15.04	3.77	81	260
R Frontal pole	0.437	0.087	4.18	5.37	-6.39	14.76	1.49	81	259
L Fusiform	0.641	0.053	20.16	43.20	-64.84	105.16	0.66	81	254
R Fusiform	0.750	-0.040	-14.18	44.51	-101.76	73.39	-0.48	81	254
L Inferior parietal	0.983	-0.003	-1.51	71.94	-143.08	140.06	-0.04	79	249
R Inferior parietal	0.955	0.007	4.57	80.71	-154.25	163.38	0.09	81	252
L Inferior temporal	0.622	-0.060	-25.81	52.26	-128.67	77.04	-0.83	79	234
R Inferior temporal	0.494	0.084	33.16	48.41	-62.12	128.43	1.12	80	242
L Insula	0.843	-0.023	-6.02	30.42	-65.87	53.84	-0.27	81	255
R Insula	0.647	-0.053	-14.99	32.73	-79.39	49.41	-0.66	81	256
L Isthmus cingulate	0.538	0.075	12.52	20.33	-27.48	52.51	1.23	80	260
R Isthmus cingulate	0.066	0.213	33.59	18.20	-2.22	69.40	3.56	81	257
L Lateral occipital	0.580	0.071	37.73	68.02	-96.12	171.58	0.82	76	248
R Lateral occipital	0.138	0.188	107.92	72.55	-34.84	250.68	2.38	78	252
L Lateral orbitofrontal	0.203	-0.157	-42.07	33.01	-107.01	22.86	-1.68	81	260
R Lateral orbitofrontal	0.745	-0.039	-11.87	36.46	-83.61	59.87	-0.48	80	258
L Lingual	0.607	0.068	24.79	48.20	-70.08	119.65	0.87	76	243
R Lingual	0.260	0.147	53.86	47.74	-40.08	147.80	1.85	76	245
L Medial orbitofrontal	0.854	-0.022	-4.70	25.61	-55.08	45.68	-0.26	81	256
R Medial orbitofrontal	0.107	0.190	39.38	24.33	-8.49	87.26	2.18	81	256
L Middle temporal	0.394	0.108	42.06	49.26	-54.90	139.01	1.46	75	231
R Middle temporal	0.383	0.105	38.77	44.39	-48.58	126.13	1.22	80	251
L Paracentral	0.889	0.016	3.26	23.43	-42.84	49.36	0.24	81	255
R Paracentral	0.794	0.031	6.87	26.31	-44.90	58.65	0.44	81	254
L Parahippocampal	0.455	0.094	9.17	12.26	-14.95	33.28	1.40	81	259
R Parahippocampal	0.463	0.093	8.42	11.44	-14.10	30.94	1.33	81	257
L Pars opercularis	0.673	0.052	11.87	28.06	-43.34	67.08	0.76	81	259
R Pars opercularis	0.343	-0.120	-22.73	23.92	-69.80	24.34	-1.75	81	259
L Pars orbitalis	0.643	-0.054	-4.59	9.89	-24.05	14.86	-0.74	81	260
R Pars orbitalis	0.731	-0.042	-3.97	11.55	-26.70	18.76	-0.53	81	258
L Pars triangularis	0.359	-0.115	-19.92	21.71	-62.62	22.79	-1.60	80	260
R Pars triangularis	0.505	-0.081	-19.39	29.04	-76.53	37.75	-1.37	81	259
L Pericalcarine	0.356	0.126	36.74	39.71	-41.43	114.92	2.80	73	219
R Pericalcarine	0.863	0.024	5.63	32.56	-58.49	69.74	0.39	69	222
L Postcentral	0.566	0.073	29.31	51.06	-71.17	129.79	0.73	79	248
R Postcentral	0.959	-0.007	-2.81	54.32	-109.70	104.08	-0.07	80	244
L Posterior cingulate	0.571	-0.073	-11.05	19.48	-49.38	27.28	-0.99	81	257
R Posterior cingulate	0.266	-0.142	-22.72	20.38	-62.82	17.38	-1.99	79	259
L Precentral	0.216	0.149	80.31	64.73	-47.06	207.69	1.66	79	246
R Precentral	0.129	0.184	91.99	60.38	-26.82	210.80	1.91	80	247
L Precuneus	0.723	-0.045	-17.22	48.51	-112.66	78.22	-0.48	81	258
R Precuneus	0.580	-0.070	-28.86	52.10	-131.37	73.65	-0.76	81	258
L Rostral anterior cingulate	0.418	-0.100	-16.96	20.93	-58.14	24.22	-2.04	81	257
R Rostral anterior cingulate	0.719	-0.043	-5.73	15.89	-37.00	25.54	-0.90	81	255
L Rostral middle frontal	0.352	0.109	76.97	82.61	-85.58	239.52	1.43	81	257
R Rostral middle frontal	0.165	0.164	115.51	83.03	-47.85	278.86	2.07	81	259
L Superior frontal	0.783	0.033	25.15	91.44	-154.78	205.08	0.36	80	252
R Superior frontal	0.580	0.066	50.34	90.97	-128.67	229.34	0.76	81	251
L Superior parietal	0.429	0.099	57.81	73.04	-85.92	201.54	1.12	81	244
R Superior parietal	0.883	-0.018	-10.36	70.21	-148.52	127.80	-0.20	81	247

L Superior temporal	0.984	0.003	1.03	51.30	-99.99	102.04	0.03	74	212
R Superior temporal	0.999	<0.001	-0.06	41.99	-82.70	82.58	0.00	78	239
L Supramarginal	0.978	0.004	1.93	68.89	-133.63	137.49	0.05	81	245
R Supramarginal	0.583	-0.071	-31.00	56.33	-141.84	79.85	-0.87	81	249
L Temporal pole	0.389	0.109	6.92	8.03	-8.88	22.73	1.50	78	239
R Temporal pole	0.375	0.107	7.15	8.05	-8.69	22.98	1.66	80	242
L Transverse temporal	0.340	0.119	8.64	9.05	-9.15	26.44	1.91	81	259
R Transverse temporal	0.851	0.024	1.18	6.26	-11.14	13.50	0.35	81	259

Results of cortical surface area analysis of PD patients with MoCA scores vs healthy controls. L – Left, R – Right, ROI – region of interest. Banks STS – Banks of the superior temporal sulcus. **Boldface** denotes significant results after Bonferroni correction

Supplementary Table 7g: Subcortical volume results - HY1 PD vs HY2 PD

ROI	*p*	*d*	*b*	SE	Lower CI	Upper CI	% diff	NPD
**L Amygdala**	0.697	-0.018	-4.89	12.56	-29.54	19.75	-0.334	1052
R Amygdala	0.060	-0.087	-25.35	13.48	-51.79	1.09	-1.595	1049
L Caudate nucleus	0.012	-0.131	-70.92	28.08	-126.00	-15.85	-2.023	1054
R Caudate nucleus	0.039	-0.106	-57.96	28.02	-112.92	-2.99	-1.625	1048
L Globus pallidus	0.757	0.015	4.85	15.69	-25.93	35.63	0.327	1017
R Globus pallidus	0.263	0.056	15.58	13.90	-11.69	42.84	1.048	1044
L Hippocampus	0.110	-0.081	-43.45	27.14	-96.69	9.79	-1.095	1034
R Hippocampus	0.059	-0.093	-51.60	27.30	-105.15	1.96	-1.262	1047
L Lateral ventricle	0.839	-0.011	-84.81	416.27	-901.35	731.74	-0.611	1063
R Lateral ventricle	0.762	-0.017	-119.76	394.62	-893.84	654.33	-0.919	1063
L Nucleus accumbens	0.887	0.007	0.95	6.68	-12.15	14.06	0.198	1052
R Nucleus accumbens	0.443	-0.037	-4.93	6.43	-17.54	7.67	-0.990	1045
L Putamen	0.858	-0.009	-6.88	38.54	-82.48	68.73	-0.139	1026
R Putamen	0.698	-0.019	-14.23	36.72	-86.26	57.79	-0.300	1051
L Thalamus	0.119	-0.069	-73.24	46.97	-165.37	18.90	-0.993	1009
R Thalamus	0.427	-0.037	-30.81	38.79	-106.90	45.28	-0.455	1031

Results of cortical thickness analysis of PD patients with MoCA scores vs healthy controls. L – Left, R – Right, ROI – region of interest. Banks STS – Banks of the superior temporal sulcus. **Boldface** denotes significant results after Bonferroni correction

Supplementary Table 7h: Subcortical volume results - HY2 PD vs HY3 PD

ROI	*p*	*d*	*b*	SE	Lower CI	Upper CI	% diff	NPD
**L Amygdala**	**<0.001**	**-0.285**	**-73.61**	**15.75**	**-104.51**	**-42.71**	**-5.07**	**262**
**R Amygdala**	**0.001**	**-0.190**	**-55.41**	**17.34**	**-89.42**	**-21.40**	**-3.54**	**263**
L Caudate nucleus	0.046	-0.127	-72.36	36.15	-143.28	-1.44	-2.10	257
R Caudate nucleus	0.024	-0.140	-80.07	35.52	-149.76	-10.38	-2.28	261
L Globus pallidus	0.780	-0.017	-5.48	19.60	-43.93	32.97	-0.37	245
R Globus pallidus	0.172	-0.082	-23.96	17.52	-58.32	10.40	-1.60	255
**L Hippocampus**	**0.001**	**-0.213**	**-115.66**	**33.83**	**-182.03**	**-49.28**	**-2.96**	**255**
**R Hippocampus**	**0.001**	**-0.209**	**-115.61**	**33.75**	**-181.83**	**-49.40**	**-2.88**	**261**

L Lateral ventricle	0.013	0.168	1306.41	524.61	277.22	2335.60	9.02	263
R Lateral ventricle	0.057	0.128	935.76	491.25	-27.98	1899.50	6.90	263
L Nucleus accumbens	0.030	-0.125	-17.69	8.13	-33.64	-1.75	-3.75	262
R Nucleus accumbens	0.038	-0.123	-16.56	7.96	-32.18	-0.94	-3.40	257
**L Putamen**	**0.001**	**-0.205**	**-157.40**	**47.32**	**-250.24**	**-64.56**	**-3.19**	**247**
R Putamen	0.034	-0.125	-93.97	44.31	-180.89	-7.05	-2.00	254
L Thalamus	0.127	-0.085	-86.65	56.69	-197.88	24.58	-1.18	255
R Thalamus	0.014	-0.143	-118.31	48.12	-212.71	-23.91	-1.75	256

Results of cortical thickness analysis of PD patients with MoCA scores vs healthy controls. L – Left, R – Right, ROI – region of interest. Banks STS – Banks of the superior temporal sulcus. **Boldface** denotes significant results after Bonferroni correction

Supplementary Table 7i: Subcortical volume results - HY3 PD vs HY4&5 PD

ROI	*p*	*d*	*b*	SE	Lower CI	Upper CI	% diff	NPD
L Amygdala	0.468	-0.088	-20.24	27.85	-75.03	34.56	-1.489	83
R Amygdala	0.595	-0.061	-16.30	30.65	-76.61	44.01	-1.085	83
L Caudate nucleus	0.740	-0.040	-21.11	63.61	-146.25	104.03	-0.622	82
R Caudate nucleus	0.593	0.067	35.17	65.82	-94.33	164.67	1.025	81
L Globus pallidus	0.260	-0.115	-36.88	32.71	-101.25	27.49	-2.419	82
R Globus pallidus	0.272	-0.109	-34.04	30.95	-94.93	26.85	-2.271	81
L Hippocampus	0.033	-0.261	-123.69	57.69	-237.20	-10.19	-3.289	81
R Hippocampus	0.056	-0.227	-119.79	62.46	-242.67	3.09	-3.091	82
L Lateral ventricle	0.193	0.163	1308.16	1003.09	-665.26	3281.57	7.730	83
R Lateral ventricle	0.040	0.255	1888.33	914.68	88.85	3687.81	12.150	83
L Nucleus accumbens	0.328	-0.105	-14.74	15.05	-44.35	14.87	-3.274	82
R Nucleus accumbens	0.110	-0.186	-22.94	14.31	-51.09	5.21	-4.885	81
L Putamen	0.082	-0.209	-136.86	78.30	-290.95	17.23	-2.935	79
R Putamen	0.013	-0.286	-194.66	77.75	-347.63	-41.68	-4.335	81
L Thalamus	0.072	-0.191	-168.47	93.47	-352.38	15.45	-2.381	81
R Thalamus	0.213	-0.133	-103.14	82.58	-265.62	59.33	-1.570	83

Results of cortical thickness analysis of PD patients with MoCA scores vs healthy controls. L – Left, R – Right, ROI – region of interest. Banks STS – Banks of the superior temporal sulcus. **Boldface** denotes significant results after Bonferroni correction

Supplementary Table 8a: HY stage 1 analysis sample characteristics

		N		Mean age, years (SD)		% Female		DURILL, years (SD)	MoCA (SD)		No med/med
Site	Cohorts	HC	PD	HC	PD	HC	PD	PD	HC	PD	PD
Amsterdam	Amsterdam 1	34	0	55.3 (8.86)	NA	29	NA	NA	NA	NA	NA
	Amsterdam 2	0	6	NA	55.5 (7.56)	NA	50	5.5 (1.87)	NA	26.3 (1.97)	1/5
Bern	BE 1	19	1	56.3 (8.30)	69.0 (0)	26	0	7 (0)	NA	NA	0/1
	BE 2	17	0	67.4 (4.46)	NA	53	NA	NA	NA	NA	NA
Campinas	Campinas	105	23	57.3 (6.72)	60.8 (9.34)	54	35	4.2 (3.87)	NA	NA	0/23
Chang Gung	Chang Gung	180	78	60.7 (7.22)	57.3 (11.01)	47	55	4.2 (3.03)	NA	NA	NA
Christchurch	Christchurch	25	19	64.4 (8.61)	61.2 (7.07)	16	32	1.7 (2.54)	27.8 (1.37)	26.5 (2.46)	14/5

Donders	Donders	18	7	61.7 (8.81)	58.6 (6.80)	39	57	3.4 (2.23)	NA	NA	1/6
Graz	GRAZ 1	93	16	60.8 (9.93)	55.2 (11.04)	25	19	2 (1.22)	NA	NA	9/7
	GRAZ 2	0	4	NA	57.3 (12.16)	NA	25	2.7 (1.49)	NA	NA	2/2
Liege	Liege 1	27	4	66.0 (4.59)	62.8 (6.65)	41	50	2.8 (2.36)	NA	NA	2/2
	Liege 2	34	20	62.8 (7.09)	65.1 (7.22)	50	50	3.6 (1.99)	NA	NA	NA
Milan	Milan	7	0	51.6 (9.62)	NA	57	NA	NA	NA	NA	NA
NEUROCON	NEUROCON	8	2	62.6 (11.88)	52.5 (10.61)	63	0	NA	NA	NA	NA
NW-England	NW-England 1	14	4	67.6 (6.94)	66 (6.53)	50	75	3.1 (1.31)	28.1 (1.29)	28.8 (1.26)	0/4
	NW-England 2	10	2	64 (4.22)	64.5 (14.85)	30	0	4 (0)	28.7 (1.49)	27.5 (0.71)	0/2
ON Japan	ON Japan	9	0	62.1 (5.06)	NA	56	NA	NA	NA	NA	NA
Oxford	Oxford Discovery	39	27	64.4 (7.64)	60.4 (8.37)	36	41	1.8 (1.42)	28.1 (1.19)	27.4 (2.2)	5/22
Pennsylvania	UDALL/U19	5	6	67 (5.43)	65.5 (6.63)	40	17	3.7 (1.21)	NA	26.2 (1.94)	0/6
PPMI	PPMI 1	3	2	62 (6.22)	58.9 (8.77)	33	0	0.6 (0.42)	27.7 (1.15)	29 (0)	2/0
	PPMI 2	8	8	60.7 (8.31)	61.1 (9.45)	25	63	0.4 (0.26)	28.4 (1.3)	26.8 (2.76)	8/0
	PPMI 3	3	4	52.7 (10.86)	57.3 (10.47)	0	25	1.5 (1.14)	28 (1)	29 (1.15)	4/0
	PPMI 4	3	6	64 (10.42)	62.7 (12.14)	0	67	0.8 (0.51)	28 (1)	29 (0.89)	6/0
	PPMI 5	4	9	60.8 (7.60)	60.4 (13.34)	25	56	0.8 (0.74)	28 (0.82)	27.9 (1.17)	9/0
	PPMI 6	0	1	NA	66.8 (0)	NA	0	1.2 (0)	NA	24 (0)	1/0
	PPMI 7	8	9	61.9 (12.47)	58.1 (13.53)	25	22	0.5 (0.25)	28.6 (1.41)	29.1 (0.93)	9/0
	PPMI 8	3	10	61 (11.97)	64.5 (6.99)	0	40	0.5 (0.28)	27.7 (1.15)	27.5 (1.43)	10/0
	PPMI 9	7	4	58.5 (6.30)	58.9 (10.74)	43	25	0.9 (0.56)	28.3 (1.25)	27.8 (1.89)	4/0
	PPMI 10	9	16	62.7 (6.15)	63.6 (8.00)	56	31	0.3 (0.10)	27.9 (1.05)	26.1 (3.16)	16/0
	PPMI 11	6	1	50.1 (6.54)	41.5 (0)	67	100	0.1 (0)	29.7 (0.52)	30 (0)	1/0
	PPMI 12	7	5	46.9 (6.08)	54.8 (12.15)	43	40	0.3 (0.12)	29.3 (1.11)	28.6 (1.14)	5/0
	PPMI 13	0	4	NA	58.5 (14.94)	NA	25	0.2 (0.10)	NA	26 (2.16)	4/0
	PPMI 14	13	32	60.9 (9)	57.1 (11.24)	38	38	0.4 (0.35)	27.9 (0.86)	26.1 (2.83)	32/0
	PPMI 15	7	9	55.5 (5.18)	55.2 (3.73)	14	22	0.6 (0.52)	27.6 (0.79)	28.1 (1.27)	9/0
	PPMI 16	7	7	63.7 (10.16)	63 (5.74)	14	14	0.4 (0.28)	28.9 (1.21)	27.9 (1.35)	7/0
	PPMI 17	8	7	61.8 (11)	62.6 (8.58)	50	43	0.7 (0.58)	28.4 (0.92)	26.1 (3.08)	7/0
	PPMI 18	5	10	66 (4.79)	55.7 (9.98)	60	30	0.2 (0.08)	28.6 (0.89)	28.9 (1.2)	10/0
	PPMI 19	8	5	56.5 (13.8)	56.5 (6.12)	13	40	0.6 (1.00)	28 (0.93)	27.4 (1.67)	5/0
	PPMI 21	10	11	60.7 (4.73)	59.9 (10.36)	10	45	1.0 (0.96)	28.9 (1.1)	26.5 (2.02)	11/0
Rome SLF	Rome SLF	61	50	43.4 (9.52)	58 (9.39)	31	32	2.8 (1.95)	NA	NA	2/46
Stanford	Stanford	8	1	64.5 (5.09)	51.5 (0)	75	100	7.4 (0)	28.3 (1.49)	NA	0/1
Tao Wu	TaoWu	14	5	65.1 (5.95)	65.4 (5.18)	36	20	3 (1.58)	NA	NA	NA
**Total**	**41**	**846**	**435**	**59.5 (9.62)**	**59.3 (9.82)**	**39**	**40**	2.3 (2.50)	**28.2 (1.21)**	**27.2 (2.33)**	**196/131**

HY stage 1 sample demographics. PD – Parkinson's disease, HC – healthy controls, SD – standard deviation, DURILL – duration of illness, MoCA – Montreal Cognitive Assessment. Med/no med – medicated and non-medicated PD patients

Supplementary Table 8b: HY stage 2 analysis sample characteristics

		N		Mean age, years (SD)		% Female		DURILL, years (SD)	MoCA (SD)		No med/med
**Site**	**Cohorts**	**HC**	**PD**	**HC**	**PD**	**HC**	**PD**	**PD**	**HC**	**PD**	**PD**
Amsterdam	Amsterdam 1	32	0	58.9 (8.93)	NA	31	NA	NA	NA	NA	NA
	Amsterdam 2	0	46	NA	63.3 (6.59)	NA	39	5.7 (3.77)	NA	26.4 (2.26)	11/35
Bern	BE 1	15	13	57.8 (7.39)	61.2 (11.04)	20	38	12.9 (5.04)	NA	NA	0/13
	BE 2	26	0	68.3 (4.63)	NA	65	NA	NA	NA	NA	NA
Campinas	Campinas	104	40	60.3 (8.47)	59.6 (11.71)	57	35	7.3 (6.26)	NA	NA	0/40
Chang Gung	Chang Gung	180	85	61.9 (7.37)	61.5 (7.77)	43	36	8.6 (5.6)	NA	NA	NA
Christchurch	Christchurch	38	109	67.6 (8.62)	68.4 (7.43)	32	27	5.2 (5.27)	28 (1.41)	24.6 (3.12)	30/78

Donders	Donders	21	34	64.4 (8.83)	58.8 (9.65)	43	38	3.9 (2.70)	NA	NA	2/32
Graz	GRAZ 1	103	64	66 (8.79)	62.9 (9.38)	24	34	4.3 (3.56)	NA	NA	14/50
	GRAZ 2	0	14	NA	64.4 (10.35)	NA	21	4.9 (7.12)	NA	NA	1/13
Liege	Liege 1	27	15	66.8 (4.1)	65.1 (7.43)	37	33	8.5 (6.56)	NA	NA	1/14
	Liege 2	38	18	66 (7.82)	66.4 (8.08)	42	33	7.8 (4.5)	NA	NA	NA
Milan	Milan	4	19	62.5 (8.81)	57.7 (8.12)	50	37	12.2 (3.1)	NA	NA	NA
NEUROCON	NEUROCON	13	22	69.2 (10.44)	68.9 (9.62)	77	36	NA	NA	NA	NA
NW-England	NW-England 1	20	9	70.1 (7.38)	66.9 (8.27)	45	22	5.9 (4.28)	28.3 (1.33)	26.3 (4.77)	0/9
	NW-England 2	11	4	64.3 (3.95)	64.3 (4.99)	36	0	5.8 (3.86)	28.8 (1.47)	27.5 (3.54)	0/4
ON Japan	ON Japan	14	0	63.4 (5.43)	NA	50	NA	NA	NA	NA	NA
Oxford	Oxford Discovery	52	70	66.9 (6.77)	63.6 (10.19)	33	39	2.4 (1.57)	28.1 (1.29)	26.4 (2.81)	8/62
Pennsylvania	UDALL/U19	10	36	69.5 (5.82)	65.4 (7.93)	60	31	6.3 (4.17)	NA	26.2 (3.15)	0/6
PPMI	PPMI 1	5	12	68.9 (11.23)	62.7 (8.02)	40	50	0.6 (0.56)	27.6 (0.89)	26.7 (1.92)	12/0
	PPMI 2	11	7	63.7 (9.29)	64 (7.22)	27	71	1.1 (0.98)	28 (1.26)	25.9 (0.9)	7/0
	PPMI 3	2	13	64.5 (0.78)	61.2 (10.71)	50	31	0.6 (0.63)	27 (0)	27.2 (1.92)	13/0
	PPMI 4	5	4	69.5 (10.65)	72 (5.37)	0	50	1.1 (1.11)	27.6 (0.89)	27.5 (1)	4/0
	PPMI 5	7	4	66.2 (4.57)	67.2 (2.38)	43	0	0.8 (0.57)	27.7 (0.76)	26.8 (0.96)	4/0
	PPMI 6	1	1	80.9 (0)	55.8 (0)	0	0	1.4 (0)	26 (0)	26 (0)	1/0
	PPMI 7	7	1	66.2 (12.26)	57.9 (0)	14	0	1.3 (0)	28.4 (1.4)	30 (0)	1/0
	PPMI 8	4	2	62 (9.96)	70.1 (1.06)	0	50	0.5 (0.21)	27.5 (1)	29.5 (0.71)	2/0
	PPMI 9	6	12	62.2 (7.08)	64.5 (5.64)	17	42	0.6 (0.32)	28.2 (1.17)	28.5 (1.83)	12/0
	PPMI 10	7	5	67.6 (4.84)	64.7 (15.51)	43	40	0.4 (0.46)	28.1 (1.07)	27.2 (2.17)	5/0
	PPMI 11	4	4	58.4 (13.45)	63.6 (7.36)	25	75	0.2 (0)	29.5 (0.58)	26.8 (0.96)	4/0
	PPMI 12	8	14	54.4 (16.26)	59.6 (9.98)	13	29	0.6 (0.55)	29.1 (1.13)	28.7 (1.2)	14/0
	PPMI 13	0	2	NA	62 (9.55)	NA	0	0.3 (0.07)	NA	29 (1.41)	2/0
	PPMI 14	12	28	62.7 (8.25)	64.3 (7.3)	25	21	0.4 (0.36)	28.3 (0.87)	25.8 (2.01)	28/0
	PPMI 15	11	4	60.5 (8.74)	60 (10.73)	36	25	0.3 (0.24)	27.8 (0.87)	27.8 (2.06)	4/0
	PPMI 16	9	6	65 (9.38)	60.5 (10.63)	11	50	1.2 (0.88)	28.6 (1.24)	28.2 (1.72)	6/0
	PPMI 17	7	11	60.8 (11.48)	67.9 (9.11)	43	45	0.7 (0.39)	28.4 (0.98)	27.2 (1.6)	11/0
	PPMI 18	5	10	66 (4.79)	59.3 (8.47)	60	20	0.6 (0.71)	28.6 (0.89)	28.8 (1.32)	10/0
	PPMI 19	8	13	58.8 (12.04)	59.6 (10.3)	25	23	0.7 (0.57)	28 (0.93)	27.4 (1.61)	13/0
	PPMI 20	0	2	NA	67.6 (5.02)	NA	0	0.3 (0)	NA	24 (1.41)	2/0
	PPMI 21	10	7	60.7 (4.73)	63.8 (8.91)	10	43	0.6 (0.61)	28.9 (1.1)	25.9 (2.67)	7/0
Rome SLF	Rome SLF	42	138	42.7 (10.36)	63.3 (10.11)	10	41	5.4 (4.7)	NA	NA	0/137
Stanford	Stanford	10	30	65.6 (6.82)	66.7 (7.36)	80	50	5.4 (3.14)	27.9 (1.66)	25.1 (4.72)	0/30
Tao Wu	TaoWu	18	12	65.2 (5.7)	65 (4.65)	33	58	5.4 (3.78)	NA	NA	NA
**Total**	**42**	907	940	62.9 (9.66)	63.7 (9.21)	38	36	4.9 (4.97)	28.2 (1.25)	26.2 (2.99)	**229/493**

HY stage 2 sample demographics. PD – Parkinson's disease, HC – healthy controls, SD – standard deviation, DURILL – duration of illness, MoCA – Montreal Cognitive Assessment. Med/no med – medicated and non-medicated PD patients

Supplementary Table 8c: HY stage 3 analysis sample characteristics

		N		Mean age, years (SD)		% Female		DURILL, years (SD)	MoCA (SD)		No med/med
**Site**	**Cohorts**	**HC**	**PD**	**HC**	**PD**	**HC**	**PD**	**PD**	**HC**	**PD**	**PD**
Amsterdam	Amsterdam 1	21	0	57.9 (8.71)	NA	24	NA	NA	NA	NA	NA
	Amsterdam 2	0	5	NA	60.2 (9.09)	NA	60	3.4 (3.21)	NA	26 (1.73)	2/3
Bern	BE 1	11	10	56.1 (10.22)	66 (11.01)	36	70	12.9 (3.63)	NA	NA	0/10
	BE 2	17	2	69.1 (5.29)	57.5 (7.78)	59	100	7.0 (1.41)	NA	NA	0/2
Campinas	Campinas	58	20	61.6 (9.37)	58.6 (11.28)	53	40	10.3 (6.82)	NA	NA	0/20
Chang Gung	Chang Gung	115	54	63.0 (8.13)	59.4 (9.84)	48	41	13.2 (7.07)	NA	NA	NA

Christchurch	Christchurch	17	43	71.9 (7.39)	72.4 (7.67)	24	30	6.7 (5.38)	27.6 (1.32)	22.2 (4.28)	4/39
Donders	Donders	15	14	65.6 (10.1)	66.5 (12.06)	40	64	6.4 (5.97)	NA	NA	0/14
Graz	GRAZ 1	52	5	68.3 (9.36)	71.5 (3.35)	25	40	10.1 (6.66)	NA	NA	0/5
Liege	Liege 1	20	4	66.6 (4.21)	70.0 (2.94)	45	75	9.8 (0.96)	NA	NA	0/4
	Liege 2	20	3	69.0 (8.4)	80.0 (7.55)	30	67	7.3 (2.89)	NA	NA	NA
Milan	Milan	3	4	65.0 (8.89)	55.8 (8.26)	33	50	10.5 (3)	NA	NA	0/4
NEUROCON	NEUROCON	12	0	70.3 (9.96)	NA	75	NA	NA	NA	NA	NA
NW-England	NW-England 1	11	11	73.4 (5.82)	70.7 (10.12)	36	9	7.2 (3.54)	28.5 (1.21)	24.3 (4.08)	0/11
	NW-England 2	6	1	63.7 (4.5)	72 (0)	33	100	10 (0)	28.7 (1.63)	24 (0)	0/1
ON Japan	ON Japan	8	0	64.9 (6.38)	NA	38	NA	NA	NA	NA	NA
Oxford	Oxford Discovery	21	2	72.4 (4.77)	71.1 (7.57)	29	50	2.1 (2.3)	28.2 (1.42)	27.5 (0.71)	0/2
Pennsylvania	UDALL/U19	7	60	71.9 (5.3)	66.4 (8.12)	57	37	8.1 (5.86)	NA	25.7 (2.7)	0/60
PPMI	PPMI 1	2	0	80.6 (1.84)	NA	100	NA	NA	27.5 (0.71)	NA	NA
	PPMI 2	3	0	73.5 (5.47)	NA	0	NA	NA	28 (1.73)	NA	NA
	PPMI 4	3	0	77.3 (1.7)	NA	0	NA	NA	27.7 (1.15)	NA	NA
	PPMI 5	3	0	68.2 (3.14)	NA	100	NA	NA	28 (1)	NA	NA
	PPMI 6	1	0	80.9 (0)	NA	0	NA	NA	26 (0)	NA	NA
	PPMI 7	4	0	68.9 (18.13)	NA	75	NA	NA	29.5 (0.58)	NA	NA
	PPMI 8	2	0	65.4 (13.22)	NA	0	NA	NA	27 (0)	NA	NA
	PPMI 9	2	0	63.3 (10.32)	NA	0	NA	NA	29.5 (0.71)	NA	NA
	PPMI 10	3	0	70 (3.23)	NA	33	NA	NA	28.3 (1.53)	NA	NA
	PPMI 11	4	0	58 (13.77)	NA	25	NA	NA	29.5 (0.58)	NA	NA
	PPMI 12	2	0	72.4 (9.12)	NA	0	NA	NA	29.5 (0.71)	NA	NA
	PPMI 14	3	0	57.7 (16.76)	NA	33	NA	NA	28.3 (1.15)	NA	NA
	PPMI 15	2	0	74.6 (3.25)	NA	50	NA	NA	28 (1.41)	NA	NA
	PPMI 16	3	0	73.3 (0.38)	NA	33	NA	NA	29 (1)	NA	NA
	PPMI 17	4	0	67.5 (9.46)	NA	25	NA	NA	28.3 (0.96)	NA	NA
	PPMI 18	2	0	71.1 (1.27)	NA	50	NA	NA	28 (0)	NA	NA
	PPMI 19	4	0	56.3 (16.4)	NA	0	NA	NA	28.5 (0.58)	NA	NA
	PPMI 21	2	2	64.3 (2.76)	61.5 (8.77)	0	50	0.3 (0.14)	28.5 (0.71)	26 (1.41)	2/0
Rome SLF	Rome SLF	24	9	48.4 (8.32)	67.1 (10.87)	29	33	9.4 (3.09)	NA	NA	0/9
Stanford	Stanford	5	8	68.4 (8.89)	73.3 (8.68)	100	75	7.4 (4.61)	28.2 (2.05)	20.3 (4.4)	NA
Tao Wu	TaoWu	9	1	68.1 (5.62)	62 (0)	22	100	6 (0)	NA	NA	NA
**Total**	**39**	**501**	**258**	**64.9 (9.98)**	**65.7 (10.41)**	**40**	**42**	**9 (6.24)**	**28.3 (1.3)**	**24.1 (3.91)**	**8/184**

HY stage 3 sample demographics. PD – Parkinson's disease, HC – healthy controls, SD – standard deviation, DURILL – duration of illness, MoCA – Montreal Cognitive Assessment. Med/no med – medicated and non-medicated PD patients

Supplementary Table 8d: HY stage 4&5 analysis sample characteristics

		N		Mean age, years (SD)		% Female		DURILL, years (SD)	MoCA (SD)		No med/med
**Site**	**Cohorts**	**HC**	**PD**	**HC**	**PD**	**HC**	**PD**	**PD**	**HC**	**PD**	**PD**
Amsterdam	Amsterdam 1	17	0	60.7 (9.26)	NA	47	NA	NA	NA	NA	NA
Bern	BE 1	3	4	53.3 (8.08)	65.3 (5.91)	67	75	13.8 (1.26)	NA	NA	0/4
	BE 2	13	1	69.6 (4.33)	64 (0)	69	0	20 (0)	NA	NA	0/1
Campinas	UNICAMP	31	13	65.1 (9.59)	62.2 (8.22)	68	31	13 (5.4)	NA	NA	0/13
Chang Gung	Chang Gung	79	34	63.4 (7.71)	63.5 (9.88)	57	62	14.9 (5.19)	NA	NA	NA
Christchurch	Christchurch	13	17	74.7 (3.7)	74.3 (5.74)	31	24	11.7 (6.96)	27.7 (1.55)	17.7 (5.38)	0/17
Donders	Donders	10	0	65.7 (11.31)	NA	50	NA	NA	NA	NA	NA
Graz	PROMOVE/ASPS I	32	2	71.8 (7.95)	64.3 (10.82)	19	0	13.9 (11.73)	NA	NA	2/0
Liege	Liege 1	11	0	68.0 (4)	NA	27	NA	NA	NA	NA	NA

	Liege 2	13	0	71.9 (6.84)	NA	38	NA	NA	NA	NA	NA
Milan	Milan	5	0	57.2 (12.83)	NA	80	NA	NA	NA	NA	NA
NEUROCON	NEUROCON	6	0	74.7 (6.5)	NA	67	NA	NA	NA	NA	NA
NW-England	NW-England 1	12	5	72.0 (4.41)	72.4 (7.5)	33	0	11.2 (4.82)	28.7 (1.23)	22.6 (3.13)	0/5
	NW-England 2	6	1	64.8 (5.42)	61 (0)	17	100	18 (0)	28.2 (1.6)	24 (0)	0/1
ON Japan	ON Japan	8	0	64.1 (4.55)	NA	63	NA	NA	NA	NA	NA
Oxford	Oxford Discovery	10	2	73.8 (5.03)	68.7 (4.55)	50	50	5.9 (0.32)	28 (1.49)	23.5 (2.12)	0/2
Pennsylvania	UDALL/U19	3	2	72.7 (5.51)	75 (8.49)	67	0	15.5 (2.12)	NA	14.5 (0.71)	0/2
PPMI	PPMI 1	2	0	80.6 (1.84)	NA	100	NA	NA	27.5 (0.71)	NA	NA
	PPMI 2	4	0	71.5 (5.79)	NA	25	NA	NA	29 (1.41)	NA	NA
	PPMI 4	1	0	79.2 (0)	NA	0	NA	NA	27 (0)	NA	NA
	PPMI 5	3	0	70.0 (0.4)	NA	67	NA	NA	27.7 (1.15)	NA	NA
	PPMI 6	1	0	80.9 (0)	NA	0	NA	NA	26 (0)	NA	NA
	PPMI 7	2	0	76.9 (5.09)	NA	50	NA	NA	28 (1.41)	NA	NA
	PPMI 8	1	0	56 (0)	NA	0	NA	NA	27 (0)	NA	NA
	PPMI 9	2	0	60.7 (6.65)	NA	0	NA	NA	29 (1.41)	NA	NA
	PPMI 10	3	0	70.7 (4.62)	NA	33	NA	NA	27.7 (1.15)	NA	NA
	PPMI 11	1	0	76.8 (0)	NA	0	NA	NA	29 (0)	NA	NA
	PPMI 12	4	0	66.3 (12.73)	NA	0	NA	NA	29.3 (0.96)	NA	NA
	PPMI 14	1	0	68.1 (0)	NA	100	NA	NA	28 (0)	NA	NA
	PPMI 15	2	0	74.6 (3.25)	NA	50	NA	NA	28 (1.41)	NA	NA
	PPMI 16	4	0	72.5 (1.74)	NA	25	NA	NA	28.8 (0.96)	NA	NA
	PPMI 17	1	0	71.5 (0)	NA	0	NA	NA	28 (0)	NA	NA
	PPMI 18	1	0	72 (0)	NA	0	NA	NA	28 (0)	NA	NA
	PPMI 19	2	0	73.9 (2.19)	NA	0	NA	NA	28.5 (0.71)	NA	NA
	PPMI 21	1	0	61 (0)	NA	0	NA	NA	27 (0)	NA	NA
Rome SLF	Rome SLF	8	0	46.1 (9.06)	NA	38	NA	NA	NA	NA	NA
Stanford	Stanford	3	2	74.4 (4.37)	73.9 (6.79)	100	0	4.3 (0.49)	28.3 (2.08)	15.5 (2.12)	0/2
Tao Wu	TaoWu	10	0	65.1 (5.04)	NA	40	NA	NA	NA	NA	NA
**Total**	**38**	**329**	**83**	**66.8 (9.22)**	**66.8 (9.38)**	**47**	**41**	**13.3 (5.8)**	**28.2 (1.33)**	**18.8 (5.12)**	**2/45**

HY stages 4/5 sample demographics. PD – Parkinson's disease, HC – healthy controls, SD – standard deviation, DURILL – duration of illness, MoCA – Montreal Cognitive Assessment. Med/no med – medicated and non-medicated PD patients

Supplementary Table 9: Overlap controls in HY stage analyses

HY stage	1	2	3
2	672	.	.
3	359	437	.
4-5	211	294	209

Amount of control subjects that overlapped across HY stage analyses.

Supplementary Table 10a: Group differences on DURILL across HY stages

					Z			*p*		
**HY stage**	**N**	**Mean (SD)**	**Median**	**IQR**	**HY 1**	**HY 2**	**HY 3**	**HY 1**	**HY 2**	**HY 3**

1	436	2.27 (2.50)	1.2	2.6	NA	NA	NA	NA	NA	NA
2	1039	4.88 (4.90)	3.2	6	-10.4	NA	NA	**< 0.001**	NA	NA
3	264	9.01 (6.29)	8	8	-16.5	-11	NA	**< 0.001**	**< 0.001**	NA
4-5	83	13.6 (5.80)	14	7.1	-13.5	-11.4	-5.8	**< 0.001**	**< 0.001**	**< 0.001**

Mann-Whitney tests for group differences across HY stages. Abbreviations: DURILL, duration of illness.

Supplementary Table 10b: Group differences on MoCA across HY stages

					**Z**			***p***		
**HY stage**	**N**	**Mean (SD)**	**Median**	**IQR**	**HY 1**	**HY 2**	**HY 3**	**HY 1**	**HY 2**	**HY 3**
1	228	27.2 (2.32)	28	3	NA	NA	NA	NA	NA	NA
2	525	26.0 (3.04)	27	4	-4.5	NA	NA	**< 0.001**	NA	NA
3	135	24.2 (3.93)	25	5	-7.4	-4.6	NA	**< 0.001**	**< 0.001**	NA
4-5	31	19.2 (5.23)	19	9.5	-7.7	-6.8	-4.5	**< 0.001**	**< 0.001**	**< 0.001**

Mann-Whitney tests for group differences across HY stages. Abbreviations: MoCA, Montreal Cognitive Assessment.

Supplementary Table 11a: Cortical thickness results - Group differences corrected for DURILL across HY stages

	HY1 vs HY2		HY1 vs HY3		HY1 vs HY4-5		HY2 vs HY3		HY2 vs HY4		HY3 vs HY4	
	p	d	p	d	p	d	p	d	*p*	*d*	*p*	*d*
L Banks STS	0.451	0.037	0.020	-0.169	0.142	-0.167	0.011	-0.149	0.012	-0.244	0.028	-0.230
R Banks STS	0.987	0.001	0.042	-0.136	0.206	-0.134	0.019	-0.136	0.140	-0.144	0.561	-0.058
L Caudal anterior cingulate	0.945	0.003	0.837	0.014	0.261	0.119	0.323	-0.063	0.690	0.042	0.668	0.048
R Caudal anterior cingulate	0.623	-0.025	0.774	0.020	0.257	-0.120	0.543	-0.038	0.037	-0.216	0.085	-0.195
L Caudal middle frontal	0.443	0.038	0.081	-0.120	0.053	-0.214	0.089	-0.102	0.068	-0.184	0.160	-0.143
R Caudal middle frontal	0.719	-0.018	0.008	-0.183	0.142	-0.160	0.063	-0.116	0.726	-0.037	0.927	0.009
L Cuneus	0.523	-0.032	0.201	-0.087	0.982	0.002	0.069	-0.114	0.713	0.038	0.418	0.089
R Cuneus	0.529	-0.029	0.046	-0.118	0.451	-0.071	0.047	-0.112	0.995	0.001	0.697	0.034
L Entorhinal	0.638	-0.024	0.009	-0.184	0.102	-0.175	0.009	-0.162	0.027	-0.228	0.958	0.006
R Entorhinal	0.881	-0.006	0.062	-0.113	0.089	-0.151	0.050	-0.104	0.124	-0.131	0.809	-0.021
L Frontal pole	0.673	-0.022	0.450	-0.056	0.121	-0.175	0.451	-0.049	0.284	-0.115	0.296	-0.128
R Frontal pole	0.200	-0.065	0.172	-0.097	0.065	-0.200	0.358	-0.057	0.005	-0.288	0.002	-0.329
L Fusiform	0.742	-0.016	0.002	-0.197	0.013	-0.249	0.003	-0.175	0.001	-0.326	0.094	-0.160
R Fusiform	0.614	-0.020	0.006	-0.152	0.067	-0.160	0.013	-0.121	0.008	-0.216	0.033	-0.167
L Inferior parietal	0.851	-0.009	0.001	-0.228	0.261	-0.122	**<0.001**	**-0.215**	0.127	-0.153	0.450	-0.075

R Inferior parietal	0.704	0.017	0.084	-0.110	0.141	-0.149	0.015	-0.138	0.027	-0.212	0.194	-0.124
L Inferior temporal	0.789	0.013	0.049	-0.134	0.481	-0.073	0.004	-0.178	0.005	-0.280	0.244	-0.124
R Inferior temporal	0.863	-0.008	0.034	-0.129	0.158	-0.134	0.215	-0.070	0.032	-0.203	0.041	-0.183
L Insula	0.650	-0.024	0.153	-0.105	0.205	-0.142	0.898	-0.008	0.405	-0.090	0.103	-0.193
R Insula	0.349	-0.044	0.345	-0.061	0.221	-0.118	0.903	0.007	0.060	-0.174	0.012	-0.232
L Isthmus cingulate	0.462	-0.038	0.041	-0.145	0.126	-0.169	0.022	-0.144	0.021	-0.241	0.124	-0.172
R Isthmus cingulate	0.778	-0.014	0.438	-0.052	0.751	-0.034	0.033	-0.130	0.065	-0.188	0.538	-0.066
L Lateral occipital	0.472	-0.036	0.124	-0.105	0.329	-0.106	0.027	-0.134	0.847	-0.020	0.252	0.116
R Lateral occipital	0.481	-0.035	0.283	-0.073	0.559	-0.063	0.028	-0.132	0.211	-0.126	0.616	-0.054
L Lateral orbitofrontal	0.850	0.009	0.073	-0.117	0.227	-0.123	0.015	-0.141	0.028	-0.210	0.153	-0.147
R Lateral orbitofrontal	0.260	-0.048	0.094	-0.098	0.007	-0.246	0.296	-0.055	0.003	-0.257	0.001	-0.266
L Lingual	0.244	-0.056	0.003	-0.204	0.092	-0.175	0.002	-0.185	0.060	-0.183	0.563	-0.057
R Lingual	0.159	-0.060	0.012	-0.145	0.174	-0.122	0.049	-0.104	0.636	-0.042	0.649	-0.040
L Medial orbitofrontal	0.965	0.002	0.985	0.001	0.102	-0.173	0.958	-0.003	0.086	-0.180	0.023	-0.268
R Medial orbitofrontal	0.664	-0.020	0.929	-0.005	0.043	-0.195	0.984	-0.001	0.005	-0.258	**0.001**	**-0.305**
L Middle temporal	0.494	-0.034	**<0.001**	**-0.254**	0.051	-0.203	**<0.001**	**-0.222**	0.001	-0.330	0.097	-0.176
R Middle temporal	0.758	-0.014	0.007	-0.168	0.041	-0.197	0.008	-0.150	**<0.001**	**-0.334**	0.009	-0.238
L Paracentral	0.808	-0.012	0.025	-0.145	0.659	-0.045	0.045	-0.118	0.888	-0.014	0.828	0.021
R Paracentral	0.732	-0.016	0.017	-0.150	0.867	-0.017	0.097	-0.098	0.994	-0.001	0.981	0.002
L Parahippocampal	0.175	-0.069	0.018	-0.168	0.180	-0.145	0.172	-0.085	0.189	-0.136	0.735	-0.038
R Parahippocampal	0.494	-0.033	0.003	-0.198	0.031	-0.226	0.001	-0.195	0.009	-0.261	0.635	-0.050
L Pars opercularis	0.856	0.009	0.033	-0.139	0.122	-0.163	0.088	-0.100	0.038	-0.205	0.032	-0.219
R Pars opercularis	0.694	0.018	0.437	-0.050	0.766	-0.031	0.408	-0.048	0.668	-0.042	0.271	-0.106
L Pars orbitalis	0.546	-0.030	0.048	-0.139	0.023	-0.251	0.211	-0.076	0.024	-0.232	0.037	-0.230
R Pars orbitalis	0.138	-0.073	0.937	-0.005	0.431	-0.083	0.704	0.023	0.128	-0.154	0.008	-0.290
L Pars triangularis	0.351	0.044	0.032	-0.141	0.401	-0.089	0.017	-0.142	0.373	-0.089	0.974	-0.003
R Pars triangularis	0.883	0.007	0.149	-0.093	0.423	-0.083	0.512	-0.039	0.569	-0.057	0.276	-0.110
L Pericalcarine	0.017	-0.109	0.477	-0.048	0.996	-0.001	0.373	-0.052	0.628	-0.045	0.736	-0.038
R Pericalcarine	0.216	-0.052	0.004	-0.166	0.141	-0.130	0.089	-0.092	0.535	0.055	0.778	0.025
L Postcentral	0.903	0.006	0.200	-0.081	0.060	-0.191	0.155	-0.080	0.031	-0.205	0.045	-0.196
R Postcentral	0.868	-0.007	0.178	-0.080	0.317	-0.091	0.204	-0.067	0.201	-0.111	0.194	-0.109
L Posterior cingulate	0.860	-0.008	0.083	-0.112	0.241	-0.119	0.107	-0.098	0.127	-0.157	0.376	-0.099
R Posterior cingulate	0.465	-0.033	0.053	-0.116	0.183	-0.124	0.008	-0.147	0.008	-0.247	0.183	-0.120
L Precentral	0.388	0.042	0.021	-0.152	0.144	-0.152	0.025	-0.131	0.072	-0.177	0.182	-0.125
R Precentral	0.937	0.004	0.047	-0.136	0.472	-0.077	0.048	-0.116	0.480	-0.069	0.952	-0.006
L Precuneus	0.738	-0.015	0.002	-0.202	0.107	-0.163	0.001	-0.194	0.126	-0.147	0.582	-0.054
R Precuneus	0.533	-0.029	**0.001**	**-0.216**	0.047	-0.203	0.004	-0.166	0.183	-0.129	0.545	-0.057
L Rostral anterior cingulate	0.362	0.046	0.950	-0.004	0.393	-0.088	0.382	-0.058	0.133	-0.164	0.150	-0.166
R Rostral anterior cingulate	0.793	0.013	0.342	0.061	0.159	0.140	0.030	0.129	0.524	0.063	0.151	-0.141
L Rostral middle frontal	0.460	0.033	0.037	-0.129	0.443	-0.073	0.001	-0.186	0.069	-0.169	0.628	-0.047
R Rostral middle frontal	0.490	-0.028	0.047	-0.114	0.041	-0.179	0.209	-0.064	0.009	-0.218	0.002	-0.278
L Superior frontal	0.801	0.012	0.037	-0.141	0.370	-0.095	0.023	-0.141	0.244	-0.120	0.985	-0.002
R Superior frontal	0.642	-0.021	0.010	-0.163	0.473	-0.072	0.063	-0.105	0.832	-0.020	0.945	-0.006
L Superior parietal	0.369	-0.042	0.008	-0.176	0.265	-0.112	0.017	-0.135	0.526	-0.058	0.828	-0.021
R Superior parietal	0.884	-0.006	0.042	-0.119	0.583	-0.051	0.024	-0.122	0.545	-0.054	0.759	-0.026
L Superior temporal	0.920	-0.005	**<0.001**	**-0.276**	0.092	-0.187	0.001	-0.206	0.009	-0.257	0.131	-0.168
R Superior temporal	0.245	-0.049	**0.001**	**-0.201**	0.043	-0.186	0.022	-0.115	0.034	-0.178	0.081	-0.144
L Supramarginal	0.569	-0.028	**<0.001**	**-0.245**	0.178	-0.145	0.001	-0.200	0.018	-0.231	0.249	-0.111
R Supramarginal	0.364	-0.044	0.002	-0.200	0.002	-0.328	0.006	-0.165	**0.001**	**-0.337**	0.018	-0.229
L Temporal pole	0.322	-0.053	0.307	-0.076	0.017	-0.269	0.919	0.007	0.075	-0.193	0.066	-0.229

R Temporal pole	0.154	-0.069	0.073	-0.120	0.113	-0.160	0.259	-0.067	0.025	-0.218	0.133	-0.143
L Transverse temporal	0.401	-0.044	0.075	-0.125	0.026	-0.252	0.651	-0.028	0.264	-0.117	0.044	-0.202
R Transverse temporal	0.161	-0.054	0.003	-0.157	0.173	-0.114	0.083	-0.082	0.525	-0.050	0.513	-0.045

Supplementary Table 11b: Cortical surface area results - Group differences corrected for DURILL across HY stages

	HY1 vs HY2		HY1 vs HY3		HY1 vs HY4-5		HY2 vs HY3		HY2 vs HY4		HY3 vs HY4	
	p	*d*	*p*	*d*	*p*	*d*	*p*	*d*	*p*	*d*	*p*	*d*
L Banks STS	0.034	0.125	0.085	-0.138	0.375	-0.110	0.009	-0.185	0.304	-0.120	0.685	0.049
R Banks STS	0.034	0.115	0.186	-0.102	0.547	-0.071	0.006	-0.183	0.141	-0.160	0.655	-0.056
L Caudal anterior cingulate	0.252	0.063	0.974	0.002	0.769	-0.034	0.051	-0.130	0.061	-0.208	0.579	-0.071
R Caudal anterior cingulate	0.748	0.018	0.725	-0.027	0.284	-0.127	0.822	-0.015	0.602	0.057	0.892	-0.017
L Caudal middle frontal	0.228	-0.066	0.391	-0.064	0.780	-0.032	0.177	-0.089	0.867	0.018	0.056	0.233
R Caudal middle frontal	0.567	-0.031	0.189	-0.102	0.611	-0.060	0.056	-0.128	0.996	0.001	0.092	0.211
L Cuneus	0.446	-0.044	0.562	-0.048	0.492	0.085	0.785	-0.020	0.613	0.059	0.082	0.236
R Cuneus	0.694	0.023	0.237	-0.096	0.473	-0.089	0.041	-0.145	0.333	-0.114	0.347	0.125
L Entorhinal	0.729	0.019	0.649	-0.034	0.256	-0.132	0.620	-0.033	0.212	-0.138	0.482	-0.082
R Entorhinal	0.423	0.045	0.806	-0.019	0.619	-0.059	0.066	-0.124	0.429	-0.087	0.749	0.040
L Frontal pole	0.521	0.032	0.026	-0.153	0.816	-0.024	0.006	-0.166	0.901	0.012	0.074	0.182
R Frontal pole	0.060	0.099	0.430	0.055	0.350	0.104	0.138	-0.091	0.952	0.006	0.378	0.099
L Fusiform	0.797	0.014	0.003	-0.225	0.013	-0.293	0.009	-0.170	0.147	-0.154	0.728	0.039
R Fusiform	0.899	-0.007	0.007	-0.206	0.016	-0.281	0.020	-0.153	0.112	-0.171	0.748	-0.040
L Inferior parietal	0.839	0.011	0.038	-0.162	0.028	-0.262	0.009	-0.171	0.094	-0.178	0.917	-0.013
R Inferior parietal	0.642	-0.026	0.089	-0.130	0.343	-0.110	0.014	-0.162	0.066	-0.199	0.934	0.010
L Inferior temporal	0.518	-0.035	0.018	-0.186	0.026	-0.260	0.108	-0.107	0.047	-0.211	0.596	-0.064
R Inferior temporal	0.883	-0.008	0.006	-0.216	0.246	-0.137	0.015	-0.161	0.154	-0.153	0.618	0.062
L Insula	0.946	-0.003	0.817	-0.017	0.362	-0.101	0.263	-0.068	0.054	-0.190	0.974	0.004
R Insula	0.821	0.012	0.304	-0.075	0.413	-0.093	0.059	-0.119	0.029	-0.227	0.723	-0.041
L Isthmus cingulate	0.267	-0.060	0.552	-0.044	0.177	-0.159	0.714	-0.024	0.943	-0.008	0.399	0.103
R Isthmus cingulate	0.545	-0.033	0.657	-0.032	0.750	-0.037	0.578	-0.036	0.262	0.122	0.052	0.226
L Lateral occipital	0.310	-0.054	0.024	-0.169	0.024	-0.262	0.002	-0.205	0.093	-0.185	0.498	0.087
R Lateral occipital	0.370	-0.049	0.042	-0.154	0.109	-0.185	0.032	-0.143	0.542	-0.068	0.215	0.157
L Lateral orbitofrontal	0.425	-0.044	0.331	-0.076	0.056	-0.233	0.618	-0.033	0.088	-0.187	0.367	-0.112
R Lateral orbitofrontal	0.081	-0.094	0.047	-0.151	0.184	-0.157	0.274	-0.071	0.205	-0.136	0.938	-0.009
L Lingual	**0.001**	**-0.188**	0.001	-0.257	0.210	-0.148	0.757	-0.021	0.955	0.006	0.542	0.081
R Lingual	0.024	-0.124	0.012	-0.192	0.273	-0.129	0.153	-0.095	0.864	0.019	0.220	0.160
L Medial orbitofrontal	0.528	0.034	0.046	-0.148	0.053	-0.222	0.005	-0.182	0.064	-0.199	0.919	0.012
R Medial orbitofrontal	0.917	-0.006	0.279	-0.080	0.323	-0.112	0.283	-0.070	0.883	0.016	0.044	0.239
L Middle temporal	0.542	0.034	0.273	-0.087	0.725	-0.042	0.066	-0.123	0.693	-0.043	0.361	0.116
R Middle temporal	0.320	0.054	**0.001**	**-0.262**	0.013	-0.291	**<0.001**	**-0.243**	0.043	-0.215	0.313	0.122
L Paracentral	0.824	0.012	0.860	0.013	0.909	0.013	0.847	-0.013	0.551	-0.065	0.766	0.035
R Paracentral	0.861	0.010	0.915	0.008	0.604	-0.060	0.942	-0.005	0.509	-0.071	0.475	0.086
L Parahippocampal	0.777	0.015	0.107	-0.124	0.318	-0.118	0.051	-0.126	0.692	-0.042	0.323	0.124
R Parahippocampal	0.223	0.065	0.075	-0.139	0.503	0.079	0.006	-0.179	0.923	0.010	0.348	0.120
L Pars opercularis	0.574	-0.031	0.093	-0.128	0.112	-0.190	0.116	-0.104	0.382	-0.096	0.776	0.035
R Pars opercularis	0.013	-0.139	0.001	-0.253	0.016	-0.291	0.134	-0.101	0.046	-0.223	0.244	-0.147
L Pars orbitalis	0.541	-0.033	0.305	-0.077	0.225	-0.140	0.954	-0.004	0.240	-0.124	0.607	-0.060

R Pars orbitalis	0.993	<0.001	0.094	-0.127	0.051	-0.229	0.029	-0.143	0.029	-0.236	0.778	-0.034
L Pars triangularis	0.282	-0.060	0.347	-0.072	0.088	-0.203	0.860	0.012	0.681	-0.046	0.387	-0.109
R Pars triangularis	0.317	-0.055	0.120	-0.119	0.147	-0.169	0.609	-0.034	0.500	-0.074	0.519	-0.079
L Pericalcarine	0.072	-0.102	0.140	-0.120	0.726	0.043	0.557	0.042	0.187	0.153	0.364	0.124
R Pericalcarine	0.030	-0.124	0.130	-0.121	0.657	-0.055	0.594	0.038	0.927	0.011	0.583	0.076
L Postcentral	0.959	-0.003	0.049	-0.156	0.033	-0.257	0.001	-0.219	0.050	-0.214	0.531	0.080
R Postcentral	0.214	0.071	0.345	-0.074	0.043	-0.241	0.068	-0.126	0.011	-0.285	0.982	0.003
L Posterior cingulate	0.632	0.026	0.599	-0.040	0.122	-0.180	0.202	-0.085	0.157	-0.155	0.753	-0.040
R Posterior cingulate	0.603	0.027	0.893	-0.010	0.074	-0.208	0.310	-0.065	0.032	-0.230	0.558	-0.075
L Precentral	0.548	0.032	0.468	-0.055	0.643	-0.054	0.048	-0.129	0.762	-0.032	0.153	0.173
R Precentral	0.856	0.010	0.109	-0.125	0.869	-0.019	0.006	-0.176	0.783	-0.029	0.089	0.207
L Precuneus	0.481	0.038	0.159	-0.108	0.051	-0.229	0.008	-0.176	0.013	-0.269	0.993	0.001
R Precuneus	0.220	0.066	0.355	-0.070	0.351	-0.108	0.032	-0.140	0.040	-0.222	0.802	-0.032
L Rostral anterior cingulate	0.152	0.079	0.463	0.055	0.339	0.107	0.738	0.022	0.646	-0.051	0.720	-0.045
R Rostral anterior cingulate	0.744	0.017	0.488	-0.052	0.182	-0.154	0.422	-0.052	0.906	0.013	0.940	-0.009
L Rostral middle frontal	0.122	-0.082	0.110	-0.120	0.597	-0.061	0.097	-0.104	0.732	-0.035	0.236	0.140
R Rostral middle frontal	0.479	-0.037	0.566	-0.043	0.583	-0.063	0.180	-0.085	0.844	-0.021	0.089	0.203
L Superior frontal	0.107	-0.083	0.211	-0.093	0.471	-0.082	0.416	-0.051	0.874	-0.016	0.553	0.071
R Superior frontal	0.443	-0.041	0.143	-0.110	0.531	-0.071	0.120	-0.100	0.409	-0.086	0.347	0.114
L Superior parietal	0.237	-0.065	0.029	-0.170	0.415	-0.094	0.038	-0.138	0.309	-0.109	0.191	0.167
R Superior parietal	0.256	-0.062	0.196	-0.099	0.035	-0.244	0.065	-0.122	0.031	-0.232	0.821	0.028
L Superior temporal	0.409	0.046	0.952	0.005	0.366	-0.105	0.390	-0.059	0.260	-0.121	0.986	-0.002
R Superior temporal	0.028	0.118	0.626	-0.037	0.269	-0.127	0.005	-0.186	0.093	-0.180	0.609	0.064
L Supramarginal	0.872	-0.009	0.258	-0.088	0.005	-0.327	0.014	-0.163	0.003	-0.314	0.933	0.011
R Supramarginal	0.190	0.072	0.591	-0.042	0.349	-0.110	0.012	-0.168	0.018	-0.256	0.750	-0.041
L Temporal pole	0.637	-0.026	0.096	-0.131	0.681	-0.050	0.335	-0.066	0.539	0.069	0.423	0.102
R Temporal pole	0.681	-0.023	0.825	0.016	0.679	0.048	0.658	0.030	0.294	0.116	0.424	0.097
L Transverse temporal	0.707	0.020	0.514	-0.049	0.979	0.003	0.852	-0.012	0.970	-0.004	0.393	0.107
R Transverse temporal	0.863	0.009	0.045	-0.155	0.045	-0.239	0.425	-0.052	0.479	-0.076	0.999	<0.001

Supplementary Table 11c: Subcortical volume results - Group differences corrected for DURILL across HY stages

	HY1 vs HY2		HY1 vs HY3		HY1 vs HY4-5		HY2 vs HY3		HY2 vs HY4		HY3 vs HY4	
	p	*d*	*p*	*d*	*p*	*d*	*p*	*d*	*p*	*d*	*p*	*d*
**L Amygdala**	0.766	0.014	**0.002**	**-0.213**	**<0.001**	**-0.454**	**<0.001**	**-0.239**	**<0.001**	**-0.359**	0.787	-0.033
R Amygdala	0.365	-0.042	0.009	-0.173	**0.001**	**-0.327**	0.017	-0.144	0.010	-0.252	0.808	-0.028
L Caudate nucleus	0.065	-0.096	0.114	-0.115	0.037	-0.234	0.174	-0.086	0.125	-0.160	0.847	0.024
R Caudate nucleus	0.151	-0.074	0.217	-0.087	0.226	-0.133	0.093	-0.104	0.467	-0.076	0.360	0.115
L Globus pallidus	0.774	0.014	0.876	0.010	0.114	-0.163	0.721	-0.022	0.092	-0.168	0.193	-0.133
R Globus pallidus	0.307	0.051	0.644	0.031	0.304	-0.109	0.157	-0.085	0.033	-0.212	0.248	-0.115
L Hippocampus	0.215	-0.063	0.011	-0.186	**0.003**	**-0.335**	**0.001**	**-0.201**	**<0.001**	**-0.393**	0.039	-0.253
R Hippocampus	0.126	-0.075	**0.002**	**-0.227**	**<0.001**	**-0.439**	**0.002**	**-0.191**	**<0.001**	**-0.425**	0.072	-0.214
L Lateral ventricle	0.710	-0.021	0.022	0.175	0.022	0.269	0.013	0.168	0.006	0.306	0.077	0.221
R Lateral ventricle	0.661	-0.025	0.074	0.136	0.007	0.321	0.067	0.124	**0.001**	**0.360**	0.019	0.290
L Nucleus accumbens	0.428	0.039	0.130	-0.101	0.150	-0.150	0.060	-0.109	0.009	-0.254	0.358	-0.098
R Nucleus accumbens	0.651	-0.023	0.282	-0.075	0.005	-0.306	0.079	-0.106	**0.001**	**-0.335**	0.127	-0.178
L Putamen	0.748	0.016	0.098	-0.117	0.015	-0.268	0.005	-0.174	**0.003**	**-0.306**	0.146	-0.174

R Putamen	0.983	0.001	0.368	-0.063	0.023	-0.250	0.075	-0.106	**0.001**	**-0.340**	0.018	-0.273
L Thalamus	0.213	-0.055	0.061	-0.123	0.044	-0.209	0.200	-0.071	0.024	-0.205	0.120	-0.166
R Thalamus	0.598	-0.024	0.023	-0.146	0.126	-0.154	0.031	-0.126	0.021	-0.217	0.348	-0.100

Supplementary table 12: MoCA sample characteristics

Cohort	N	Mean age, years (SD)	% Female	DURILL, years (SD)	MoCA (SD)
Amsterdam II	61	62.5 (7.08)	39	5.3 (3.54)	26.3 (2.15)
BE I	2	73 (4.24)	50	15.5 (4.95)	23 (5.66)
Charlottesville I	86	63.7 (8.45)	23	9.9 (5.42)	24.9 (3.44)
Charlottesville II	25	60.6 (9.36)	12	9.5 (3.98)	24.3 (3.45)
Charlottesville III	18	69.3 (6.24)	33	8.4 (3.41)	23.4 (4.6)
NW-England I	32	69.9 (8.58)	19	6.8 (4.42)	24.8 (4.25)
NW-England II	12	64.8 (6.13)	33	10.3 (5.86)	26.3 (2.93)
Oxford DISCOVERY	113	63.7 (9.87)	36	2.3 (1.57)	26.6 (2.72)
PDNZ	207	69.4 (7.79)	27	5.8 (5.58)	23.6 (4.18)
PPMI 1	15	62.9 (8.19)	47	0.6 (0.52)	27.1 (1.91)
PPMI 2	15	62.4 (8.31)	67	0.7 (0.77)	26.3 (2.09)
PPMI 3	17	60.2 (10.46)	29	0.8 (0.83)	27.6 (1.9)
PPMI 4	10	66.4 (10.72)	60	0.9 (0.76)	28.4 (1.17)
PPMI 5	15	64.3 (12.07)	33	0.8 (0.65)	26.9 (2.17)
PPMI 6	2	61.3 (7.78)	0	1.3 (0.14)	25 (1.41)
PPMI 7	11	59.1 (12.53)	18	0.5 (0.34)	29 (1.1)
PPMI 8	13	66.4 (7.19)	46	0.5 (0.27)	27.9 (1.5)
PPMI 9	18	63.2 (7.85)	39	0.6 (0.39)	28.2 (1.76)
PPMI 10	21	63.8 (9.82)	33	0.3 (0.23)	26.4 (2.94)
PPMI 11	6	60.6 (11.09)	67	0.2 (0.04)	27.5 (1.52)
PPMI 12	21	59.5 (10.55)	33	0.6 (0.57)	28.4 (1.66)
PPMI 13	6	59.6 (12.46)	17	0.2 (0.08)	27 (2.37)
PPMI 14	62	60.7 (10.16)	29	0.4 (0.38)	25.8 (2.68)
PPMI 15	15	58.5 (7.83)	20	0.5 (0.44)	27.8 (1.47)
PPMI 16	15	62.0 (7.51)	27	0.7 (0.68)	27.7 (1.53)
PPMI 17	19	66.4 (9.08)	47	0.7 (0.47)	26.6 (2.29)
PPMI 18	22	58.3 (9.23)	23	0.4 (0.52)	28.9 (1.19)
PPMI 19	19	59.3 (9.3)	26	0.7 (0.69)	27.4 (1.54)
PPMI 20	3	63.6 (7.71)	0	0.3 (0.06)	25.3 (2.52)
PPMI 21	22	62.0 (9.14)	41	0.8 (0.80)	26.1 (2.09)
Stanford	43	69.0 (8.17)	49	5.6 (3.47)	23.9 (5.21)
UDALL/U19	111	66.3 (7.88)	32	7.3 (5.50)	25.5 (3.31)
**Total**	**1057**	**64.8 (9.28)**	**32**	**4.4 (5.05)**	**25.6 (3.55)**

Demographics for the MoCA regression analysis. SD – standard deviation, DURILL – duration of illness, MoCA – Montreal Cognitive Assessment.

Supplementary table 13: DURILL sample characteristics

Cohort	N	Mean age, years (SD)	% Female	DURILL, years (SD)	MoCA (SD)
Amsterdam I	104	63.1 (10.87)	38	2.1 (3.39)	NA
Amsterdam II	61	62.5 (7.08)	39	5.3 (3.54)	26.3 (2.15)

BE I	52	62.9 (10.38)	52	12.4 (4.29)	23 (5.66)
BE II	3	59.7 (6.66)	67	11.3 (7.57)	NA
CGU	291	60.6 (9.37)	45	8.7 (6.33)	NA
Charlottesville I	116	63.7 (8.52)	28	9.7 (5.09)	24.9 (3.44)
Charlottesville II	37	62.4 (9.59)	14	8.7 (3.64)	24.3 (3.45)
Charlottesville III	24	70.8 (6.77)	29	7.7 (3.23)	23.4 (4.6)
Donders	57	61 (10.18)	46	4.4 (3.79)	NA
Liege I	30	65.9 (6.61)	37	7.2 (5.32)	NA
Liege II	45	66.9 (8.24)	44	6 (3.93)	NA
Milan	43	57.8 (7.8)	33	11.4 (3.38)	NA
NW-England I	32	69.9 (8.58)	19	6.8 (4.42)	24.8 (4.25)
NW-England II	14	65 (5.67)	29	9.2 (6.02)	26.3 (2.93)
Oxford DISCOVERY	114	63.9 (10.09)	36	2.3 (1.58)	26.6 (2.72)
PDNZ	208	69.4 (7.79)	26	5.7 (5.57)	23.6 (4.19)
PPMI 1	15	62.9 (8.19)	47	0.6 (0.52)	27.1 (1.91)
PPMI 2	15	62.4 (8.31)	67	0.7 (0.77)	26.3 (2.09)
PPMI 3	17	60.2 (10.46)	29	0.8 (0.83)	27.6 (1.9)
PPMI 4	10	66.4 (10.72)	60	0.9 (0.76)	28.4 (1.17)
PPMI 5	15	64.3 (12.07)	33	0.8 (0.65)	26.9 (2.17)
PPMI 6	2	61.3 (7.78)	0	1.3 (0.14)	25 (1.41)
PPMI 7	11	59.1 (12.53)	18	0.5 (0.34)	29 (1.1)
PPMI 8	13	66.4 (7.19)	46	0.5 (0.27)	27.9 (1.5)
PPMI 9	18	63.2 (7.85)	39	0.6 (0.39)	28.2 (1.76)
PPMI 10	21	63.8 (9.82)	33	0.3 (0.23)	26.4 (2.94)
PPMI 11	6	60.6 (11.09)	67	0.2 (0.04)	27.5 (1.52)
PPMI 12	21	59.5 (10.55)	33	0.6 (0.57)	28.4 (1.66)
PPMI 13	6	59.6 (12.46)	17	0.2 (0.08)	27 (2.37)
PPMI 14	62	60.7 (10.16)	29	0.4 (0.38)	25.8 (2.68)
PPMI 15	15	58.5 (7.83)	20	0.5 (0.44)	27.8 (1.47)
PPMI 16	15	62 (7.51)	27	0.7 (0.68)	27.7 (1.53)
PPMI 17	19	66.4 (9.08)	47	0.7 (0.47)	26.6 (2.29)
PPMI 18	22	58.3 (9.23)	23	0.4 (0.52)	28.9 (1.19)
PPMI 19	19	59.3 (9.3)	26	0.7 (0.69)	27.4 (1.54)
PPMI 20	3	63.6 (7.71)	0	0.3 (0.06)	25.3 (2.52)
PPMI 21	22	62 (9.14)	41	0.8 (0.8)	26.1 (2.09)
PROMOVE/ASPS I	100	63.2 (10.15)	29	4.7 (4.77)	NA
PROMOVE/ASPS II	23	64 (9.9)	22	4 (5.69)	NA
Rome SLF	225	62.7 (10.14)	36	4.9 (4.17)	NA
Stanford	44	68.6 (8.49)	50	5.6 (3.44)	23.9 (5.21)
Tao Wu	19	65 (4.45)	47	5.3 (4)	NA
UDALL/U19	112	66.4 (7.87)	32	7.3 (5.48)	25.5 (3.31)
UNICAMP	110	59.9 (10.2)	34	7.3 (6.41)	NA
**Total**	**2211**	**63.4 (9.69)**	**35**	**5.5 (5.47)**	**25.6 (3.55)**

Demographics for the duration of illness regression analysis. SD – standard deviation, DURILL – duration of illness, MoCA – Montreal Cognitive Assessment.

Supplementary table 14a: Results of regression analysis of duration of illness and cortical thickness

ROI Thickness	*p*	*r*	b	SE	Lower CI	Upper CI	NPD
L Banks STS	0.019	-0.042	-0.002	0.001	-0.004	<0.001	1933
R Banks STS	0.126	-0.026	-0.001	0.001	-0.003	<0.001	2071

L Caudal anterior cingulate	0.386	0.016	0.001	0.001	-0.001	0.004	2156
R Caudal anterior cingulate	0.885	0.003	<0.001	0.001	-0.002	0.003	2154
L Caudal middle frontal	0.053	-0.035	-0.001	0.001	-0.003	<0.001	2163
R Caudal middle frontal	0.117	-0.029	-0.001	0.001	-0.003	<0.001	2158
L Cuneus	0.345	-0.017	-0.001	0.001	-0.002	0.001	2016
R Cuneus	0.990	<0.001	<0.001	0.001	-0.001	0.001	1985
L Entorhinal	0.081	-0.033	-0.003	0.002	-0.006	<0.001	2123
R Entorhinal	0.006	-0.044	-0.005	0.002	-0.008	-0.001	2068
L Frontal pole	0.727	-0.007	<0.001	0.001	-0.003	0.002	2168
R Frontal pole	0.739	0.006	<0.001	0.001	-0.002	0.003	2164
L Fusiform	0.001	-0.056	-0.003	0.001	-0.004	-0.001	2135
R Fusiform	0.006	-0.041	-0.002	0.001	-0.004	-0.001	2147
**L Inferior parietal**	**<0.001**	**-0.063**	**-0.003**	**0.001**	**-0.004**	**-0.001**	**2079**
R Inferior parietal	0.001	-0.057	-0.002	0.001	-0.004	-0.001	2091
L Inferior temporal	0.023	-0.040	-0.002	0.001	-0.004	<0.001	2074
R Inferior temporal	0.043	-0.034	-0.002	0.001	-0.003	<0.001	2101
L Insula	0.085	-0.034	-0.002	0.001	-0.003	<0.001	2163
R Insula	0.024	-0.039	-0.002	0.001	-0.004	<0.001	2153
L Isthmus cingulate	0.071	-0.035	-0.002	0.001	-0.004	<0.001	2162
R Isthmus cingulate	0.757	0.006	<0.001	0.001	-0.002	0.002	2158
L Lateral occipital	0.177	-0.025	-0.001	0.001	-0.002	<0.001	2088
R Lateral occipital	0.003	-0.054	-0.002	0.001	-0.004	-0.001	2105
L Lateral orbitofrontal	0.287	-0.018	-0.001	0.001	-0.002	0.001	2166
R Lateral orbitofrontal	0.241	-0.018	-0.001	0.001	-0.002	0.001	2163
L Lingual	0.447	-0.014	<0.001	0.001	-0.002	0.001	2113
R Lingual	0.069	-0.030	-0.001	0.001	-0.002	<0.001	2076
L Medial orbitofrontal	0.753	-0.006	<0.001	0.001	-0.002	0.001	2146
R Medial orbitofrontal	0.293	-0.017	-0.001	0.001	-0.002	0.001	2140
L Middle temporal	0.004	-0.052	-0.002	0.001	-0.004	-0.001	1970
R Middle temporal	0.039	-0.035	-0.002	0.001	-0.003	<0.001	2088
L Paracentral	0.543	-0.011	-0.001	0.001	-0.002	0.001	2153
R Paracentral	0.262	-0.020	-0.001	0.001	-0.003	0.001	2141
L Parahippocampal	0.101	-0.031	-0.002	0.001	-0.005	<0.001	2167
R Parahippocampal	0.989	<0.001	<0.001	0.001	-0.002	0.002	2156
L Pars opercularis	0.008	-0.047	-0.002	0.001	-0.003	-0.001	2159
R Pars opercularis	0.094	-0.029	-0.001	0.001	-0.003	<0.001	2155
L Pars orbitalis	0.671	-0.008	<0.001	0.001	-0.002	0.001	2157
R Pars orbitalis	0.687	-0.007	<0.001	0.001	-0.002	0.001	2154
L Pars triangularis	0.004	-0.050	-0.002	0.001	-0.004	-0.001	2153
R Pars triangularis	0.539	-0.011	<0.001	0.001	-0.002	0.001	2142
L Pericalcarine	0.296	-0.018	-0.001	0.001	-0.002	0.001	2020
R Pericalcarine	0.868	0.003	<0.001	0.001	-0.001	0.002	1956
L Postcentral	0.020	-0.041	-0.001	0.001	-0.003	<0.001	2041
R Postcentral	0.017	-0.039	-0.002	0.001	-0.003	<0.001	2037
L Posterior cingulate	0.369	-0.016	-0.001	0.001	-0.002	0.001	2168
R Posterior cingulate	0.004	-0.049	-0.002	0.001	-0.004	-0.001	2167
L Precentral	0.042	-0.036	-0.002	0.001	-0.003	<0.001	2073
R Precentral	0.072	-0.033	-0.002	0.001	-0.003	<0.001	2084
**L Precuneus**	**<0.001**	**-0.060**	**-0.002**	**0.001**	**-0.004**	**-0.001**	**2162**
**R Precuneus**	**<0.001**	**-0.060**	**-0.002**	**0.001**	**-0.004**	**-0.001**	**2164**
L Rostral anterior cingulate	0.290	-0.020	-0.001	0.001	-0.004	0.001	2145

R Rostral anterior cingulate	0.719	0.006	<0.001	0.001	-0.002	0.003	2142
L Rostral middle frontal	0.075	-0.029	-0.001	0.001	-0.002	<0.001	2149
R Rostral middle frontal	0.147	-0.022	-0.001	0.001	-0.002	<0.001	2157
L Superior frontal	0.104	-0.029	-0.001	0.001	-0.003	<0.001	2119
R Superior frontal	0.017	-0.040	-0.002	0.001	-0.003	<0.001	2122
L Superior parietal	0.002	-0.053	-0.002	0.001	-0.003	-0.001	2098
**R Superior parietal**	**<0.001**	**-0.058**	**-0.002**	**0.001**	**-0.004**	**-0.001**	**2117**
L Superior temporal	0.018	-0.043	-0.002	0.001	-0.004	<0.001	1892
R Superior temporal	0.225	-0.019	-0.001	0.001	-0.003	0.001	2010
L Supramarginal	0.055	-0.034	-0.001	0.001	-0.003	<0.001	2051
R Supramarginal	0.017	-0.043	-0.002	0.001	-0.003	<0.001	2076
L Temporal pole	0.009	-0.052	-0.004	0.002	-0.007	-0.001	2077
R Temporal pole	0.038	-0.038	-0.003	0.002	-0.007	<0.001	2083
L Transverse temporal	0.002	-0.058	-0.003	0.001	-0.005	-0.001	2178
R Transverse temporal	0.010	-0.038	-0.003	0.001	-0.005	-0.001	2175

Results of regression analysis of duration of illness and cortical thickness in PD sample. L – Left, R – Right, ROI – region of interest. Banks STS – Banks of the superior temporal sulcus. **Boldface** denotes significant results after Bonferroni correction.

Supplementary table 14b: Results of regression analysis of duration of illness and surface area

ROI Surface Area	*p*	*r*	b	SE	Lower CI	Upper CI	NPD
L Banks STS	0.045	-0.044	-1.400	0.697	-2.767	-0.032	1931
R Banks STS	0.066	-0.037	-1.110	0.604	-2.295	0.075	2072
L Caudal anterior cingulate	0.270	-0.023	-0.656	0.595	-1.822	0.511	2155
R Caudal anterior cingulate	0.005	-0.058	-1.940	0.682	-3.278	-0.602	2150
L Caudal middle frontal	0.217	-0.025	-1.825	1.478	-4.723	1.073	2160
R Caudal middle frontal	0.719	-0.007	-0.528	1.469	-3.409	2.353	2159
L Cuneus	0.167	-0.030	-1.271	0.919	-3.073	0.531	2016
R Cuneus	0.264	-0.024	-1.027	0.918	-2.827	0.774	1981
L Entorhinal	0.710	-0.008	-0.144	0.388	-0.905	0.616	2123
R Entorhinal	0.123	0.032	0.553	0.358	-0.150	1.256	2071
L Frontal pole	0.707	-0.007	-0.054	0.145	-0.338	0.230	2166
R Frontal pole	0.647	0.009	0.087	0.189	-0.284	0.458	2166
L Fusiform	0.218	-0.025	-1.979	1.605	-5.127	1.169	2135
R Fusiform	0.007	-0.055	-4.367	1.605	-7.515	-1.218	2147
L Inferior parietal	0.340	-0.019	-2.367	2.482	-7.234	2.500	2080
R Inferior parietal	0.186	-0.028	-3.681	2.783	-9.139	1.777	2095
L Inferior temporal	0.006	-0.057	-5.395	1.945	-9.209	-1.581	2077
R Inferior temporal	0.310	-0.021	-1.755	1.727	-5.142	1.632	2103
L Insula	0.367	0.017	0.879	0.976	-1.034	2.793	2162
R Insula	0.638	-0.009	-0.536	1.140	-2.772	1.699	2153
L Isthmus cingulate	0.898	0.003	0.092	0.716	-1.312	1.495	2162
R Isthmus cingulate	0.893	0.003	0.088	0.655	-1.195	1.372	2157
L Lateral occipital	0.116	-0.032	-3.855	2.452	-8.663	0.953	2090
R Lateral occipital	0.478	0.015	1.805	2.543	-3.184	6.793	2106
L Lateral orbitofrontal	0.008	-0.055	-3.004	1.131	-5.222	-0.786	2166
R Lateral orbitofrontal	0.061	-0.038	-2.330	1.245	-4.772	0.112	2161
L Lingual	0.002	-0.063	-5.567	1.821	-9.139	-1.996	2110

R Lingual	0.026	-0.046	-3.969	1.786	-7.472	-0.466	2077
**L Medial orbitofrontal**	**<0.001**	**-0.078**	**-3.600**	**0.936**	**-5.435**	**-1.765**	**2145**
R Medial orbitofrontal	0.050	-0.040	-1.617	0.823	-3.230	-0.003	2140
L Middle temporal	0.050	-0.041	-3.238	1.647	-6.469	-0.007	1970
R Middle temporal	0.004	-0.058	-4.697	1.634	-7.903	-1.492	2088
L Paracentral	0.392	-0.017	-0.666	0.778	-2.192	0.860	2150
R Paracentral	0.252	-0.023	-1.073	0.937	-2.911	0.765	2141
L Parahippocampal	0.058	-0.038	-0.855	0.451	-1.739	0.028	2166
R Parahippocampal	0.020	-0.047	-0.981	0.423	-1.811	-0.152	2153
L Pars opercularis	0.222	0.025	1.279	1.047	-0.774	3.331	2161
R Pars opercularis	0.818	0.005	0.205	0.892	-1.544	1.955	2156
L Pars orbitalis	0.187	-0.027	-0.451	0.342	-1.121	0.219	2155
R Pars orbitalis	0.119	-0.032	-0.666	0.427	-1.503	0.172	2157
L Pars triangularis	0.849	0.004	0.151	0.792	-1.402	1.704	2153
R Pars triangularis	0.661	-0.009	-0.449	1.024	-2.457	1.559	2143
L Pericalcarine	0.029	-0.047	-2.466	1.131	-4.683	-0.248	2018
R Pericalcarine	0.004	-0.063	-3.362	1.159	-5.635	-1.089	1955
L Postcentral	0.931	0.002	0.161	1.852	-3.471	3.792	2042
R Postcentral	0.654	0.010	0.845	1.884	-2.850	4.540	2035
L Posterior cingulate	0.124	-0.032	-1.383	0.900	-3.147	0.381	2168
R Posterior cingulate	0.022	-0.046	-1.732	0.754	-3.212	-0.253	2165
L Precentral	0.862	-0.004	-0.360	2.065	-4.409	3.689	2072
R Precentral	0.840	-0.004	-0.434	2.149	-4.648	3.781	2085
L Precuneus	0.509	-0.013	-1.164	1.764	-4.624	2.296	2165
R Precuneus	0.470	-0.015	-1.385	1.917	-5.145	2.374	2164
L Rostral anterior cingulate	0.222	-0.025	-0.829	0.679	-2.160	0.502	2140
R Rostral anterior cingulate	0.002	-0.060	-1.778	0.584	-2.924	-0.632	2144
L Rostral middle frontal	0.003	-0.060	-8.506	2.827	-14.050	-2.961	2149
R Rostral middle frontal	0.015	-0.048	-7.132	2.921	-12.860	-1.405	2160
L Superior frontal	0.098	-0.033	-4.940	2.987	-10.799	0.919	2121
R Superior frontal	0.082	-0.035	-5.251	3.020	-11.174	0.671	2124
L Superior parietal	0.411	-0.017	-2.086	2.536	-7.060	2.887	2098
R Superior parietal	0.335	-0.020	-2.426	2.517	-7.363	2.510	2115
L Superior temporal	0.642	-0.010	-0.829	1.785	-4.329	2.671	1892
R Superior temporal	0.002	-0.063	-4.726	1.526	-7.719	-1.732	2011
L Supramarginal	0.138	-0.031	-3.486	2.350	-8.096	1.123	2050
R Supramarginal	0.550	-0.013	-1.254	2.095	-5.362	2.854	2081
L Temporal pole	0.548	-0.013	-0.166	0.276	-0.707	0.375	2079
R Temporal pole	0.130	0.031	0.430	0.284	-0.127	0.987	2083
L Transverse temporal	0.587	0.011	0.179	0.329	-0.466	0.823	2177
R Transverse temporal	0.896	-0.003	-0.031	0.234	-0.489	0.428	2176

Results of regression analysis of duration of illness and cortical surface area in PD sample. L – Left, R – Right, ROI – region of interest. Banks STS – Banks of the superior temporal sulcus. **Boldface** denotes significant results after Bonferroni correction.

Supplementary table 14c: Results of regression analysis of duration of illness and subcortical volume

ROI Subcortical	*p*	*r*	b	SE	Lower CI	Upper CI	NPD
**L Amygdala**	**<0.001**	**-0.105**	**-5.48**	**0.96**	**-7.37**	**-3.59**	**2153**
**R Amygdala**	**<0.001**	**-0.102**	**-5.88**	**1.04**	**-7.92**	**-3.83**	**2147**

**L Caudate nucleus**	**<0.001**	**-0.100**	**-10.85**	**2.11**	**-14.99**	**-6.71**	**2172**
**R Caudate nucleus**	**<0.001**	**-0.084**	**-9.17**	**2.11**	**-13.31**	**-5.02**	**2176**
L Globus pallidus	0.613	0.009	0.61	1.20	-1.74	2.95	2059
R Globus pallidus	0.908	0.002	0.12	1.06	-1.96	2.20	2120
**L Hippocampus**	**0.002**	**-0.062**	**-6.40**	**2.02**	**-10.36**	**-2.45**	**2128**
**R Hippocampus**	**<0.001**	**-0.065**	**-7.20**	**2.10**	**-11.32**	**-3.09**	**2159**
L Lateral ventricle	0.656	0.009	13.90	31.20	-47.29	75.09	2197
R Lateral ventricle	0.378	0.018	25.67	29.10	-31.40	82.73	2198
**L Nucleus accumbens**	**<0.001**	**-0.064**	**-1.79**	**0.50**	**-2.77**	**-0.81**	**2164**
R Nucleus accumbens	0.005	-0.053	-1.34	0.48	-2.28	-0.40	2138
L Putamen	<0.001	-0.070	-10.66	2.90	-16.35	-4.97	2047
R Putamen	0.010	-0.049	-7.11	2.75	-12.50	-1.73	2103
**L Thalamus**	**0.003**	**-0.051**	**-10.25**	**3.43**	**-16.98**	**-3.53**	**2072**
**R Thalamus**	**<0.001**	**-0.058**	**-9.88**	**2.90**	**-15.57**	**-4.19**	**2128**

Results of regression analysis of duration of illness and subcortical volume in PD sample. L – Left, R – Right, ROI – region of interest. **Boldface** denotes significant results after Bonferroni correction.

Supplementary table 14d: Results of regression analysis of duration of illness and cortical thickness – uncorrected for age

ROI Surface Area	*p*	*r*	b	SE	Lower CI	Upper CI	NPD
L Banks STS	0.001	-0.058	-0.003	0.001	-0.005	-0.001	1933
R Banks STS	0.014	-0.043	-0.002	0.001	-0.004	<0.001	2071
L Caudal anterior cingulate	0.226	0.023	0.002	0.001	-0.001	0.004	2156
R Caudal anterior cingulate	0.748	0.006	<0.001	0.001	-0.002	0.003	2154
L Caudal middle frontal	0.007	-0.050	-0.002	0.001	-0.004	-0.001	2163
R Caudal middle frontal	0.028	-0.041	-0.002	0.001	-0.003	<0.001	2158
L Cuneus	0.143	-0.027	-0.001	0.001	-0.002	<0.001	2016
R Cuneus	0.553	-0.010	<0.001	0.001	-0.002	0.001	1985
L Entorhinal	0.014	-0.047	-0.004	0.002	-0.007	-0.001	2123
R Entorhinal	0.001	-0.055	-0.006	0.002	-0.009	-0.002	2068
L Frontal pole	0.413	-0.016	-0.001	0.001	-0.004	0.001	2168
R Frontal pole	0.923	-0.002	<0.001	0.001	-0.003	0.002	2164
**L Fusiform**	**<0.001**	**-0.073**	**-0.003**	**0.001**	**-0.005**	**-0.002**	**2135**
**R Fusiform**	**<0.001**	**-0.055**	**-0.003**	**0.001**	**-0.005**	**-0.002**	**2147**
**L Inferior parietal**	**<0.001**	**-0.082**	**-0.003**	**0.001**	**-0.005**	**-0.002**	**2079**
**R Inferior parietal**	**<0.001**	**-0.073**	**-0.003**	**0.001**	**-0.005**	**-0.002**	**2091**
L Inferior temporal	0.003	-0.054	-0.003	0.001	-0.004	-0.001	2074
R Inferior temporal	0.005	-0.047	-0.002	0.001	-0.004	-0.001	2101
L Insula	0.020	-0.046	-0.002	0.001	-0.004	<0.001	2163
R Insula	0.005	-0.049	-0.003	0.001	-0.005	-0.001	2153
L Isthmus cingulate	0.008	-0.052	-0.003	0.001	-0.004	-0.001	2162
R Isthmus cingulate	0.530	-0.012	-0.001	0.001	-0.003	0.001	2158
L Lateral occipital	0.029	-0.041	-0.002	0.001	-0.003	<0.001	2088
**R Lateral occipital**	**<0.001**	**-0.069**	**-0.003**	**0.001**	**-0.004**	**-0.001**	**2105**
L Lateral orbitofrontal	0.119	-0.026	-0.001	0.001	-0.003	<0.001	2166
R Lateral orbitofrontal	0.079	-0.027	-0.001	0.001	-0.003	<0.001	2163
L Lingual	0.054	-0.035	-0.001	0.001	-0.003	<0.001	2113
R Lingual	0.005	-0.046	-0.002	0.001	-0.003	-0.001	2076

L Medial orbitofrontal	0.412	-0.015	-0.001	0.001	-0.002	0.001	2146
R Medial orbitofrontal	0.175	-0.022	-0.001	0.001	-0.003	<0.001	2140
**L Middle temporal**	**<0.001**	**-0.066**	**-0.003**	**0.001**	**-0.005**	**-0.001**	**1970**
R Middle temporal	0.005	-0.048	-0.002	0.001	-0.004	-0.001	2088
L Paracentral	0.154	-0.025	-0.001	0.001	-0.003	<0.001	2153
R Paracentral	0.047	-0.036	-0.002	0.001	-0.004	<0.001	2141
L Parahippocampal	0.028	-0.042	-0.003	0.001	-0.006	<0.001	2167
R Parahippocampal	0.482	-0.013	-0.001	0.001	-0.003	0.002	2156
**L Pars opercularis**	**0.001**	**-0.061**	**-0.003**	**0.001**	**-0.004**	**-0.001**	**2159**
R Pars opercularis	0.021	-0.040	-0.002	0.001	-0.003	<0.001	2155
L Pars orbitalis	0.269	-0.021	-0.001	0.001	-0.003	0.001	2157
R Pars orbitalis	0.304	-0.019	-0.001	0.001	-0.003	0.001	2154
**L Pars triangularis**	**<0.001**	**-0.066**	**-0.003**	**0.001**	**-0.004**	**-0.001**	**2153**
R Pars triangularis	0.169	-0.024	-0.001	0.001	-0.002	<0.001	2142
L Pericalcarine	0.093	-0.030	-0.001	0.001	-0.003	<0.001	2020
R Pericalcarine	0.701	-0.006	<0.001	0.001	-0.002	0.001	1956
L Postcentral	0.001	-0.058	-0.002	0.001	-0.003	-0.001	2041
R Postcentral	0.001	-0.055	-0.002	0.001	-0.004	-0.001	2037
L Posterior cingulate	0.146	-0.027	-0.001	0.001	-0.003	<0.001	2168
**R Posterior cingulate**	**<0.001**	**-0.059**	**-0.003**	**0.001**	**-0.004**	**-0.001**	**2167**
L Precentral	0.003	-0.054	-0.003	0.001	-0.004	-0.001	2073
R Precentral	0.005	-0.051	-0.002	0.001	-0.004	-0.001	2084
**L Precuneus**	**<0.001**	**-0.077**	**-0.003**	**0.001**	**-0.005**	**-0.002**	**2162**
**R Precuneus**	**<0.001**	**-0.079**	**-0.003**	**0.001**	**-0.005**	**-0.002**	**2164**
L Rostral anterior cingulate	0.313	-0.019	-0.001	0.001	-0.003	0.001	2145
R Rostral anterior cingulate	0.549	0.011	0.001	0.001	-0.002	0.003	2142
L Rostral middle frontal	0.012	-0.041	-0.002	0.001	-0.003	<0.001	2149
R Rostral middle frontal	0.045	-0.031	-0.001	0.001	-0.003	<0.001	2157
L Superior frontal	0.008	-0.048	-0.002	0.001	-0.003	-0.001	2119
R Superior frontal	0.001	-0.055	-0.002	0.001	-0.004	-0.001	2122
**L Superior parietal**	**<0.001**	**-0.067**	**-0.003**	**0.001**	**-0.004**	**-0.001**	**2098**
**R Superior parietal**	**<0.001**	**-0.071**	**-0.003**	**0.001**	**-0.004**	**-0.002**	**2117**
**L Superior temporal**	**<0.001**	**-0.068**	**-0.003**	**0.001**	**-0.005**	**-0.002**	**1892**
R Superior temporal	0.011	-0.041	-0.002	0.001	-0.004	<0.001	2010
L Supramarginal	0.002	-0.055	-0.002	0.001	-0.004	-0.001	2051
**R Supramarginal**	**0.001**	**-0.061**	**-0.003**	**0.001**	**-0.004**	**-0.001**	**2076**
L Temporal pole	0.002	-0.061	-0.005	0.002	-0.008	-0.002	2077
R Temporal pole	0.008	-0.048	-0.004	0.002	-0.008	-0.001	2083
**L Transverse temporal**	**<0.001**	**-0.075**	**-0.004**	**0.001**	**-0.006**	**-0.002**	**2178**
**R Transverse temporal**	**<0.001**	**-0.053**	**-0.004**	**0.001**	**-0.007**	**-0.002**	**2175**

Results of regression analysis of duration of illness and cortical surface area in PD sample. L – Left, R – Right, ROI – region of interest. Banks STS – Banks of the superior temporal sulcus. **Boldface** denotes significant results after Bonferroni correction.

Supplementary table 14e: Results of regression analysis of duration of illness and cortical surface area – uncorrected for age

ROI Surface Area	*p*	*r*	b	SE	Lower CI	Upper CI	NPD
L Banks STS	0.008	-0.058	-1.889	0.706	-3.275	-0.503	1931
R Banks STS	0.009	-0.053	-1.602	0.613	-2.803	-0.401	2072

L Caudal anterior cingulate	0.108	-0.033	-0.961	0.597	-2.133	0.210	2155
R Caudal anterior cingulate	0.001	-0.066	-2.239	0.684	-3.581	-0.897	2150
L Caudal middle frontal	0.071	-0.037	-2.691	1.488	-5.609	0.227	2160
R Caudal middle frontal	0.379	-0.018	-1.301	1.477	-4.196	1.595	2159
L Cuneus	0.030	-0.047	-2.025	0.930	-3.850	-0.201	2016
R Cuneus	0.066	-0.040	-1.712	0.931	-3.538	0.113	1981
L Entorhinal	0.495	-0.014	-0.264	0.387	-1.023	0.494	2123
R Entorhinal	0.154	0.030	0.508	0.357	-0.192	1.208	2071
L Frontal pole	0.485	-0.013	-0.101	0.145	-0.385	0.183	2166
R Frontal pole	0.908	0.002	0.022	0.189	-0.349	0.393	2166
L Fusiform	0.022	-0.047	-3.809	1.657	-7.059	-0.559	2135
**R Fusiform**	**<0.001**	**-0.075**	**-6.086**	**1.653**	**-9.328**	**-2.844**	**2147**
L Inferior parietal	0.074	-0.036	-4.528	2.531	-9.491	0.435	2080
R Inferior parietal	0.024	-0.047	-6.488	2.865	-12.107	-0.869	2095
**L Inferior temporal**	**<0.001**	**-0.074**	**-7.115**	**1.988**	**-11.013**	**-3.217**	**2077**
R Inferior temporal	0.046	-0.041	-3.532	1.769	-7.002	-0.063	2103
L Insula	0.590	0.010	0.527	0.976	-1.388	2.441	2162
R Insula	0.377	-0.017	-1.007	1.141	-3.245	1.230	2153
L Isthmus cingulate	0.776	-0.006	-0.204	0.717	-1.610	1.202	2162
R Isthmus cingulate	0.788	-0.005	-0.176	0.656	-1.462	1.110	2157
L Lateral occipital	0.013	-0.051	-6.264	2.512	-11.190	-1.338	2090
R Lateral occipital	0.915	-0.002	-0.277	2.587	-5.351	4.796	2106
**L Lateral orbitofrontal**	**<0.001**	**-0.073**	**-4.117**	**1.161**	**-6.394**	**-1.841**	**2166**
R Lateral orbitofrontal	0.009	-0.053	-3.300	1.266	-5.782	-0.819	2161
**L Lingual**	**<0.001**	**-0.084**	**-7.558**	**1.862**	**-11.210**	**-3.906**	**2110**
R Lingual	0.002	-0.064	-5.619	1.824	-9.196	-2.042	2077
**L Medial orbitofrontal**	**<0.001**	**-0.088**	**-4.095**	**0.941**	**-5.940**	**-2.251**	**2145**
R Medial orbitofrontal	0.010	-0.052	-2.135	0.829	-3.760	-0.510	2140
L Middle temporal	0.006	-0.058	-4.636	1.694	-7.958	-1.315	1970
**R Middle temporal**	**<0.001**	**-0.076**	**-6.214**	**1.674**	**-9.497**	**-2.931**	**2088**
L Paracentral	0.204	-0.026	-0.992	0.780	-2.521	0.538	2150
R Paracentral	0.123	-0.031	-1.447	0.939	-3.288	0.394	2141
L Parahippocampal	0.006	-0.055	-1.267	0.462	-2.172	-0.361	2166
R Parahippocampal	0.002	-0.062	-1.329	0.431	-2.175	-0.484	2153
L Pars opercularis	0.546	0.012	0.637	1.055	-1.431	2.706	2161
R Pars opercularis	0.587	-0.011	-0.492	0.905	-2.267	1.283	2156
L Pars orbitalis	0.040	-0.041	-0.711	0.347	-1.391	-0.032	2155
R Pars orbitalis	0.019	-0.048	-1.021	0.434	-1.873	-0.170	2157
L Pars triangularis	0.613	-0.011	-0.403	0.798	-1.969	1.162	2153
R Pars triangularis	0.240	-0.024	-1.215	1.034	-3.242	0.812	2143
L Pericalcarine	0.007	-0.058	-3.033	1.133	-5.254	-0.811	2018
**R Pericalcarine**	**0.001**	**-0.073**	**-3.953**	**1.164**	**-6.236**	**-1.671**	**1955**
L Postcentral	0.580	-0.012	-1.032	1.867	-4.693	2.629	2042
R Postcentral	0.951	-0.001	-0.116	1.892	-3.827	3.594	2035
L Posterior cingulate	0.030	-0.045	-1.978	0.909	-3.761	-0.195	2168
R Posterior cingulate	0.002	-0.063	-2.442	0.770	-3.953	-0.931	2165
L Precentral	0.509	-0.013	-1.370	2.072	-5.432	2.693	2072
R Precentral	0.492	-0.014	-1.479	2.153	-5.702	2.744	2085
L Precuneus	0.102	-0.033	-2.970	1.813	-6.526	0.586	2165
R Precuneus	0.115	-0.032	-3.080	1.954	-6.912	0.752	2164
L Rostral anterior cingulate	0.111	-0.033	-1.084	0.680	-2.417	0.249	2140
**R Rostral anterior cingulate**	**<0.001**	**-0.070**	**-2.095**	**0.587**	**-3.247**	**-0.944**	**2144**

**L Rostral middle frontal**	**<0.001**	**-0.077**	**-11.100**	**2.893**	**-16.773**	**-5.427**	**2149**
R Rostral middle frontal	0.001	-0.065	-9.664	2.973	-15.494	-3.834	2160
L Superior frontal	0.010	-0.051	-7.911	3.067	-13.926	-1.897	2121
R Superior frontal	0.007	-0.054	-8.404	3.099	-14.481	-2.327	2124
L Superior parietal	0.077	-0.037	-4.601	2.596	-9.692	0.491	2098
R Superior parietal	0.056	-0.039	-4.928	2.577	-9.981	0.125	2115
L Superior temporal	0.233	-0.025	-2.154	1.807	-5.698	1.390	1892
**R Superior temporal**	**<0.001**	**-0.078**	**-5.885**	**1.546**	**-8.917**	**-2.853**	**2011**
L Supramarginal	0.028	-0.046	-5.214	2.375	-9.872	-0.556	2050
R Supramarginal	0.202	-0.027	-2.699	2.116	-6.849	1.451	2081
L Temporal pole	0.323	-0.021	-0.273	0.277	-0.816	0.269	2079
R Temporal pole	0.195	0.026	0.367	0.283	-0.189	0.923	2083
L Transverse temporal	0.937	-0.002	-0.026	0.331	-0.675	0.623	2177
R Transverse temporal	0.559	-0.012	-0.137	0.235	-0.597	0.323	2176

Results of regression analysis of duration of illness and cortical surface area in PD sample. L – Left, R – Right, ROI – region of interest. Banks STS – Banks of the superior temporal sulcus. **Boldface** denotes significant results after Bonferroni correction.

Supplementary table 14f: Results of regression analysis of duration of illness and subcortical volume – uncorrected for age

ROI Subcortical	*p*	*r*	b	SE	Lower CI	Upper CI	NPD
**L Amygdala**	**<0.001**	**-0.131**	**-7.298**	**1.043**	**-9.343**	**-5.252**	**2153**
**R Amygdala**	**<0.001**	**-0.126**	**-7.600**	**1.102**	**-9.761**	**-5.439**	**2147**
**L Caudate nucleus**	**<0.001**	**-0.107**	**-11.603**	**2.109**	**-15.739**	**-7.467**	**2172**
**R Caudate nucleus**	**<0.001**	**-0.089**	**-9.674**	**2.109**	**-13.810**	**-5.537**	**2176**
L Globus pallidus	0.929	-0.002	-0.107	1.205	-2.470	2.256	2059
R Globus pallidus	0.560	-0.010	-0.629	1.077	-2.742	1.484	2120
**L Hippocampus**	**<0.001**	**-0.094**	**-10.590**	**2.229**	**-14.961**	**-6.220**	**2128**
**R Hippocampus**	**<0.001**	**-0.094**	**-11.138**	**2.308**	**-15.665**	**-6.611**	**2159**
L Lateral ventricle	0.021	0.048	79.766	34.507	12.096	147.437	2197
R Lateral ventricle	0.009	0.055	83.643	31.992	20.903	146.382	2198
**L Nucleus accumbens**	**<0.001**	**-0.094**	**-2.770**	**0.540**	**-3.830**	**-1.711**	**2164**
R Nucleus accumbens	**<0.001**	**-0.082**	**-2.191**	**0.512**	**-3.195**	**-1.187**	**2138**
L Putamen	**<0.001**	**-0.095**	**-15.451**	**3.083**	**-21.498**	**-9.405**	**2047**
R Putamen	**<0.001**	**-0.074**	**-11.331**	**2.915**	**-17.048**	**-5.615**	**2103**
**L Thalamus**	**<0.001**	**-0.084**	**-18.775**	**3.893**	**-26.409**	**-11.141**	**2072**
**R Thalamus**	**<0.001**	**-0.089**	**-16.724**	**3.314**	**-23.224**	**-10.225**	**2128**

Results of regression analysis of duration of illness and subcortical volume in PD sample. L – Left, R – Right, ROI – region of interest. **Boldface** denotes significant results after Bonferroni correction.

Supplementary Table 15a: Cortical thickness results - MoCA regression analysis corrected for DURILL

ROI Thickness	*p*	*r*	b	SE	Lower CI	Upper CI	NPD
L Banks STS	0.003	0.076	0.006	0.002	0.002	0.010	940
R Banks STS	0.002	0.077	0.006	0.002	0.002	0.010	1005
L Caudal anterior cingulate	0.404	0.023	0.002	0.003	-0.003	0.008	1026
R Caudal anterior cingulate	0.418	0.021	0.002	0.003	-0.003	0.007	1025
**L Caudal middle frontal**	**<0.001**	**0.118**	**0.007**	**0.002**	**0.004**	**0.011**	**1034**
**R Caudal middle frontal**	**<0.001**	**0.100**	**0.006**	**0.002**	**0.003**	**0.009**	**1034**
L Cuneus	0.178	0.038	0.002	0.002	-0.001	0.005	939
R Cuneus	0.522	0.017	0.001	0.002	-0.002	0.004	929
L Entorhinal	0.047	0.055	0.007	0.003	<0.001	0.013	1017
R Entorhinal	0.034	0.059	0.008	0.004	0.001	0.015	1011
**L Frontal pole**	**<0.001**	**0.105**	**0.011**	**0.003**	**0.005**	**0.016**	**1031**
R Frontal pole	0.008	0.074	0.007	0.003	0.002	0.013	1030
**L Fusiform**	**<0.001**	**0.134**	**0.009**	**0.002**	**0.006**	**0.012**	**1012**
**R Fusiform**	**<0.001**	**0.140**	**0.010**	**0.002**	**0.006**	**0.013**	**1019**
L Inferior parietal	0.009	0.066	0.004	0.002	0.001	0.007	1006
**R Inferior parietal**	**0.001**	**0.085**	**0.005**	**0.002**	**0.002**	**0.008**	**1004**
**L Inferior temporal**	**<0.001**	**0.104**	**0.007**	**0.002**	**0.004**	**0.010**	**991**
**R Inferior temporal**	**<0.001**	**0.104**	**0.007**	**0.002**	**0.004**	**0.010**	**1007**
**L Insula**	**<0.001**	**0.114**	**0.007**	**0.002**	**0.004**	**0.011**	**1031**
**R Insula**	**<0.001**	**0.120**	**0.009**	**0.002**	**0.005**	**0.012**	**1028**
L Isthmus cingulate	0.034	0.058	0.004	0.002	<0.001	0.008	1031
R Isthmus cingulate	0.006	0.076	0.006	0.002	0.002	0.010	1031
L Lateral occipital	0.667	0.011	0.001	0.001	-0.002	0.003	1006
R Lateral occipital	0.027	0.056	0.003	0.001	<0.001	0.006	1007
L Lateral orbitofrontal	0.006	0.065	0.005	0.002	0.001	0.008	1025
**R Lateral orbitofrontal**	**<0.001**	**0.083**	**0.006**	**0.002**	**0.003**	**0.009**	**1026**
L Lingual	0.003	0.079	0.004	0.001	0.001	0.007	998
R Lingual	0.008	0.071	0.004	0.001	0.001	0.006	981
L Medial orbitofrontal	0.001	0.087	0.006	0.002	0.002	0.009	1019
R Medial orbitofrontal	0.004	0.069	0.005	0.002	0.002	0.009	1022
**L Middle temporal**	**<0.001**	**0.127**	**0.009**	**0.002**	**0.006**	**0.013**	**943**
**R Middle temporal**	**<0.001**	**0.097**	**0.007**	**0.002**	**0.004**	**0.011**	**1005**
L Paracentral	0.002	0.083	0.006	0.002	0.002	0.009	1028
R Paracentral	0.002	0.084	0.006	0.002	0.002	0.009	1026
L Parahippocampal	0.186	0.037	0.004	0.003	-0.002	0.010	1030
R Parahippocampal	0.003	0.080	0.008	0.003	0.003	0.013	1030
**L Pars opercularis**	**<0.001**	**0.091**	**0.006**	**0.002**	**0.003**	**0.009**	**1025**
R Pars opercularis	0.001	0.082	0.005	0.002	0.002	0.009	1023
L Pars orbitalis	0.062	0.050	0.004	0.002	<0.001	0.008	1025
R Pars orbitalis	0.027	0.057	0.004	0.002	<0.001	0.008	1024
**L Pars triangularis**	**<0.001**	**0.134**	**0.009**	**0.002**	**0.006**	**0.012**	**1023**
**R Pars triangularis**	**<0.001**	**0.101**	**0.006**	**0.002**	**0.003**	**0.009**	**1022**
L Pericalcarine	0.360	0.025	0.001	0.002	-0.002	0.005	940
R Pericalcarine	0.260	0.031	0.002	0.002	-0.001	0.005	912

**L Postcentral**	**<0.001**	**0.092**	**0.005**	**0.001**	**0.002**	**0.008**	**995**
R Postcentral	0.004	0.074	0.004	0.001	0.001	0.007	996
L Posterior cingulate	0.002	0.078	0.005	0.002	0.002	0.009	1032
R Posterior cingulate	0.009	0.067	0.005	0.002	0.001	0.008	1031
**L Precentral**	**<0.001**	**0.122**	**0.008**	**0.002**	**0.005**	**0.012**	**1018**
**R Precentral**	**<0.001**	**0.113**	**0.008**	**0.002**	**0.004**	**0.011**	**1021**
L Precuneus	0.001	0.079	0.005	0.001	0.002	0.008	1033
**R Precuneus**	**<0.001**	**0.093**	**0.006**	**0.001**	**0.003**	**0.009**	**1032**
**L Rostral anterior cingulate**	**<0.001**	**0.095**	**0.009**	**0.002**	**0.004**	**0.014**	**1022**
R Rostral anterior cingulate	0.236	0.032	0.003	0.003	-0.002	0.008	1025
**L Rostral middle frontal**	**<0.001**	**0.109**	**0.006**	**0.001**	**0.004**	**0.009**	**1026**
R Rostral middle frontal	0.004	0.067	0.004	0.001	0.001	0.007	1026
**L Superior frontal**	**<0.001**	**0.097**	**0.006**	**0.002**	**0.003**	**0.009**	**1012**
**R Superior frontal**	**<0.001**	**0.101**	**0.006**	**0.002**	**0.003**	**0.009**	**1012**
L Superior parietal	0.015	0.062	0.004	0.001	0.001	0.007	1003
R Superior parietal	0.009	0.065	0.004	0.001	0.001	0.007	1017
**L Superior temporal**	**<0.001**	**0.114**	**0.008**	**0.002**	**0.004**	**0.011**	**904**
**R Superior temporal**	**<0.001**	**0.112**	**0.008**	**0.002**	**0.005**	**0.011**	**979**
L Supramarginal	0.004	0.075	0.005	0.002	0.002	0.008	997
R Supramarginal	0.005	0.073	0.005	0.002	0.001	0.008	1005
L Temporal pole	0.029	0.064	0.008	0.004	0.001	0.015	971
R Temporal pole	0.004	0.084	0.010	0.004	0.003	0.017	999
L Transverse temporal	0.004	0.081	0.007	0.002	0.002	0.011	1036
R Transverse temporal	0.001	0.093	0.008	0.002	0.003	0.013	1035

Results of regression analysis of duration of illness and cortical thickness in PD sample. L – Left, R – Right, ROI – region of interest. Banks STS – Banks of the superior temporal sulcus. **Boldface** denotes significant results after Bonferroni correction.

Supplementary Table 15b: Cortical surface area results - MoCA regression analysis corrected for DURILL

ROI Thickness	*p*	*r*	b	SE	Lower CI	Upper CI	NPD
L Banks STS	0.011	0.081	4.12	1.62	0.94	7.30	940
R Banks STS	0.253	0.034	1.61	1.41	-1.15	4.37	1005
L Caudal anterior cingulate	0.055	0.058	2.75	1.43	-0.05	5.55	1026
R Caudal anterior cingulate	0.069	0.054	2.92	1.60	-0.22	6.06	1025
L Caudal middle frontal	0.069	0.055	6.26	3.44	-0.49	13.01	1035
R Caudal middle frontal	0.388	0.026	2.90	3.35	-3.68	9.48	1035
L Cuneus	0.797	0.008	0.52	2.02	-3.45	4.49	939
R Cuneus	0.509	0.021	1.44	2.18	-2.83	5.70	929
L Entorhinal	0.873	-0.005	-0.15	0.92	-1.95	1.65	1017
R Entorhinal	0.797	0.008	0.22	0.85	-1.45	1.89	1012
L Frontal pole	0.370	0.024	0.28	0.31	-0.33	0.90	1031
R Frontal pole	0.155	0.040	0.59	0.42	-0.22	1.41	1030
L Fusiform	0.898	0.004	0.44	3.47	-6.36	7.25	1013
R Fusiform	0.750	0.010	1.06	3.33	-5.48	7.60	1018
L Inferior parietal	0.133	0.045	8.04	5.34	-2.44	18.53	1006
**R Inferior parietal**	**<0.001**	**0.116**	**22.92**	**5.99**	**11.16**	**34.67**	**1004**

L Inferior temporal	0.462	0.023	3.15	4.28	-5.25	11.56	991
R Inferior temporal	0.288	0.032	4.01	3.77	-3.39	11.41	1007
L Insula	0.487	0.020	1.44	2.07	-2.63	5.51	1031
R Insula	0.622	-0.014	-1.23	2.50	-6.14	3.67	1028
L Isthmus cingulate	0.104	0.047	2.45	1.51	-0.51	5.40	1031
R Isthmus cingulate	0.591	-0.016	-0.77	1.44	-3.59	2.04	1031
L Lateral occipital	0.856	0.005	0.96	5.27	-9.38	11.30	1006
R Lateral occipital	0.012	0.074	14.32	5.70	3.13	25.50	1007
L Lateral orbitofrontal	0.341	0.029	2.38	2.50	-2.52	7.29	1026
R Lateral orbitofrontal	0.214	0.037	3.41	2.74	-1.97	8.79	1027
L Lingual	0.238	0.037	4.57	3.87	-3.02	12.16	998
R Lingual	0.398	0.026	3.27	3.86	-4.32	10.85	981
L Medial orbitofrontal	0.430	0.024	1.63	2.07	-2.42	5.69	1018
R Medial orbitofrontal	0.625	-0.015	-0.89	1.83	-4.47	2.69	1021
L Middle temporal	0.156	0.044	5.17	3.64	-1.98	12.31	943
R Middle temporal	0.142	0.044	5.25	3.57	-1.76	12.26	1005
L Paracentral	0.962	0.001	0.08	1.65	-3.17	3.33	1028
R Paracentral	0.537	-0.019	-1.31	2.13	-5.49	2.86	1026
L Parahippocampal	0.124	0.046	1.47	0.95	-0.40	3.34	1030
R Parahippocampal	0.138	0.045	1.40	0.94	-0.45	3.24	1030
**L Pars opercularis**	**<0.001**	**0.106**	**7.99**	**2.27**	**3.54**	**12.45**	**1025**
R Pars opercularis	0.236	0.036	2.34	1.97	-1.53	6.20	1023
L Pars orbitalis	0.016	0.070	1.84	0.76	0.35	3.33	1025
R Pars orbitalis	0.078	0.052	1.68	0.95	-0.19	3.55	1024
L Pars triangularis	0.043	0.062	3.62	1.79	0.11	7.13	1023
R Pars triangularis	0.295	0.032	2.35	2.24	-2.05	6.74	1022
L Pericalcarine	0.848	-0.006	-0.50	2.62	-5.65	4.64	940
R Pericalcarine	0.532	-0.020	-1.61	2.58	-6.66	3.45	912
L Postcentral	0.207	0.039	5.11	4.04	-2.83	13.04	995
R Postcentral	0.092	0.052	6.89	4.09	-1.14	14.91	996
L Posterior cingulate	0.226	0.036	2.51	2.07	-1.56	6.58	1032
R Posterior cingulate	0.376	0.025	1.46	1.65	-1.78	4.70	1031
L Precentral	0.300	0.031	4.62	4.45	-4.12	13.35	1018
R Precentral	0.014	0.073	11.26	4.59	2.24	20.27	1021
L Precuneus	0.004	0.085	11.31	3.93	3.59	19.03	1033
R Precuneus	0.113	0.047	6.68	4.21	-1.59	14.95	1032
L Rostral anterior cingulate	0.671	-0.013	-0.68	1.61	-3.84	2.47	1022
R Rostral anterior cingulate	0.442	0.022	1.00	1.30	-1.55	3.55	1025
L Rostral middle frontal	0.627	0.014	3.05	6.27	-9.25	15.34	1026
R Rostral middle frontal	0.321	0.029	6.37	6.42	-6.22	18.96	1026
L Superior frontal	0.963	-0.001	-0.31	6.72	-13.50	12.88	1013
R Superior frontal	0.209	0.037	8.74	6.95	-4.91	22.39	1012
L Superior parietal	0.001	0.099	18.46	5.60	7.46	29.45	1003
R Superior parietal	0.003	0.090	16.35	5.42	5.72	26.99	1017
L Superior temporal	0.208	0.039	4.97	3.94	-2.77	12.70	904
R Superior temporal	0.524	0.019	2.12	3.33	-4.41	8.65	979
L Supramarginal	0.003	0.090	16.10	5.38	5.54	26.67	997
R Supramarginal	0.477	0.022	3.40	4.79	-5.99	12.80	1005
L Temporal pole	0.380	0.027	0.56	0.64	-0.69	1.80	971
R Temporal pole	0.806	-0.007	-0.16	0.63	-1.40	1.09	999
L Transverse temporal	0.023	0.068	1.72	0.76	0.24	3.21	1036

R Transverse temporal	0.066	0.056	0.93	0.50	-0.06	1.92	1035

Results of regression analysis of duration of illness and cortical thickness in PD sample. L – Left, R – Right, ROI – region of interest. Banks STS – Banks of the superior temporal sulcus. **Boldface** denotes significant results after Bonferroni correction.

Supplementary Table 15c: Subcortical volume results - MoCA regression analysis corrected for DURILL

ROI Subcortical	*p*	*r*	b	SE	Lower CI	Upper CI	NPD
**L Amygdala**	**<0.001**	**0.123**	**9.89**	**2.10**	**5.76**	**14.01**	**1047**
**R Amygdala**	**<0.001**	**0.101**	**8.72**	**2.21**	**4.38**	**13.06**	**1049**
L Caudate nucleus	0.505	0.019	3.22	4.83	-6.26	12.70	1044
R Caudate nucleus	0.680	0.012	2.04	4.94	-7.66	11.75	1045
L Globus pallidus	0.877	0.004	0.39	2.55	-4.61	5.40	1023
R Globus pallidus	0.518	0.016	1.49	2.31	-3.04	6.03	1040
**L Hippocampus**	**<0.001**	**0.104**	**16.37**	**4.28**	**7.97**	**24.77**	**1033**
**R Hippocampus**	**<0.001**	**0.112**	**19.14**	**4.50**	**10.31**	**27.97**	**1046**
**L Lateral ventricle**	**<0.001**	**-0.120**	**-300.98**	**74.52**	**-447.21**	**-154.75**	**1051**
**R Lateral ventricle**	**<0.001**	**-0.116**	**-273.45**	**69.39**	**-409.62**	**-137.28**	**1051**
L Nucleus accumbens	0.031	0.056	2.50	1.16	0.23	4.77	1048
R Nucleus accumbens	0.173	0.038	1.49	1.09	-0.65	3.63	1049
L Putamen	0.008	0.071	16.10	6.10	4.13	28.07	1024
R Putamen	0.055	0.054	11.38	5.92	-0.24	23.01	1039
L Thalamus	0.111	0.043	11.84	7.43	-2.74	26.42	1009
R Thalamus	0.145	0.038	9.12	6.26	-3.16	21.40	1023

Results of regression analysis of duration of illness and subcortical volume in PD sample. L – Left, R – Right, ROI – region of interest. **Boldface** denotes significant results after Bonferroni correction.

Supplementary figure S1: World map participating sites

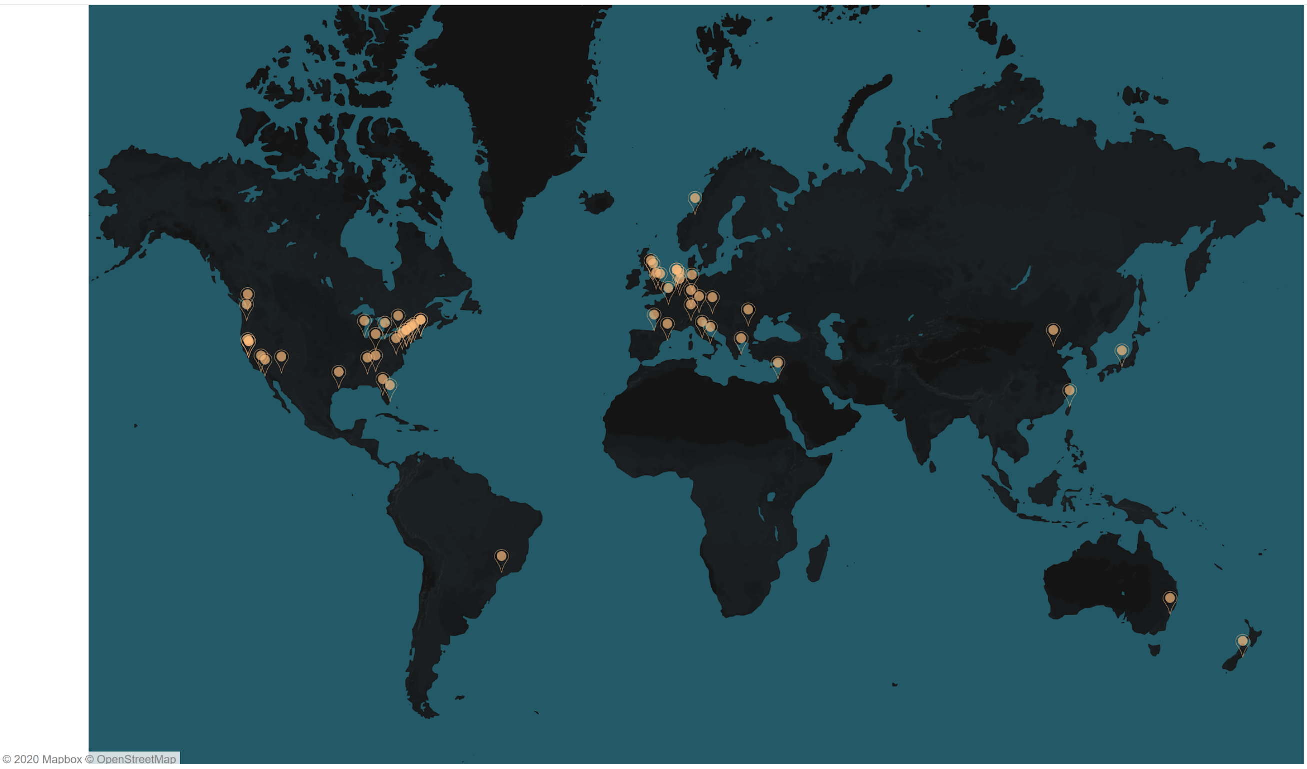

Supplement: Supplementary file 1 — Appendix S1. Supplementary Information [file MDS-36-2583-s001.pdf]
